# Atypical Outcome of the Giese Reaction with Halogenated Enones

**DOI:** 10.1021/acs.joc.6c00649

**Published:** 2026-06-15

**Authors:** Vojtěch Kundera, Judit E. Šponer, Jakub Švenda

**Affiliations:** † Department of Chemistry, Faculty of Science, 37748 Masaryk University, Kamenice 5, Brno 625 00, Czech Republic; ‡ Institute of Biophysics of the Czech Academy of Sciences, Brno 612 65, Czech Republic; § International Clinical Research Center, St. Anne’s University Hospital, Brno 656 91, Czech Republic

## Abstract

An atypical pathway in the Giese reaction is being reported. Photoredox-mediated 1,4-addition of α-amino radicals onto an α′-fluorinated enone was found to proceed with a loss of fluoride and retention of the acceptor C=C bond. Experimental observations and computational analysis are consistent with the initially formed fluorinated α-carbonyl radical intermediate undergoing rapid deprotonation rather than the typically observed one-electron reduction. This underexplored reactivity may aid the development of an oxidative Giese reaction.

## Graphical abstract

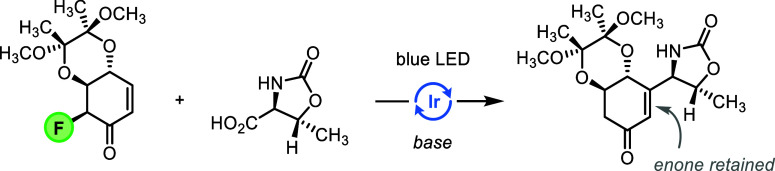

## 1 Introduction

Efficient chemical synthesis of organic molecules depends critically on fragment-coupling reactions. Those with a broad scope and predictable selectivity are particularly valuable and have been adopted in the synthesis of complex molecules.^1,2^ 1,4-Additions of carbon-centered free radicals onto electron-deficient alkenes (the Giese reaction^3,4^ ) made challenging Csp3–Csp3 bond disconnections possible.^5–7^

Recently, we used photoredox catalysis to promote 1,4-additions of α-amino radicals onto chiral enones to prepare natural and modified bactobolin antibiotics.^8^ Scheme 1 illustrates a photoredox Giese reaction between enone **1** and oxazolidinone carboxylic acid **2**, which we used in the synthesis of (+)-actinobolin, yielding two diastereomeric adducts, **4** and **5**.^8^ The mechanism of this transformation is outlined in Scheme 2 and follows a previous proposal.^9^ Briefly, single-electron oxidation of the carboxylate anion **6** by the photoexcited state of iridium­(III) catalyst **3** promotes decarboxylation with release of the α-amino radical **7**. This highly nucleophilic radical undergoes facile 1,4-addition onto enone **1** to give the α-carbonyl radical intermediate **8**. The photoredox catalytic cycle is closed after a single-electron reduction of **8** by iridium­(II) species, leading to the corresponding anion (enolate **9**) and ultimately products of enolate protonation, compounds **4** and **5**. Regenerated iridium­(III) complex **3** re-enters the catalytic cycle.

**Scheme 1:**
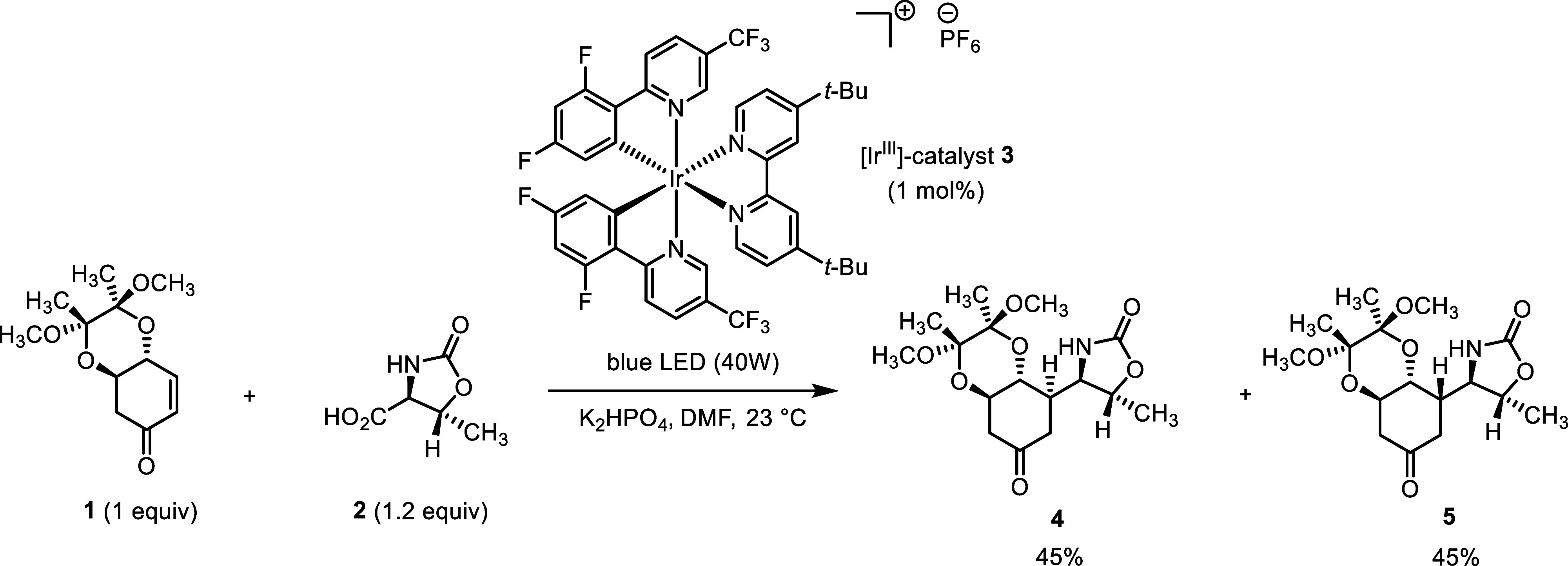
Standard Reaction Outcome of the Photoredox-Promoted Coupling of Enone 1 and Oxazolidinone 2

**Scheme 2:**
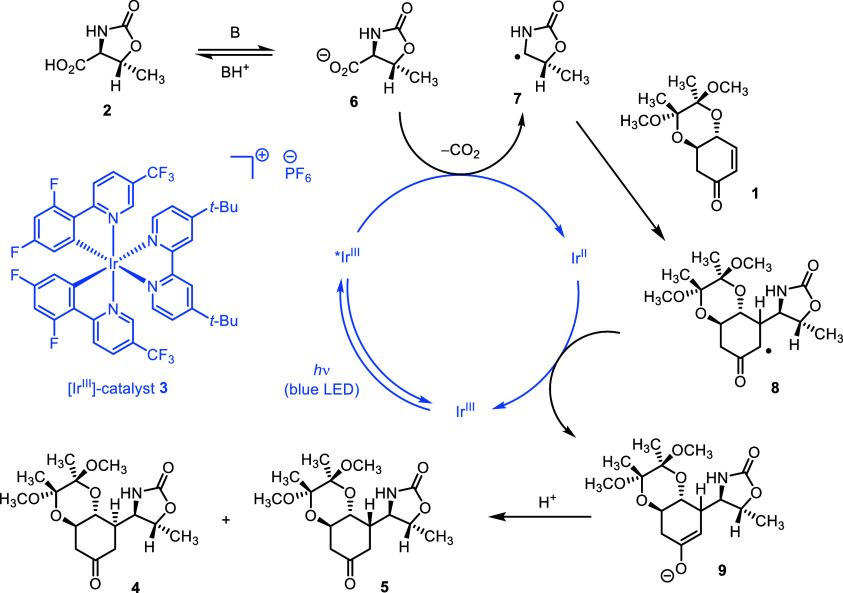
Reaction Mechanism for the Photoredox Catalysis of the Coupling between 1 and 2

Various enones were found to participate in this fragment-coupling reaction and follow the outcome described in Scheme 1. Nonetheless, an unexpected mode of reactivity emerged upon examining α′-halogenated enones.^10^ As shown in Scheme 3, coupling oxazolidinone **2** and α′-fluorinated enone **10** (2.0 equiv) afforded atypical product **11** as the major species (43% isolated yield), with less than 5% of expected 1,4-adducts **4** and **5** observed. The same product (**11**) was also observed with α′-chlorinated and α′-acetoxylated analogs of enone **10**, albeit only in 15–26% yield (see Supporting Information). Product **11** is atypical in that the enone C=C bond is retained, which, to our knowledge, is not a common mode of reactivity in the context of the Giese reaction of carbon-centered free radicals.^11^ This paper aims to deconvolute the mechanistic details of this unusual reaction outcome.

**Scheme 3:**
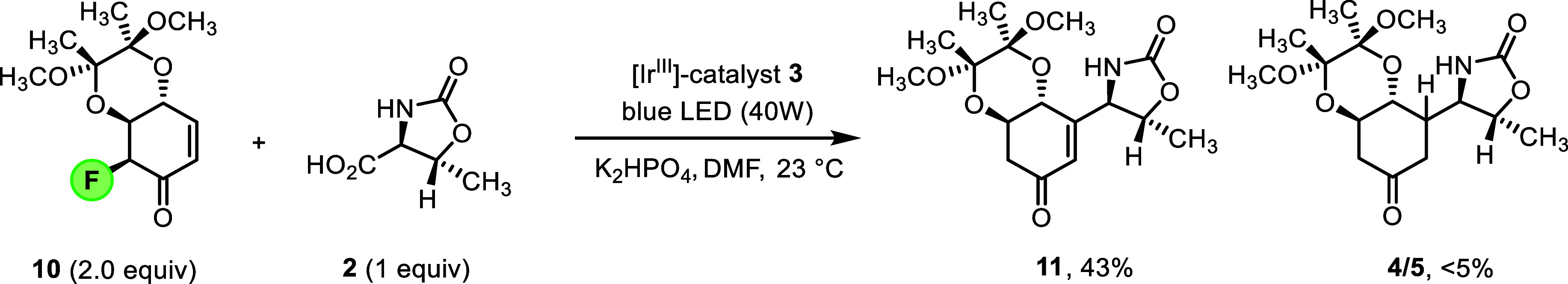
Atypical Reaction Outcome with Fluorinated Enone 10

## 2 Results and Discussion

### 2.1 Preparation of Fluorinated Enones

Fluorinated enones employed in this study were prepared from (−)-quinic acid in six to eight steps (Scheme 4). Formation of *tert*-butyldimethylsilyl enol ether from known enone **1**,^12^ followed by treatment with Selectfluor, gave epimeric monofluorinated products **10** (64%) and **12** (7%). The major epimer **10**, containing a pseudoaxial C–F bond, matched the previously reported NMR data,^13^ confirmed here beyond doubt by X-ray crystallographic analysis (Scheme 4). The minor epimer **12** was characterized upon careful chromatography.

**Scheme 4:**
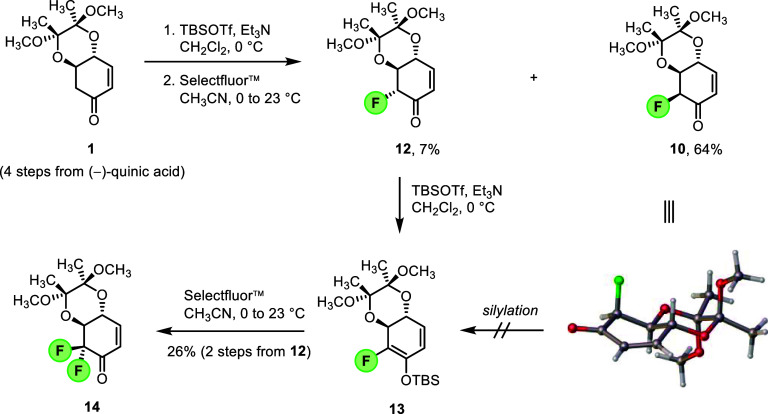
Synthesis of Fluorinated Enones

The epimeric monofluorinated enones **10** and **12** displayed noticeably different reactivity in the subsequent α′-functionalization. Only epimer **12**, having a C–F bond in the pseudoequatorial orientation, underwent formation of the corresponding silyl enol ether and subsequent electrophilic fluorination to afford gem-difluorinated enone **14**. The epimeric enone **10** failed to yield the silyl enol ether **13** under various conditions. This contrasting reactivity agrees with observations by others^14^ and can be understood through the lower kinetic acidity of the pseudoequatorial C–H bond in the conformationally rigid enone **10** (see the crystal structure in Scheme 4). Fluorinated enones **10**, **12**, and **14** were employed as radical acceptors in the present study.

### 2.2 Enone with a Pseudoaxially Oriented C–F Bond

The observed formation of atypical product **11** with fluorinated enone **10** (Scheme 3) merges a reduction (defluorination) and an oxidation (retention of the enone C=C bond). The overall process may therefore be viewed as redox-neutral. Given that radical dehalogenations of α-fluoro carbonyls are known in the literature,^15–19^ we first investigated whether the events of defluorination and enone C=C bond retention are intertwined.

Were defluorination to occur prior to the 1,4-addition, the product mixture would have to contain significant amounts of the standard products **4** and **5** (less than 5% were detected by NMR). Separately or in a competition experiment, the coupling of oxazolidinone **2** to the fluorinated enone **10** was faster relative to the reference nonfluorinated enone **1** (see Supporting Information). These observations place the defluorination step after the radical 1,4-addition. Following the mechanism in Scheme 2, a fluorinated analog of α-carbonyl radical **8** is therefore implied. The presence of fluorine in the α′ position of radical **8** should render its single-electron reduction by iridium­(II) more thermodynamically favorable (**8** → **9** in Scheme 2).^20^ The corresponding enolate (analogous to **9**), having fluorine in the α′ position, is principally set up for the Favorskii rearrangement^21^ as seen with enolizable ketones. There, extrusion of the fluoride anion leads to a cyclopropanone intermediate, followed by a ring opening/contraction. While we did not observe products of ring contraction in the product mixtures using enone **10**, it is worth noting that cyclopropanones may alternatively undergo fragmentation to enones, as reported in the case of vicarious elimination on α′-brominated cyclohexanone **15** leading to enone **18** (Scheme 5a).^22^ An alternative mechanism for an analogous elimination was reported on the brominated podocarpane derivative **19**, wherein a base-promoted 1,4-elimination led to enone **22** (Scheme 5b).^23^ In principle, both mechanistic scenarios described in Scheme 5 may be used to explain the formation of the atypical product **11** we observe. Additional experiments were performed to assess these mechanistic possibilities.

**Scheme 5:**
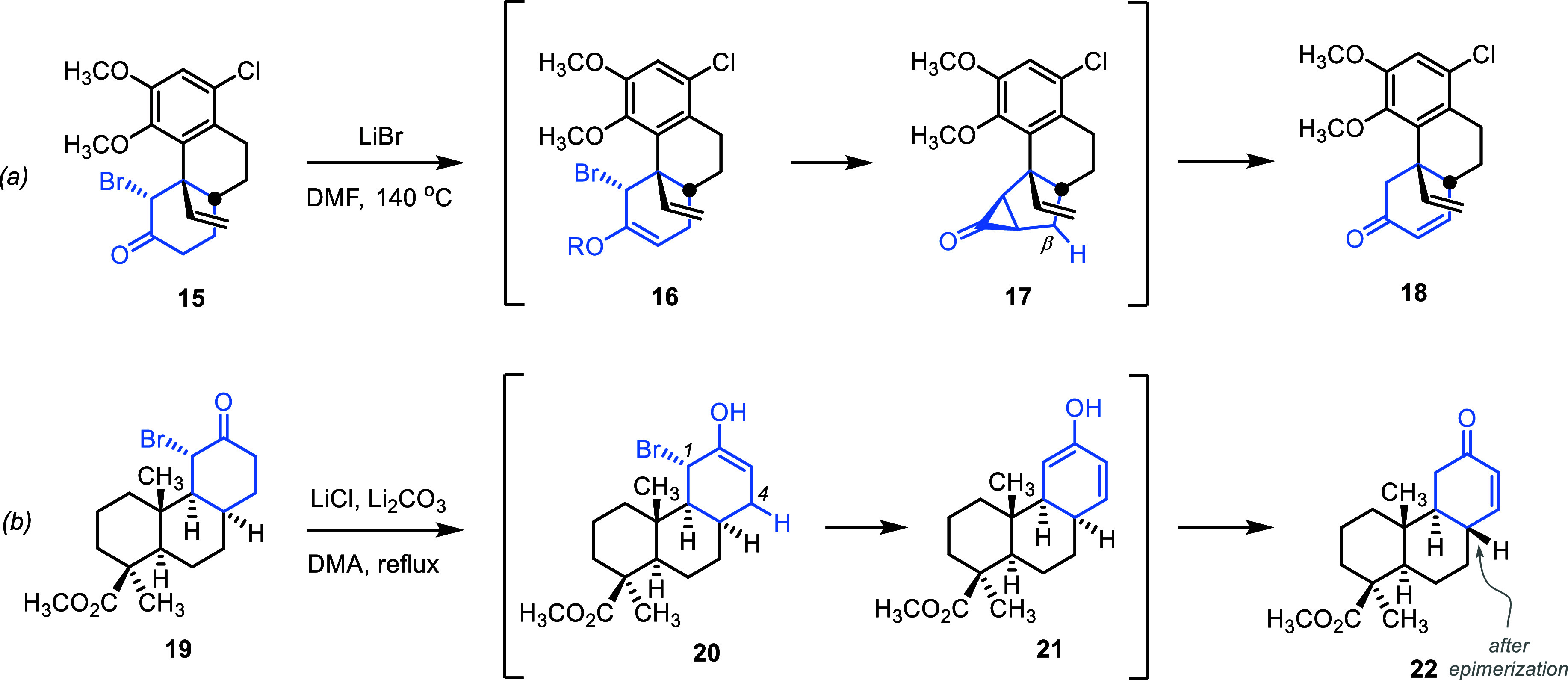
Previously Reported Mechanisms for the Conversion of α′-Halogenated Ketones into Enones

The moderate isolated yield of the atypical product **11** (43% yield using 2.0 equiv of enone **10**) led us to inquire into the mass balance of the reaction. To that end, we carried out a series of experiments in DMF using iridium­(III) complex **3** or 4-CzIPN (2,4,5,6-tetrakis­(9*H*-carbazol-9-yl)­isophthalonitrile, **23**) as photocatalysts, with early and late stop of the reaction (Scheme 6). Upon stopping the reaction early (ca. 40% conversion of enone **10**, 4-CzIPN (**23**) as photocatalyst), we found by ^19^F NMR analysis of the crude mixture that a significant fraction of the coupled product still contained fluorine in the axial position (enone **10**: −206.5 ppm (dd, *J* = 52.0, 34.7 Hz), detected fluorinated product: −205.8 ppm (dd, *J* = 52.0, 34.0 Hz) in ^19^F NMR). We isolated this fluorinated product to unambiguously determine its structure by NMR and X-ray analysis (structure **24** in Scheme 6). An analogous outcome leading to **24** was observed with iridium­(III) photocatalyst **3** upon the early stop (entries 1,2). At high enone conversions (late stop, entry 3), the atypical product **11** without fluorine predominated. Interestingly, employing higher amount of the fluorinated enone **10** (relative to acid **2**) increased the fraction of the fluorinated product **24** and the combined overall yield of products **24** and **11** (ca. 70% combined yield obtained from a 4:1 molar ratio of enone **10** to acid **2**, entry 8). Product **11** was the only isolable product when excess acid **2** was used (entry 9).

**Scheme 6:**
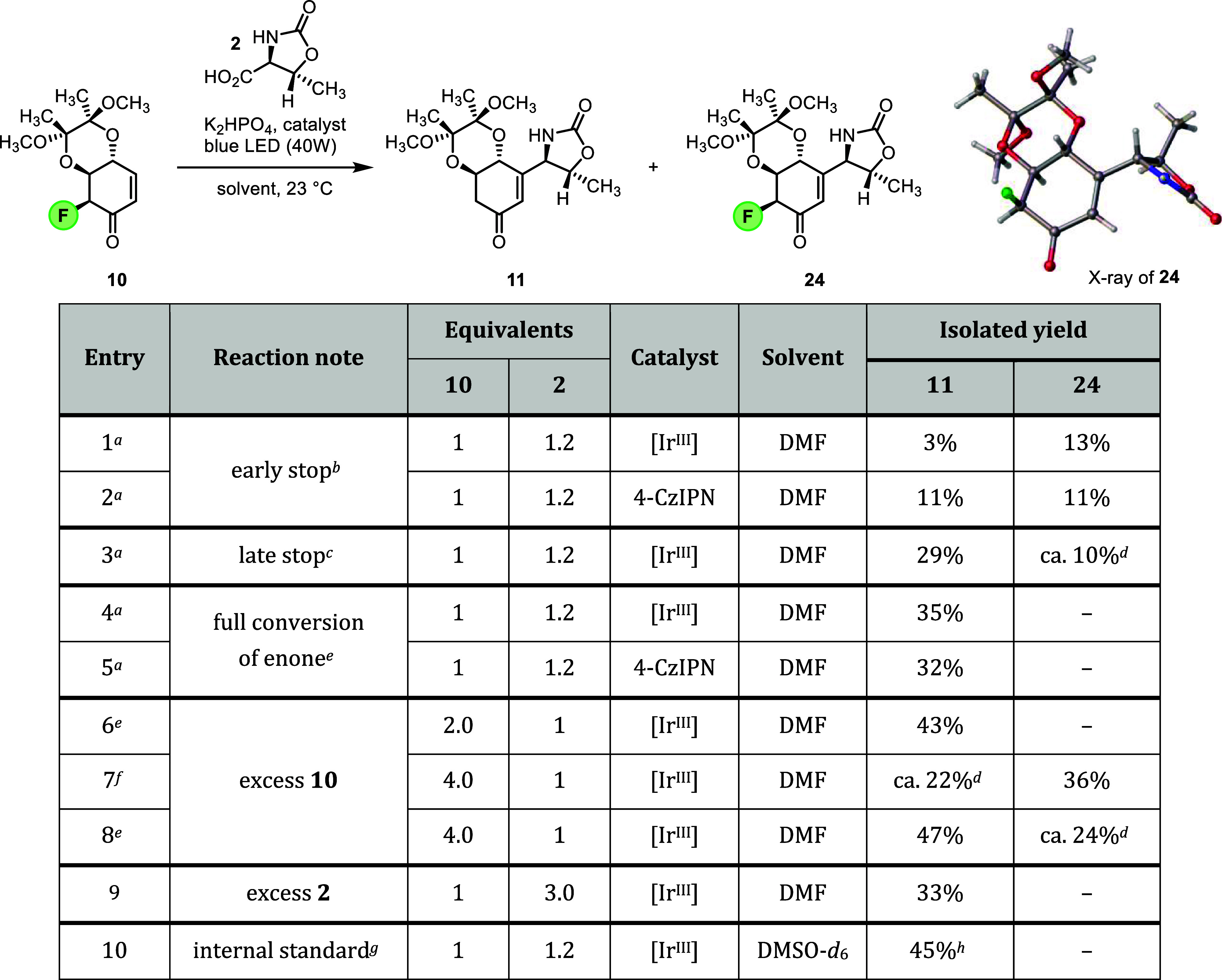
Coupling between Enone 10 and Oxazolidinone 2 under Various Reaction Conditions^a^ ^,^ ^b^ ^,^ ^c^ ^,^ ^d^ ^,^ ^e^ ^,^ ^f^ ^,^ ^g^ ^,^ ^h^

a Standard conditions: enone **10**, acid **2**, 1.2 equiv K_2_HPO_4_, and 1 mol % of photocatalyst.
b Unreacted enone **10** observed as the major component of the reaction mixture (TLC analysis).
c Enone **10** almost fully consumed (TLC analysis).
d Indicated yield is approximate due to inseparable impurities.
e Reaction was allowed to proceed overnight (16–19 h).
f Reaction time was 1 h.
g 1,3,5-Trimethoxybenzene (0.35 equiv) was used as an internal NMR standard.
h Yield determined by ^1^H NMR against 1,3,5-trimethoxybenzene as the internal standard. Abbreviations: 4-CzIPN = 2,4,5,6-tetrakis­(9*H*-carbazol-9-yl)­isophthalonitrile, [Ir^III^] = catalyst **3**.

To rule out that workup or chromatography is responsible for the lower isolated yield of the atypical products **11** and **24**, we performed additional experiments in DMSO-*d*_6_ and analyzed the crude product mixtures against an internal standard (1,3,5-trimethoxybenzene, Scheme 6, entry 10). Although the coupled defluorinated product **11** remained the major species after 2 and 1/2 h using [Ir^III^]-catalyst **3**, the NMR yield of product **11** relative to the internal standard was only 45%; a good correlation with the isolated yield of **11**. Inspection of the crude reaction mixture in DMSO-*d*_6_ by sensitive ^19^F NMR analysis reported the presence of other unidentified fluorine-containing species (see Supporting Information).

The oxidized fluorinated product **24** is reactive under our standard reaction conditions. When a DMSO-*d*_6_ solution of **24** and oxazolidinone acid **2** (1.2 equiv) was irradiated in the presence of [Ir^III^]-catalyst **3**, a rapid conversion to **11** ensued (<20 min, ca. 55% yield by NMR, Scheme 7). Despite some intractable decomposition, the experiment established the fluorinated product **24** as a viable precursor of final product **11**; the oxazolidinone carboxylic acid **2** is the likely stoichiometric reductant in the process. Product **11** was stable under these conditions.

**Scheme 7:**
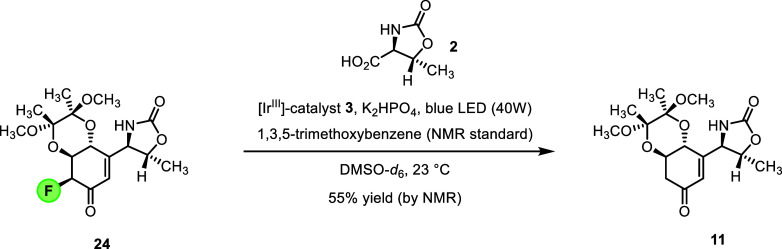
Defluorination of Oxidized Product 24 under the Standard Reaction Conditions

The above observations indicate that the steps of enone retention and defluorination are independent, and that the moderate yield of **11** results from intractable decomposition during the formation of **24** and its subsequent defluorination.

### 2.3 Enone with a Pseudoequatorially Oriented C–F Bond

The above experiments demonstrated that the presence of fluorine in the α′ position (enone **10**) alters the typical reaction outcome. To probe a potential stereoelectronic effect, we examined the epimeric enone **12** (see Scheme 4 for preparation) bearing a pseudoequatorially oriented C–F bond. Under the conditions suitable for NMR monitoring ([Ir^III^]-catalyst **3**, DMSO-*d*_6_, 1,3,5-trimethoxybenzene as internal standard), a 1.3:1 mixture of the 1,4-addition products **25** and **26** (ca. 70% yield by NMR) was observed (Scheme 8). The equatorial C–F bond was assigned by ^19^F NMR. The atypical product **11** was detected as a minor species (ca. 9% yield by NMR). We attribute the retention of fluorine in products **25** and **26** to the equatorial orientation of the carbon–fluorine bond.^24^ More importantly, the different reaction outcomes with enones **10** and **12** point to a stereoelectronic effect of the C–F bond. The distinct enolization propensities of enones **10** and **12**, as described in Scheme 4, do not rationalize this C–F bond effect.

**Scheme 8:**
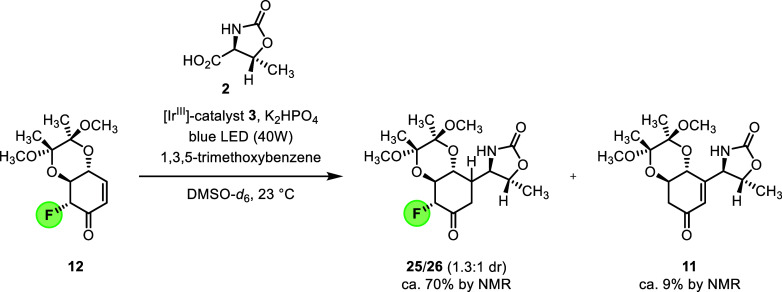
Reaction Outcome with Fluorinated Enone 12

Attempted coupling of oxazolidinone **2** to the difluorinated enone **14** did not provide further insight into the reaction, as only an intractable mixture of products was observed (enone **14** fully consumed).

### 2.4 Discussion of the Reaction Mechanism

To postulate a mechanism for the atypical reaction outcome with fluorinated enone **10** (Scheme 3), we weighed all experimental observations described above and the relevant literature. Contrary to initial considerations, the detection and isolation of the fluorinated product **24** proved that retaining the enone C=C bond does not require C–F bond cleavage, at least not within the same molecule. This effectively ruled out the involvement of the mechanisms based on the cyclopropanone fragmentation or base-mediated 1,4-elimination (Scheme 5). Instead, our data support a pathway proceeding initially to the oxidized fluorinated product **24**, followed by reduction to the final atypical product **11**.

To postulate how the oxidized fluorinated product **24** might form, we queried the mechanism of photoredox-mediated Giese reaction outlined in Scheme 2. Because the defluorination occurs after the radical 1,4-addition, the fluorinated α-carbonyl radical **27** is supported as an early reaction intermediate (Scheme 9). A single-electron reduction of **27** by iridium­(II) to form enolate **30** (in gray) would be expected to be more favorable due to the fluorine effect,^20^ but does not lead to the formation of product **24**.

**Scheme 9:**
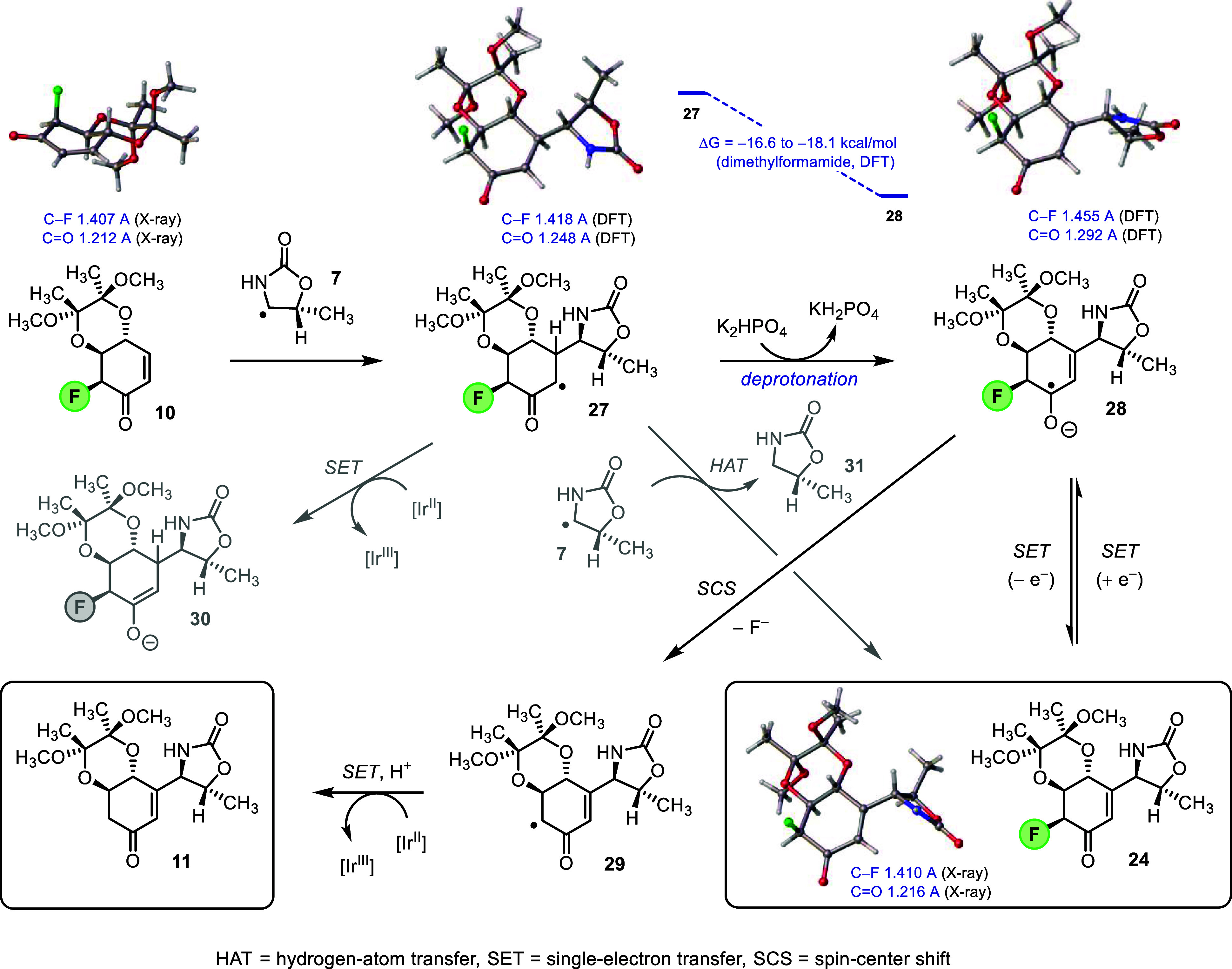
Proposed Reaction Mechanism for the Formation of Atypical Products 24 and 11

Hydrogen-atom transfer (HAT, in gray) represents a direct pathway from intermediate **27** to product **24**. Such a process is predicted to be highly thermodynamically favored with α-amino radical **7** (Δ*G* = −52.4 kcal/mol in DMF), yet kinetically unlikely given the bimolecular coupling of two radicals under catalytic conditions. Moreover, experiments with β-deuterated fluorinated enone **10**-*d* (≥95% deuteration, see Supporting Information for synthesis) and oxazolidinone acid **2** in DMSO-*d*_6_ gave no detectable amounts of decarboxylated oxazolidinone with or without deuterium (**31** or **31**-*d*) to support the HAT pathway (see Supporting Information).

An alternative mechanistic possibility for the **27** → **24** conversion involves initial deprotonation of **27** by dipotassium hydrogen phosphate to yield **28**. Increased thermodynamic acidity of neutral carbon-centered radicals has been noted^25^ and shown to be operative, for example, in radical trifluoromethylation of phenanthridine.^26^ For α-carbonyl radicals specifically, the dramatic acidification of β-hydrogens plays a key role in the enzymatic mechanism of (*R*)-2-hydroxyacyl-CoA dehydratases.^27,28^ According to our DFT calculations, energy-minimized **27** adopts a half-chair conformation with a lengthened C=O bond due to delocalization of the radical (one epimer of **27** shown). The deprotonation step is predicted to be highly favorable in DMF for both epimers of **27** (calculated Δ*G* = −16.6 and −18.1 kcal/mol, respectively) and about twice as favorable as for the nonfluorinated counterparts (calculated Δ*G* = −8.4 and −9.5 kcal/mol). The resulting ketyl radical anion **28** can evolve in two main ways: (1) a spin-center shift (SCS) pathway^24,29^ with elimination of fluoride to give **29** and ultimately **11**, or (2) a single-electron oxidation to give **24**.

The first pathway borrows from the mechanism of defluorination of α-fluoro carbonyls.^24^ The axial C–F bond is significantly lengthened in the energy-minimized structure of **28**, facilitating expulsion of fluoride anion (SCS pathway). The resulting α-carbonyl radical **29** undergoes a single-electron reduction and protonation to give the final product **11**. The carboxylate anion may serve as the stoichiometric reductant (Ir­(III) → Ir­(II) cycling) in the **29** → **11** conversion.

The second pathway requires an electron loss from the ketyl radical anion **28**. Since the reaction was performed in deoxygenated solvent under an inert atmosphere and a sacrificial stoichiometric oxidant was not included, there should be a theoretical limit to the amount of oxidized **24** that can be obtained. In one possibility, the unreacted fluorinated enone **10** may act as a single-electron oxidant to quench the ketyl radical anion **28**. This would be consistent with the observed increase of intermediate **24** when using excess fluorinated enone **10** (see entries 7 and 8, Scheme 6). The process, however, lacks a clear thermodynamic driving force (calculated Δ*G* = +2.6 kcal/mol in DMF), unless paired with the subsequent irreversible defluorination. Alternatively, **28** could transfer one electron to **29**, which is predicted to be highly favorable in DMF (calculated Δ*G* = −22.2 kcal/mol), but seems unlikely under the catalytic conditions. An electron transfer from **28** to trace amounts of oxygen cannot be ruled out.

The partitioning of intermediate **28** via the two routes (**28** → **24** and **28** → **29**, respectively) is likely. Our data suggest that **28 → 24** is a significant pathway in DMF, as early stop experiments and those using excess fluorinated enone **10** favored the formation of **24** over **11**. The **28** ↔ **24** redox interconversion is assumed to be reversible (cf. Scheme 7), and strong reductants such as the iridium­(II) form of catalyst **3** (*E*_1/2_^red^ −1.37 V vs SCE) may repopulate intermediate **28** from **24** to enter defluorination via the SCS pathway (**28** → **29**/**11**).

Finally, attempted coupling between **2** and **10** in the presence of 1,1-diphenylethylene (radical scavenger), shut down the Giese reaction pathway by capturing the oxazolidinone radical **7** and the α′-carbonyl radical in defluorination of enone **10** (see Supporting Information).

Overall, the experiments and calculations described above helped frame a mechanism for the atypical photoredox-mediated Giese reaction with fluorinated enone **10** (Scheme 9). Key to the proposal is the recognition that α-carbonyl radical intermediates, cemented in the mechanism of the Giese reaction with α,β-unsaturated carbonyls, are notably acidic and that their deprotonation can compete with the typically observed single-electron reduction. Calculations have shown that the presence of a pseudoaxially oriented fluorine in the α′ position of enone **10** favors the deprotonation step thermodynamically. In addition, we speculate that the properly oriented C–F bond may interfere with the one-electron reduction of the α-carbonyl radical (SET pathway), further steering the reaction pathway toward elimination. The outcomes with α′-chlorinated and α′-acetoxylated analogs of **10** (see Supporting Information), despite being lower yielding, indicate that the effect is not limited to fluorine.

## 3 Conclusions

In this work, we reported a case of the Giese reaction with α′-functionalized enones that proceeded with retention of the acceptor C=C bond. This atypical reaction outcome was probed experimentally and computationally using α′-fluorinated enone **10**. The results point to a mechanism wherein the initially formed α-carbonyl radical intermediate is deprotonated in preference to the normally observed one-electron reduction. While demonstrated here for the specific case of a conformationally rigid class of enones, ready deprotonation of α-carbonyl radicals is known to be used by (*R*)-2-hydroxyacyl-CoA dehydratases on acyclic substrates.^27,28^ To our knowledge, such an approach is unexplored in the development of an oxidative Giese reaction.

## Supplementary Material



## Data Availability

All data underlying this study are available in the published article and its Supporting Information.

## Notes

Detailed experimental procedures, X-ray crystallographic data, DFT calculations, and copies of NMR spectra are provided (PDF)
