# Supplementary material for: Atypical Outcome of the Giese Reaction with Halogenated Enones
Source: J Org Chem. 2026 Jun 15;91(25):8616–23. doi: 10.1021/acs.joc.6c00649 (PMC13316980; doi:10.1021/acs.joc.6c00649)
Supplement: Supplementary file 1 [file jo6c00649_si_001.pdf]

Supporting Information

Atypical Outcome of the Giese Reaction with Halogenated Enones

Vojtěch Kundera,[a] Judit Šponer,[b] and Jakub Švenda[a,c]*

[a] Department of Chemistry, Faculty of Science, Masaryk University, Kamenice 5, Brno 625 00, Czech Republic

[b] Institute of Biophysics, Czech Academy of Sciences, Brno 612 65, Czech Republic

[c] International Clinical Research Center, St. Anne's University Hospital, Brno 656 91, Czech Republic

*Email: svenda@chemi.muni.cz

Table of Contents

General Experimental Procedures S2

Materials S2

Instrumentation S2

Experimental Procedures S3

- Halogenated Enones (**10**, **12**, **14**, **32**) and Their Photoredox Reactions S3
- Synthetic Sequence to Deuterated Fluorinated Enone **10**-*d* S15
- Expanded Table for Scheme 6 with Fragment Coupling Conditions S21
- Competition Experiment S22
- Radical Trapping Experiment S23
- Classic Giese Reaction with the α'-Hydroxy Enone **33** S25

X-Ray Crystallography S26

Computational Data S29

References S39

NMR Spectra S40

General Experimental Procedures

All reactions were performed in round-bottom flasks fitted with rubber septa under a positive argon pressure, unless noted otherwise. All reactions were monitored by thin-layer chromatography (TLC) using aluminum plates pre-coated with silica gel (silica gel 60 F254, Merck) impregnated with a fluorescent indicator. TLC plates were visualized by exposure to UV light (λ = 254 nm) and/or by submersion in aqueous potassium permanganate ($KMnO_4$) solution followed by brief heating. All solutions were concentrated by rotary evaporation at 40 °C, unless noted otherwise. Flash-column chromatography (FCC) was performed using silica gel (60 Å, 230–400 mesh, Sigma-Aldrich). Structural assignments were made with additional information from COSY, HSQC, NOESY and HMBC experiments. For the reactions requiring blue LED irradiation, 40W Kessil® A160WE Tuna Blue lamp was set on the blue light setting (λ = 456 nm) on maximum brightness.

Materials

All reagents purchased from commercial suppliers (Sigma-Aldrich, Fluorochem, BLD Pharm, TCI Chemicals) were used without further purification. All solvents were used as received. Sodium borodeuteride ($NaBD_4$, 98 atom% D) was purchased from Sigma-Aldrich.

Instrumentation

Proton nuclear magnetic resonance (^{1}H NMR) spectra were recorded using Bruker Avance 500 (500 MHz) NMR spectrometer at 30 °C. Proton chemical shifts are expressed in parts per million (ppm, δ scale) and are referenced to residual protium in the NMR solvents ($C\mathit{H}Cl_3$: δ = 7.26 ppm, $C\mathit{H}D_2SOCD_3$: δ = 2.50 ppm (quint)). Data are represented as follows: chemical shift, multiplicity (s = singlet, d = doublet, t = triplet, q = quartet, quint = quintet, m = multiplet and/or multiple resonances, app = apparent, br = broad), coupling constants (J) in Hertz, integration. Carbon nuclear magnetic resonance (^{13}C{^{1}H} NMR) spectra were recorded using Bruker Avance 500 (126 MHz) NMR spectrometer at 30 °C. Carbon chemical shifts are expressed in parts per million (ppm, δ scale) and are referenced to the carbon resonance of the NMR solvent ($\mathit{C}DCl_3$: δ = 77.2 ppm (t)). Fluorine nuclear magnetic resonance (^{19}F NMR) spectra were recorded using Bruker Avance 500 (471 MHz) NMR spectrometer at 30 °C. Fluorine chemical shifts are expressed in parts per million (ppm, δ scale) without reference. Fourier transform infrared (FTIR) spectra were obtained using ALPHA Bruker FTIR spectrometer equipped with a diamond ATR adaptor. High-resolution mass spectra were obtained on Agilent 6224 Accurate-Mass TOF LC-MS with dual electrospray/chemical ionization mode. Melting points were obtained using the Stuart SMP40 Melting Point Apparatus.

Experimental Procedures

Halogenated Enones (10, 12, 14, 32) and Their Photoredox Reactions

Synthesis of Fluorinated Enones 10 and 12

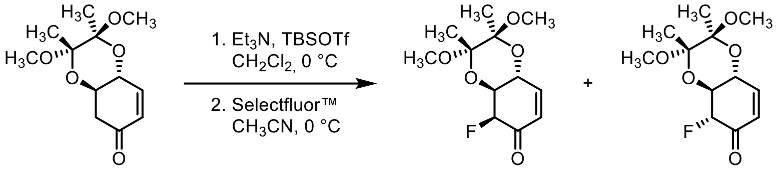

1 **10**, 64% **12**, 7%

Note: Generated silyl enol ether intermediate tends to hydrolyze readily and should be forwarded to the fluorination step as quickly as possible to achieve reproducible yields.

Triethylamine (4.60 mL, 33.0 mmol, 4.0 equiv) was added dropwise over ca. 2 min to a stirred solution of enone **1** (2.00 g, 8.26 mmol, 1 equiv) in dichloromethane (40.0 mL) at 23 °C. The reaction flask was placed in a 0 °C cooling bath (ice–sodium chloride–water). Then, *tert*-butyldimethylsilyl trifluoromethanesulfonate (2.84 mL, 12.4 mmol, 1.5 equiv) was added to the solution dropwise over ca. 2 min at 0 °C. The reaction mixture was stirred at 0 °C for 1 h. The 0 °C cooling bath was removed, and the reaction mixture was stirred at 23 °C for an additional 30 min until TLC analysis (20% ethyl acetate in cyclohexane, $KMnO_4$) showed full consumption of the starting material **1**. The reaction mixture was concentrated in vacuo (rotavap) and then under high vacuum for ca. 15 min. The reaction flask containing the crude TBS enol ether (not shown) was purged with argon, and anhydrous acetonitrile (60.0 mL) was added. The resulting solution was cooled in a 0 °C cooling bath (ice–sodium chloride–water), and the mixture was stirred at this temperature for 10 min. Selectfluor™ (4.39 g, 12.4 mmol, 1.5 equiv) was added in one portion at 0 °C, and the reaction mixture was stirred for 30 min at this temperature. TLC analysis (20% ethyl acetate in cyclohexane, $KMnO_4$) showed full consumption of the TBS enol ether, with minor amounts of the hydrolyzed silyl enol ether detected. The reaction flask was removed from the 0 °C cooling bath, and the reaction mixture was concentrated in vacuo. The residue was diluted with dichloromethane (80 mL) and transferred to a separatory funnel containing 80 mL of water. The organic layer was separated, and the aqueous layer was extracted with dichloromethane (3 × 60 mL). Combined organic layers were dried over anhydrous $MgSO_4$, and the dried solution was filtered through cotton and concentrated in vacuo. The residue was purified by flash column chromatography (20% ethyl acetate in cyclohexane) to afford separately pseudoaxial fluorinated enone **10** as a white solid (1.37 g, 64%) and pseudoequatorial fluorinated enone **12** as a light-yellow solid (151 mg, 7%). ^1H NMR spectral data of the pseudoaxial fluorinated enone **10** matched those published in the literature.[1] Single crystals suitable for X-ray crystallographic analysis were obtained by slow evaporation of a solution of **10** in ethyl acetate at 23 °C.

Pseudoaxial fluorinated enone **10**: **TLC** (20% ethyl acetate in cyclohexane, $KMnO_4$): R_f = 0.43. **mp** 175.5–177.7 °C. **^{1}H NMR** (500 MHz, $CDCl_3$) δ: 6.98 (dd, *J* = 10.3, 1.6 Hz, 1H), 6.08 (ddd, *J* = 10.2, 2.5, 1.2 Hz, 1H), 4.87 (app dt, *J* = 8.8, 2.3 Hz, 1H), 4.81 (app dt, *J* = 52.1, 1.5 Hz, 1H), 4.00 (ddd, *J* = 34.8, 8.5, 2.1 Hz, 1H), 3.33 (s, 3H), 3.28 (s, 3H), 1.39 (s, 3H), 1.36 (s, 3H). **^{13}C{^{1}H} NMR** (126 MHz, $CDCl_3$) δ: 190.4 (d, *J* = 16.9 Hz), 150.1, 127.8, 101.1, 100.7, 89.8 (d, *J* = 182.6 Hz), 71.2 (d, *J* = 18.0 Hz), 64.1 (d, *J* = 7.4 Hz), 48.4, 48.4, 17.8, 17.6. **^{19}F NMR** (471 MHz, $CDCl_3$) δ: −206.5 (dd, *J* = 52.0, 34.7 Hz).

Pseudoequatorial fluorinated enone **12**: **TLC** (20% ethyl acetate in cyclohexane, $KMnO_4$): R_f = 0.26. **^{1}H NMR** (500 MHz, $CDCl_3$) δ: 6.93 (dd, *J* = 10.2, 1.8 Hz, 1H), 6.12 (ddd, *J* = 10.2, 4.0, 2.8 Hz, 1H), 4.88 (dd, *J* = 50.1, 11.6 Hz, 1H), 4.62 (dddd, *J* = 8.9, 2.9, 1.8, 1.1 Hz, 1H), 4.16 (app td, *J* = 11.3, 8.8 Hz, 1H), 3.32 (s, 3H), 3.29 (s, 3H), 1.37 (s, 3H), 1.37 (s, 3H). **^{13}C{^{1}H} NMR** (126 MHz, $CDCl_3$) δ: 192.3 (d, *J* = 14.5 Hz), 149.0 (d, *J* = 1.5 Hz), 128.3, 101.0, 99.9, 90.5 (d, *J* = 196.2 Hz), 72.3 (d, *J* = 17.4 Hz), 67.1 (d, *J* = 10.1 Hz), 48.5, 48.3, 17.7, 17.6. **^{19}F NMR** (471 MHz, $CDCl_3$; contains <5% of unidentified fluorinated impurity) δ: −205.0 (dddd, *J* = 50.2, 11.1, 3.9, 1.1 Hz). **FTIR** (neat), cm^{-1}: 2994, 2950, 2837, 1701, 1377, 1134, 1112, 1076, 1051, 1031, 995, 931, 885, 846, 797, 731, 551. **HRMS** (APCI+) *m/z*: $[M - CH_3OH + H]^+$ Calcd for $C_{11}H_{14}FO_4$ 229.0871; Found 229.0870.

Synthesis of Gem-Difluorinated Enone 14

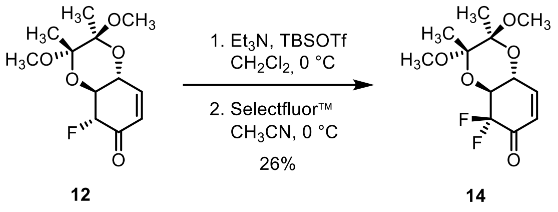

Triethylamine (0.11 mL, 0.77 mmol, 4.0 equiv) was added dropwise over ca. 1 min to a stirred solution of pseudoequatorial fluorinated enone **12** (50 mg, 0.192 mmol, 1 equiv) in dichloromethane (2.0 mL) at 23 °C. The reaction flask was placed in a 0 °C cooling bath (ice–sodium chloride–water). Then, *tert*-butyldimethylsilyl trifluoromethanesulfonate (66 µL, 0.29 mmol, 1.5 equiv) was added dropwise over ca. 1 min at 0 °C. The reaction mixture was stirred at 0 °C for 2 h 30 min. Additional portions of triethylamine (55 µL, 0.38 mmol, 2.0 equiv) and *tert*-butyldimethylsilyl trifluoromethanesulfonate (33 µL, 0.19 mmol, 1.0 equiv) were added at 0 °C. The reaction mixture was stirred for an additional 2 h until TLC analysis (20% ethyl acetate in cyclohexane, $KMnO_4$) showed near full consumption of the starting material **12**. The reaction flask was removed from the 0 °C cooling bath, and the reaction mixture was concentrated in vacuo (rotavap) and then under high vacuum for ca. 10 min. The reaction flask containing the crude TBS enol ether (not shown) was purged with argon, and anhydrous acetonitrile (3.0 mL) was added. The resulting solution was cooled in a 0 °C cooling bath (ice–sodium chloride–water), and the mixture was stirred at this temperature for 10 min. Selectfluor™ (102 mg, 0.288 mmol, 1.5 equiv) was added in one portion at 0 °C, and the reaction mixture was stirred for 45 min at this temperature. TLC analysis (20% ethyl acetate in cyclohexane, $KMnO_4$) showed full consumption of the TBS enol ether, with minor hydrolysis of the silyl enol ether detected. The reaction flask was removed from the 0 °C cooling bath, quenched with a saturated aqueous solution of NH_4Cl (5.0 mL), and transferred to a separatory funnel containing 10 mL of water and 20 mL of dichloromethane. The organic layer was separated, and the aqueous layer was extracted with dichloromethane (2 × 20 mL). Combined organic layers were dried over anhydrous $MgSO_4$, and the dried solution was filtered through cotton and concentrated in vacuo. The residue was purified by flash-column chromatography (20% ethyl acetate in cyclohexane) to afford gem-difluorinated enone **14** as a white solid (14 mg, 26%).

<u>Gem-difluorinated enone **14**:</u> **TLC** (20% ethyl acetate in cyclohexane, $KMnO_4$): R_f = 0.35. **^{1}H NMR** (500 MHz, $CDCl_3$) δ: 7.02 (dd, J = 10.5, 1.6 Hz, 1H), 6.17 (ddd, J = 10.4, 4.1, 2.8 Hz, 1H), 4.78 (app dt, J = 8.5, 2.1 Hz, 1H), 4.16 (ddd, J = 25.3, 8.4, 4.4 Hz, 1H), 3.33 (s, 3H), 3.30 (s, 3H), 1.41 (s, 3H), 1.36 (s, 3H). **^{13}C{^{1}H} NMR** (126 MHz, $CDCl_3$) δ: 185.2 (app t, J = 24.6 Hz), 150.4, 126.8 (d, J = 2.6 Hz), 111.0 (dd, J = 259.9, 250.6 Hz), 101.1, 100.6, 71.4 (app t, J = 18.4 Hz), 65.7 (dd, J = 7.4, 2.9 Hz), 48.5, 48.5, 17.7, 17.5. **^{19}F NMR** (471 MHz, $CDCl_3$) δ: −124.0 (dd, J = 272.5, 25.2 Hz), −127.4 (app dt, J = 272.9, 4.7 Hz). **FTIR** (neat), cm^{-1}: 2998, 2952, 2925, 2852, 1714, 1461, 1378, 1216, 1143, 1111, 1100, 1057, 1035, 1018, 973, 923,

894, 821. **HRMS** (APCI+) *m/z*: [M − CH_3OH + H]$^+$ Calcd for $C_{11}H_{13}F_2O_4$ 247.0776; Found 247.0779.

Synthesis of Pseudoaxial Chlorinated Enone 32

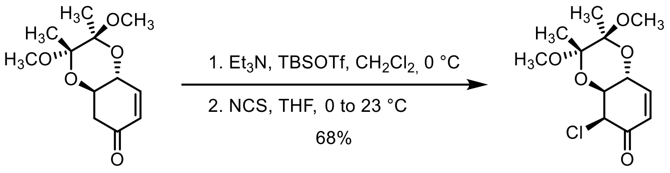

1 **32**

Triethylamine (0.69 mL, 4.95 mmol, 4.0 equiv) was added dropwise over ca. 2 min to a stirred solution of enone **1** (300 mg, 1.24 mmol, 1 equiv) in dichloromethane (6.0 mL) at 23 °C. The reaction flask was placed in a 0 °C cooling bath (ice–sodium chloride–water). Then, *tert*-butyldimethylsilyl trifluoromethanesulfonate (0.43 mL, 1.86 mmol, 1.5 equiv) was added to the solution dropwise over ca. 2 min at 0 °C. The reaction mixture was stirred at 0 °C for 1 h. The 0 °C cooling bath was removed, and the reaction mixture was stirred at 23 °C for an additional 30 min until TLC analysis (20% ethyl acetate in cyclohexane, $KMnO_4$) showed full consumption of the starting material **1**. The reaction mixture was concentrated in vacuo (rotavap) and then under high vacuum for ca. 15 min. The reaction flask containing the crude TBS enol ether (not shown) was purged with argon, and anhydrous tetrahydrofuran (6.0 mL) was added. The resulting solution was cooled in a 0 °C cooling bath (ice–sodium chloride–water), and the mixture was stirred at this temperature for 10 min. *N*-Chlorosuccinimide (248 mg, 1.86 mmol, 1.5 equiv) was added in one portion at 0 °C, and the reaction mixture was stirred for 30 min at this temperature. TLC analysis (20% ethyl acetate in cyclohexane, $KMnO_4$) showed full consumption of the TBS enol ether, with minor amounts of hydrolyzed silyl enol ether detected. The reaction mixture was quenched with a saturated aqueous solution of NH_4Cl (10 mL) and transferred to a separatory funnel containing 30 mL of water and 40 mL of dichloromethane. The organic layer was separated, and the aqueous layer was extracted with dichloromethane (2 × 30 mL). Combined organic layers were dried over anhydrous $MgSO_4$, and the dried solution was filtered through cotton and concentrated in vacuo. The residue was purified by flash-column chromatography (20% ethyl acetate in cyclohexane) to afford pseudoaxial chlorinated enone **32** as a white solid (232 mg, 68%).

<u>Pseudoaxial chlorinated enone **32**:</u> **TLC** (20% ethyl acetate in cyclohexane, $KMnO_4$): R_f = 0.49. **^{1}H NMR** (500 MHz, $CDCl_3$) δ: 6.91 (dd, *J* = 10.3, 1.8 Hz, 1H), 6.05 (ddd, *J* = 10.4, 2.7, 1.1 Hz, 1H), 4.84 (app dt, *J* = 8.4, 2.2 Hz, 1H), 4.35 (dd, *J* = 3.4, 0.9 Hz, 1H), 4.14 (dd, *J* = 8.3, 3.4 Hz, 1H), 3.34 (s, 3H), 3.27 (s, 3H), 1.38 (s, 3H), 1.36 (s, 3H). **^{13}C{^{1}H} NMR** (126 MHz, $CDCl_3$) δ: 190.3, 149.0, 127.4, 101.0, 100.7, 70.1, 65.0, 58.6, 48.4 (2C), 17.8, 17.6. **FTIR** (neat), cm^{-1}: 2994, 2951, 2882, 2835, 1716, 1457, 1373, 1244, 1204, 1132, 1107, 1086, 1052, 1039, 928, 907, 885, 859, 704, 645, 569, 471, 453, 419. **HRMS** (APCI+) *m/z*: $[M - CH_3OH + H]^+$ Calcd for $C_{11}H_{14}ClO_4$ 245.0575; Found 245.0577.

Synthesis of α'-Hydroxy Enone 33

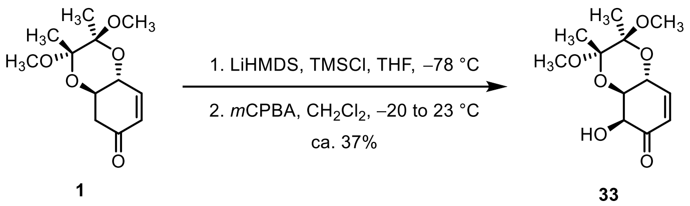

A solution of lithium hexamethyldisilazide (1 M in THF, 1.86 mL, 1.86 mmol, 1.5 equiv) was added dropwise over 1 min to a stirred solution of enone **1** (300 mg, 1.238 mmol, 1 equiv) in anhydrous tetrahydrofuran (8.0 mL) at −78 °C (dry ice–acetone cooling bath). The resulting mixture was stirred for 30 min at this temperature. Trimethylsilyl chloride (0.79 mL, 6.19 mmol, 5.0 equiv) was added dropwise over 1 min at −78 °C. The reaction mixture was stirred for 20 min at −78 °C, then brought up to 0 °C (ice–sodium chloride–water cooling bath) and stirred at this temperature for 20 min. The cooling bath was then removed, and the mixture was stirred for 20 min at 23 °C. The reaction mixture was concentrated in vacuo (rotavap) and then under high vacuum for ca. 10 min. The reaction flask containing the crude TMS enol ether (not shown) was purged with argon, and dichloromethane (4.0 mL) was added. The resulting solution was cooled in a −20 °C cooling bath (ice–sodium chloride), and the mixture was stirred at this temperature for 10 min. A solution of freshly purified[2] *m*CPBA (416 mg, 1.86 mmol, 1.5 equiv) in 4.0 mL dichloromethane was added dropwise over 1 min at −20 °C, and the reaction mixture was slowly brought up to 23 °C, while stirring for 2 h. TLC analysis (30% ethyl acetate in cyclohexane, $KMnO_4$) showed full consumption of the TMS enol ether, with minor amounts of hydrolyzed silyl enol ether detected. The reaction mixture was concentrated in vacuo (rotavap) and redissolved in methanol (5.0 mL). A saturated aqueous solution of NH_4Cl (5.0 mL) was added, and the mixture was vigorously stirred for 30 min. The mixture was then transferred to a separatory funnel containing 40 mL of water and 40 mL of dichloromethane. The organic layer was separated, and the aqueous layer was extracted with dichloromethane (3 × 40 mL). Combined organic layers were dried over anhydrous $MgSO_4$, and the dried solution was filtered through cotton and concentrated in vacuo. The residue was purified by flash-column chromatography (40% ethyl acetate in cyclohexane) to afford α'-hydroxy enone **33** as an off-white solid (127 mg, ca. 37%, contains traces of *m*CPBA).

α'-Hydroxy enone **33**: **TLC** (40% ethyl acetate in cyclohexane, $KMnO_4$): R_f = 0.51. **^{1}H NMR** (500 MHz, $CDCl_3$) δ: 6.94 (dd, J = 10.2, 1.7 Hz, 1H), 6.02 (ddd, J = 10.2, 2.7, 1.2 Hz, 1H), 4.90 (app dt, J = 8.7, 2.2 Hz, 1H), 4.21 (dd, J = 3.1, 0.9 Hz, 1H), 3.95 (dd, J = 8.8, 3.1 Hz, 1H), 3.32 (s, 3H), 3.27 (s, 3H), 2.49 (br s, 1H), 1.37 (s, 3H), 1.36 (s, 3H). **^{13}C{^{1}H} NMR** (126 MHz, $CDCl_3$) δ: 194.7, 149.6, 127.9, 100.9, 100.5, 72.6, 71.6, 64.2, 48.3, 48.3, 17.9, 17.7. **FTIR** (neat), cm^{-1}: 3456, 2994, 2951, 2917, 2835, 1717, 1686, 1457, 1376, 1213, 1178, 1142, 1131, 1113, 1089, 1050, 1036, 930, 885, 863, 420. **HRMS** (APCI+) *m/z*: $[M - CH_3OH + H]^+$ Calcd for $C_{11}H_{15}O_5$ 227.0914; Found 227.0917.

Synthesis of α’-Acetoxy Enone 34

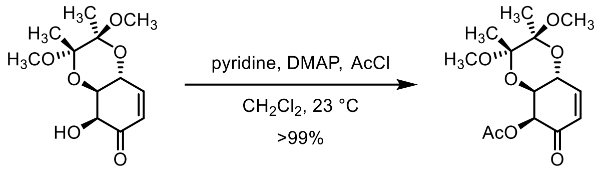

Acetyl chloride (55 µL, 0.77 mmol, 2.0 equiv) was added dropwise over 1 min to a stirred solution of α’-hydroxy enone **33** (100 mg, 0.387 mmol, 1 equiv), pyridine (94 µL, 1.16 mmol, 3.0 equiv), and 4-dimethylaminopyridine (5 mg, 0.039 mmol, 0.1 equiv) in anhydrous dichlorometane (4.0 mL) at 23 °C. The resulting mixture was stirred for 1 h at 23 °C. TLC analysis (30% ethyl acetate in cyclohexane, $KMnO_4$) showed full consumption of starting material **33**. Then, 5 mL of saturated aqueous solution of $NaHCO_3$ was added, and the reaction mixture was transferred to a separatory funnel containing 30 mL of water and 30 mL of dichloromethane. After layer separation, the organic layer was collected, and the aqueous layer was extracted with dichloromethane (2 × 30 mL). Combined organic layers were dried over anhydrous $MgSO_4$, and the dried solution was filtered through cotton and concentrated in vacuo. The residue was purified by flash-column chromatography (30% ethyl acetate in cyclohexane) to afford α’-acetoxy enone **34** as a light-yellow oil (120 mg, >99%).

α’-Acetoxy enone **34**: **TLC** (30% ethyl acetate in cyclohexane, $KMnO_4$): R_f = 0.47. **^{1}H NMR** (500 MHz, $CDCl_3$) *δ*: 6.93 (dd, *J* = 10.4, 1.7 Hz, 1H), 6.03 (ddd, *J* = 10.4, 2.7, 1.2 Hz, 1H), 5.53 (dd, *J* = 3.4, 1.1 Hz, 1H), 4.80 (app dt, *J* = 8.5, 2.2 Hz, 1H), 4.03 (dd, *J* = 8.5, 3.4 Hz, 1H), 3.31 (s, 3H), 3.25 (s, 3H), 2.11 (s, 3H), 1.34 (s, 3H), 1.29 (s, 3H). **^{13}C{^{1}H} NMR** (126 MHz, $CDCl_3$) *δ*: 191.8, 169.3, 149.7, 128.3, 101.0, 100.3, 71.8, 70.2, 64.4, 48.3, 48.3, 20.9, 17.8, 17.6. **FTIR** (neat), cm^{-1}: 2995, 2951, 2892, 2836, 1756, 1690, 1457, 1373, 1262, 1216, 1187, 1134, 1111, 1085, 1050, 1035, 1025, 955, 930, 888, 860, 774, 619, 571, 519, 495, 423. **HRMS** (APCI+) *m/z*: $[M - CH_3OH + H]^+$ Calcd for $C_{13}H_{17}O_6$ 269.1020; Found 269.1023.

Representative Procedure Leading to Defluorinated Coupled Product 11

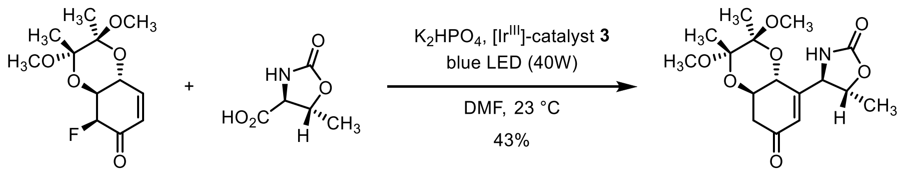

Pseudoaxial fluorinated enone **10** (100 mg, 0.384 mmol, 2.0 equiv), oxazolidinone **2** (28 mg, 0.192 mmol, 1 equiv), dipotassium phosphate (34 mg, 0.192 mmol, 1.0 equiv), and [Ir^{III}]-catalyst **3** (2.2 mg, 0.0019 mmol, 0.01 equiv) were added to a reaction flask. The flask was evacuated and backfilled with argon (3 cycles). Anhydrous *N*,*N*-dimethylformamide (3.0 mL) was added at 23 °C, and the reaction mixture was degassed by bubbling argon through the solution for 20 min (outlet needle) with stirring at 23 °C. A double-layered balloon filled with argon was attached, and the reaction mixture was stirred for 18 h at 23 °C (a cooling fan was set up to maintain the ambient temperature of the reaction mixture) while being irradiated by a blue LED lamp (40W Kessil® A160WE Tuna Blue lamp) placed about 4 cm away from the reaction flask. TLC analysis (20% acetone in dichloromethane, $KMnO_4$) showed the formation of a new UV-active spot. The blue LED was turned off, and the reaction mixture was transferred to a separatory funnel containing 30 mL of ethyl acetate and 30 mL of ice-cold water. The mixture was extracted, the organic layer was separated, and the aqueous layer was extracted with ethyl acetate (2 × 30 mL). Combined organic layers were washed with ice-cold water (4 × 30 mL) and brine (1 × 30 mL). The washed organic layer was dried over anhydrous $MgSO_4$. The dried solution was filtered through cotton and concentrated in vacuo. The residue was purified by flash-column chromatography (20% acetone in dichloromethane) to afford the coupled product **11** as a white foam (28 mg, 43%).

Defluorinated coupled product **11**: **TLC** (20% acetone in dichloromethane, $KMnO_4$): R_f = 0.40. **^{1}H NMR** (500 MHz, $CDCl_3$) δ: 6.09 (app dt, *J* = 2.5, 1.2 Hz, 1H), 5.97 (br s, 1H), 4.58 (qd, *J* = 6.2, 3.6 Hz, 1H), 4.54 (ddd, *J* = 9.2, 2.6, 1.1 Hz, 1H), 4.21 (dq, *J* = 3.7, 1.2 Hz, 1H), 4.05 (ddd, *J* = 13.4, 9.1, 5.0 Hz, 1H), 3.30 (s, 3H), 3.24 (s, 3H), 2.75 (ddd, *J* = 16.6, 5.0, 1.1 Hz, 1H), 2.49 (dd, *J* = 16.6, 13.3 Hz, 1H), 1.54 (d, *J* = 6.2 Hz, 3H), 1.34 (s, 3H), 1.32 (s, 3H). **^{13}C{^{1}H} NMR** (126 MHz, $CDCl_3$) δ: 195.1, 158.8, 158.8, 125.2, 101.1, 99.8, 79.1, 70.4, 68.1, 58.7, 48.5, 48.3, 42.2, 20.9, 17.8, 17.6. **FTIR** (neat), cm^{-1}: 2924, 2853, 1749, 1677, 1454, 1379, 1258, 1223, 1131, 1111, 1083, 1051, 1033, 933, 876, 735. **HRMS** (APCI−) *m/z*: [M + Cl]$^-$ Calcd for $C_{16}H_{23}NO_7Cl$ 376.1169; Found 376.1165.

Preparative Procedure Used for Isolation of the Fluorinated Coupled Product 24

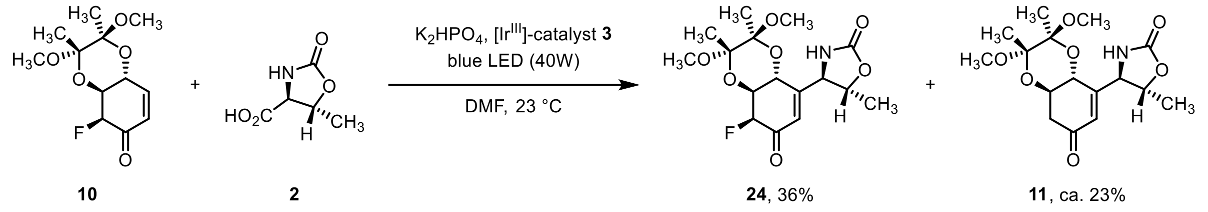

Pseudoaxial fluorinated enone **10** (150 mg, 0.576 mmol, 4.0 equiv), oxazolidinone **2** (21 mg, 0.144 mmol, 1 equiv), dipotassium phosphate (25 mg, 0.144 mmol, 1.0 equiv), and [Ir^{III}]-catalyst **3** (1.6 mg, 0.0014 mmol, 0.01 equiv) were added to a reaction flask. The flask was evacuated and backfilled with argon (3 cycles). Anhydrous *N,N*-dimethylformamide (3.0 mL) was added at 23 °C, and the reaction mixture was degassed by bubbling argon through the solution for 20 min (outlet needle) with stirring at 23 °C. A double-layered balloon filled with argon was attached, and the reaction mixture was stirred for 1 h at 23 °C (a cooling fan was set up to maintain the ambient temperature of the reaction mixture) while being irradiated by a blue LED lamp (40W Kessil® A160WE Tuna Blue lamp) placed about 4 cm away from the reaction flask. TLC analysis (20% acetone in dichloromethane, $KMnO_4$) showed the formation of 2 new UV-active spots. The blue LED was turned off, and the reaction mixture was transferred to a separatory funnel containing 20 mL of ethyl acetate and 20 mL of ice-cold water. The mixture was extracted, the organic layer was separated, and the aqueous layer was extracted with ethyl acetate (2 × 20 mL). Combined organic layers were washed with ice-cold water (4 × 20 mL) and brine (1 × 20 mL). The washed organic layer was dried over anhydrous $MgSO_4$. The dried solution was filtered through cotton and concentrated in vacuo. The residue was purified by flash-column chromatography (20% acetone in dichloromethane) to afford fluorinated coupled product **24** as an off-white solid (19 mg, 36%) and defluorinated coupled product **11** as a light-yellow solid (12 mg, ca. 23%, containing trace impurities). Single crystals suitable for X-ray crystallographic analysis were obtained by slow evaporation of a solution of **24** in $CDCl_3$ at 23 °C.

Fluorinated coupled product **24**: **TLC** (20% acetone in dichloromethane, $KMnO_4$): R_f = 0.67. **mp** 250.7–261.3 °C (decomposition).**^{1}H NMR** (500 MHz, $CDCl_3$) δ: 6.19 (app dt, J = 2.3, 1.1 Hz, 1H), 5.65 (br s, 1H), 4.91 (dd, J = 8.5, 1.9 Hz, 1H), 4.80 (app dt, J = 52.1, 1.6 Hz, 1H), 4.58 (qd, J = 6.2, 3.5 Hz, 1H), 4.23 (dq, J = 3.4, 1.1 Hz, 1H), 4.02 (ddd, J = 34.0, 8.5, 2.1 Hz, 1H), 3.32 (s, 3H), 3.26 (s, 3H), 1.56 (d, J = 6.3 Hz, 3H), 1.38 (s, 3H), 1.35 (s, 3H). **^{13}C{^{1}H} NMR** (126 MHz, $CDCl_3$) δ: 189.0 (d, J = 17.0 Hz), 160.5, 158.4, 123.0, 101.2, 100.7, 89.7 (d, J = 182.6 Hz), 78.9, 70.9 (d, J = 17.9 Hz), 65.3 (d, J = 7.3 Hz), 58.9, 48.6, 48.5, 20.9, 17.6. **^{19}F NMR** (471 MHz, $CDCl_3$) δ: −205.8 (dd, J = 52.0, 34.0 Hz). **FTIR** (neat), cm^{-1}: 2993, 2950, 2928, 2836, 1740, 1689, 1379, 1269, 1216, 1131, 1110, 1033, 934, 886, 871, 849, 735. **HRMS** (APCI−) *m/z*: $[M + Cl]^-$ Calcd for $C_{16}H_{22}FNO_7Cl$ 394.1074; Found 394.1078.

Representative Coupling Procedure with Pseudoequatorial Enone 12

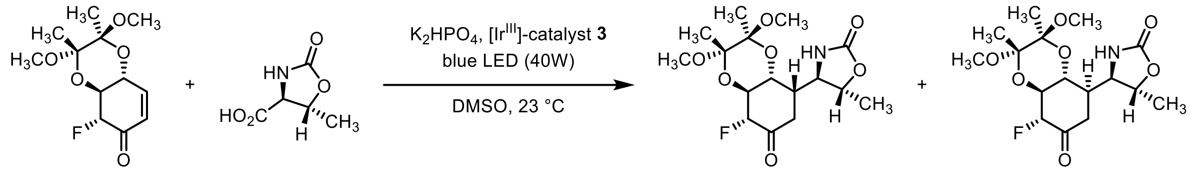

Pseudoequatorial fluorinated enone **12** (69 mg, 0.265 mmol, 1 equiv), oxazolidinone **2** (46 mg, 0.318 mmol, 1.2 equiv), dipotassium phosphate (55 mg, 0.318 mmol, 1.2 equiv), and [Ir^{III}]-catalyst **3** (3.0 mg, 0.0027 mmol, 0.01 equiv) were added to the reaction flask. The flask was evacuated and backfilled with argon (3 cycles). Anhydrous dimethyl sulfoxide (3.0 mL) was added at 23 °C, and the reaction mixture was degassed by bubbling argon through the solution for 20 min (outlet needle) with stirring at 23 °C. A double-layered balloon filled with argon was attached, and the reaction mixture was stirred for 1 h 45 min at 23 °C (a cooling fan was set up to maintain the ambient temperature of the reaction mixture) while being irradiated by a blue LED lamp (40W Kessil® A160WE Tuna Blue lamp) placed about 4 cm away from the reaction flask. TLC analysis (20% acetone in dichloromethane, $KMnO_4$) showed full consumption of starting material **12**. The blue LED was turned off, and the reaction mixture was transferred to a separatory funnel containing 30 mL of ethyl acetate and 30 mL of ice-cold water. The mixture was extracted, the organic layer was separated, and the aqueous layer was extracted with ethyl acetate (2 × 30 mL). Combined organic layers were washed with ice-cold water (4 × 30 mL) and brine (1 × 30 mL). The washed organic layer was dried over anhydrous $MgSO_4$. The dried solution was filtered through cotton and concentrated in vacuo. The residue was purified by flash-column chromatography (20% acetone in dichloromethane) to afford 3 different fractions. The first two fractions contained impure products **25** and **26**, and the third fraction contained the defluorinated coupled product **11** (8 mg, ca. 8%; contains impurities and traces of **26** – overlap with the second fraction). The first fraction containing **25** was repurified by a second flash-column chromatography (70% ethyl acetate in cyclohexane) to afford pseudoequatorial fluorinated Giese product **25** as a colorless oil (16 mg, ca. 16%; contains approx. 10% of inseparable defluorinated Giese adduct **5**). The second fraction containing **26** was repurified by a second flash-column chromatography (80% ethyl acetate in cyclohexane) to afford the epimeric pseudoequatorial fluorinated Giese product **26** as a colorless oil (12 mg, ca. 12%; contains only minor impurities).

Pseudoequatorial fluorinated Giese product **25**: **TLC** (20% acetone in dichloromethane, $KMnO_4$): R_f = 0.68. **^{1}H NMR** (500 MHz, $CDCl_3$) δ: 5.85 (br s, 1H), 4.94 (dd, J = 49.9, 10.2 Hz, 1H), 4.35–4.26 (m, 2H), 4.10 (app dt, J = 14.6, 10.4 Hz, 1H), 3.37 (dd, J = 11.0, 6.3 Hz, 1H), 3.30 (s, 3H), 3.27 (s, 3H), 2.57 (dd, J = 15.1, 6.0 Hz, 1H), 2.45 (ddd, J = 15.1, 5.8, 2.4 Hz, 1H), 2.37 (ddd, J = 10.6, 6.4, 2.2 Hz, 1H), 1.48 (d, J = 6.3 Hz, 3H), 1.35 (s, 3H), 1.34 (s, 3H). **^{13}C{^{1}H} NMR** (126 MHz, $CDCl_3$) δ: 199.7 (d, J = 14.7 Hz), 157.6, 100.5, 99.4, 91.7 (d, J =

199.5 Hz), 77.5, 69.4 (d, *J* = 8.3 Hz), 69.2 (d, *J* = 17.9 Hz), 58.7, 48.7, 48.5, 41.0, 38.0, 21.0, 17.8, 17.4. **^{19}F NMR** (471 MHz, $CDCl_3$) *δ*: −202.3 (ddd, *J* = 50.0, 14.5, 5.7 Hz). **FTIR** (neat), cm^{-1}: 3452, 2993, 2950, 2923, 2838, 1739, 1458, 1399, 1379, 1218, 1134, 1112, 1072, 1045, 1012, 972, 943, 914, 895, 854, 766, 730, 591, 526, 505. **HRMS** (APCI−) *m/z*: $[M + Cl]^-$ Calcd for $C_{16}H_{24}FNO_7Cl$ 396.1231; found 396.1229.

<u>Epimeric pseudoequatorial fluorinated Giese product **26**:</u> **TLC** (20% acetone in dichloromethane, $KMnO_4$): R_f = 0.35. **^{1}H NMR** (500 MHz, $CDCl_3$) *δ*: 6.41 (br s, 1H), 4.96 (dd, *J* = 50.3, 10.0 Hz, 1H), 4.43 (qd, *J* = 6.3, 4.6 Hz, 1H), 3.92–3.87 (m, 1H), 3.88–3.79 (m, 2H), 3.30 (s, 3H), 3.26 (s, 3H), 2.55 (ddd, *J* = 14.5, 5.2, 4.3 Hz, 1H), 2.35 (app t, *J* = 14.0 Hz, 1H), 1.91 (app ddq, *J* = 13.7, 7.1, 3.7 Hz, 1H), 1.45 (d, *J* = 6.3 Hz, 3H), 1.35 (s, 3H), 1.33 (s, 3H). **^{13}C{^{1}H} NMR** (126 MHz, $CDCl_3$) *δ*: 199.7 (d, *J* = 14.6 Hz), 159.4, 100.1, 99.8, 91.7 (d, *J* = 198.3 Hz), 77.0, 72.3 (d, *J* = 17.8 Hz), 67.5 (d, *J* = 8.8 Hz), 57.0, 48.7, 48.4, 40.2, 36.4, 20.9, 17.7, 17.6. **^{19}F NMR** (471 MHz, $CDCl_3$) *δ*: −203.8 (app ddq, *J* = 50.6, 11.6, 6.1 Hz). **FTIR** (neat), cm^{-1}: 2994, 2950, 2929, 2837, 1739, 1455, 1431, 1381, 1232, 1135, 1111, 1036, 1012, 991, 937, 910, 886, 853, 732. **HRMS** (APCI−) *m/z*: $[M + Cl]^-$ Calcd for $C_{16}H_{24}FNO_7Cl$ 396.1231; Found 396.1233.

Quantitative NMR Procedure for the Coupling with Pseudoequatorial Enone 12

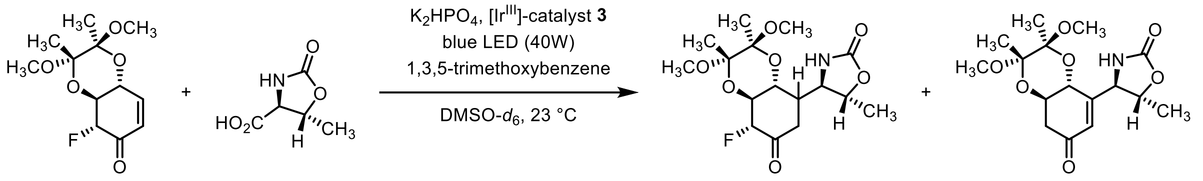

12 **2** **25/26** (1.3:1 dr) ca. 70% by NMR **11** ca. 9% by NMR

Pseudoequatorial fluorinated enone **12** (50 mg, 0.192 mmol, 1 equiv), and 1,3,5-trimethoxybenzene (11 mg, 0.067 mmol, 0.35 equiv) were dissolved in DMSO-d_6 (0.75 mL), and the solution was transferred into an NMR tube fitted with a rubber septum cap. A ^{1}H NMR spectrum of this mixture was measured as a reference point. In a separate flask, oxazolidinone **2** (34 mg, 0.231 mmol, 1.2 equiv), dipotassium phosphate (40 mg, 0.231 mmol, 1.2 equiv), and [Ir^{III}]-catalyst **3** (2.2 mg, 0.0019 mmol, 0.01 equiv) were added, and the flask was evacuated and backfilled with argon (3 cycles). The solution of **12** in DMSO-d_6 was transferred from the NMR tube to the reaction flask, and the NMR tube was washed with another portion of DMSO-d_6 (0.75 mL). The reaction mixture containing the total of 1.5 mL of DMSO-d_6 was degassed by bubbling argon through the solution for 20 min (outlet needle) with stirring at 23 °C. A double-layered balloon filled with argon was attached, and the reaction mixture was stirred for 1 h at 23 °C (a cooling fan was set up to maintain the ambient temperature of the reaction mixture) while being irradiated by a blue LED lamp (40W Kessil® A160WE Tuna Blue lamp) placed about 4 cm away from the reaction flask. TLC analysis (20% acetone in dichloromethane, $KMnO_4$) showed full consumption of starting material **12**. The blue LED was turned off, and the reaction mixture was filtered through a PTFE syringe filter (0.20 µm pore size). A ^{1}H NMR spectrum of the filtrate was measured and the yields by NMR against 1,3,5-trimethoxybenzene as an internal standard were determined for Giese products **25/26** (ca. 70% by NMR integrating against 1,3,5-trimethoxybenzene as the internal standard) and for the defluorinated coupled product **11** (ca. 9% by NMR integrating against 1,3,5-trimethoxybenzene as the internal standard).

Synthetic Sequence to Deuterated Fluorinated Enone 10-*d*

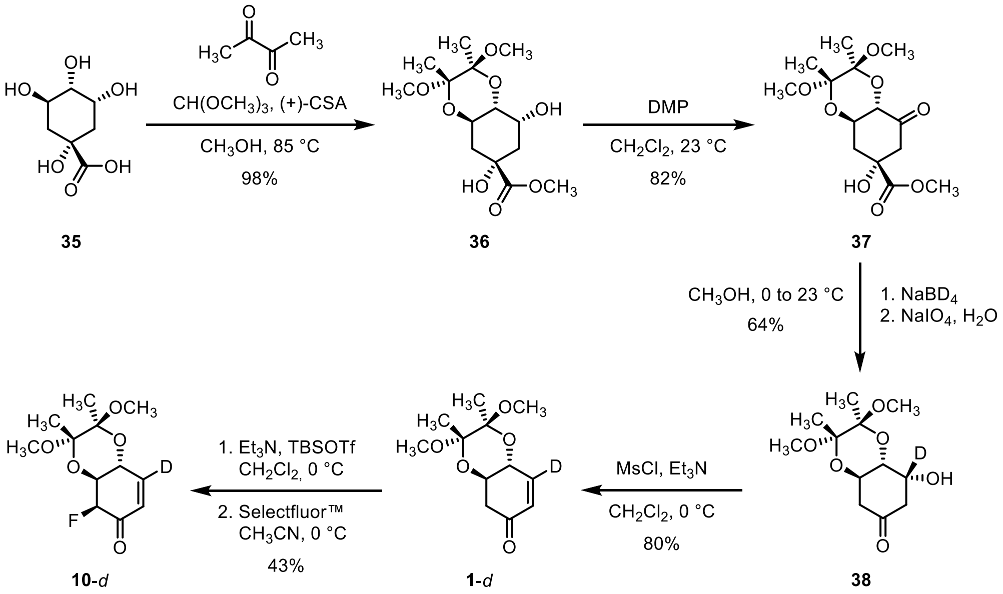

Synthesis of Diacetal (−)-Quinic Acid Methyl Ester 36

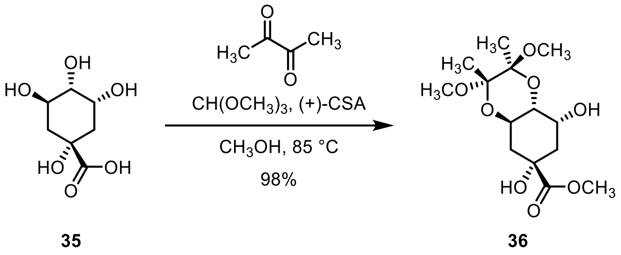

Diacetal (−)-quinic acid methyl ester **36** was prepared according to a literature procedure.[3] 2,3-Butanedione (2.54 mL, 28.62 mmol, 1.1 equiv), trimethyl orthoformate (9.39 mL, 85.86 mmol, 3.3 equiv), and (+)-camphorsulfonic acid (665 mg, 2.86 mmol, 0.11 equiv) were sequentially added to a stirred solution of (−)-quinic acid **35** (5.00 g, 26.02 mmol, 1 equiv) in methanol (50 mL) at 23 °C. The resulting mixture was placed in an 85 °C bath (DrySyn® heating block temperature) and stirred at this temperature for 20 h. TLC analysis (50% methanol in dichloromethane, $KMnO_4$) showed full consumption of starting material **35**. The reaction mixture was removed from the 85 °C bath and left to cool down to ambient temperature. The resulting mixture was concentrated in vacuo, and the residue was purified by flash-column chromatography (10% methanol in dichloromethane) to afford diacetal (−)-quinic acid methyl ester **36** as a light-yellow solid (8.12 g, 98%). ^{1}H NMR spectral data were in accordance with the literature.[3]

Diacetal (−)-quinic acid methyl ester **36**: **TLC** (5% methanol in dichloromethane, $KMnO_4$): R_f = 0.50. **^{1}H NMR** (500 MHz, $CDCl_3$) *δ*: 4.31 (ddd, *J* = 12.2, 10.1, 4.6 Hz, 1H), 4.20–4.17 (m, 2H), 3.79 (s, 3H), 3.59 (dd, *J* = 10.1, 2.9 Hz, 1H), 3.26 (s, 3H), 3.26 (s, 3H), 3.03 (dd, *J* = 3.8, 1.2 Hz, 1H), 2.18 (app dt, *J* = 14.9, 3.0 Hz, 1H), 2.10 (ddd, *J* = 12.8, 4.7, 2.9 Hz, 1H), 2.04 (ddd, *J* = 14.9, 3.1, 1.2 Hz, 1H), 1.92 (app t, *J* = 12.5 Hz, 1H), 1.34 (s, 3H), 1.29 (s, 3H).

Synthesis of Ketone 37

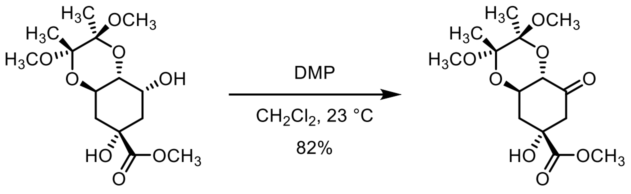

36 **37**

Dess–Martin periodinane (1.66 g, 3.90 mmol, 1.25 equiv) was added in one portion to a stirred solution of diacetal (−)-quinic acid methyl ester **36** (1.00 g, 3.12 mmol, 1 equiv) in dichloromethane (50 mL) at 23 °C. The resulting mixture was stirred for 90 min at 23 °C. TLC analysis (90% ethyl acetate in cyclohexane, $KMnO_4$) showed full consumption of starting material **36**. Saturated aqueous solutions of $NaHCO_3$ (25 mL) and $Na_2S_2O_3$ (25 mL) were added at 23 °C. The resulting biphasic mixture was vigorously stirred for an additional 20 min to reduce any remaining Dess–Martin periodinane. The mixture was diluted with 30 mL of dichloromethane and transferred to a separatory funnel containing 30 mL of water. The mixture was extracted, the organic layer was separated, and the aqueous layer was extracted with dichloromethane (2 × 50 mL). Combined organic layers were dried over anhydrous $MgSO_4$, the dried solution was filtered through cotton and concentrated in vacuo. The residue was purified by flash-column chromatography (80% ethyl acetate in cyclohexane) to afford ketone **37** as a white solid (814 mg, 82%). ^{1}H NMR spectral data were in accordance with the literature.[4]

<u>Ketone **37**:</u> **TLC** (90% ethyl acetate in cyclohexane, $KMnO_4$): R_f = 0.60. **^{1}H NMR** (500 MHz, $CDCl_3$) δ: 4.41 (dd, *J* = 10.3, 1.3 Hz, 1H), 4.27 (ddd, *J* = 12.0, 10.2, 4.4 Hz, 1H), 3.84 (s, 3H), 3.27 (s, 3H), 3.23 (s, 3H), 3.18 (br s, 1H), 2.89 (app dt, *J* = 14.2, 1.0 Hz, 1H), 2.50 (dd, *J* = 14.3, 2.9 Hz, 1H), 2.36 (ddd, *J* = 13.0, 12.0, 0.9 Hz, 1H), 2.11 (ddd, *J* = 13.1, 4.4, 2.9 Hz, 1H), 1.40 (s, 3H), 1.30 (s, 3H).

Synthesis of β-Deutero-β-hydroxy Ketone 38

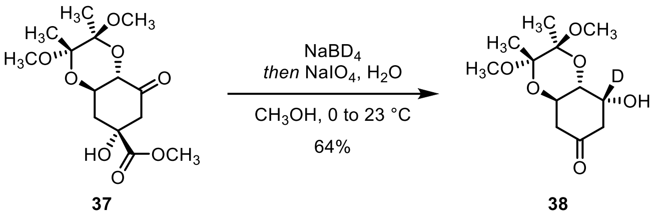

Sodium borodeuteride (329 mg, 7.854 mmol, 5.0 equiv) was slowly added to a stirred solution of ketone **37** (500 mg, 1.571 mmol, 1 equiv) in anhydrous methanol (8.0 mL) at 0 °C (ice–sodium chloride–water cooling bath). The resulting mixture was stirred for 2 h at 0 °C, then the 0 °C cooling bath was removed, and the reaction mixture was stirred for an additional 1 h at 23 °C. TLC analysis (5% methanol in ethyl acetate, $KMnO_4$) showed full consumption of the starting material **37**. The reaction mixture was placed in a 0 °C cooling bath (ice–sodium chloride–water) and quenched carefully with water (15.0 mL). Sodium periodate (1.51 g, 7.07 mmol, 4.5 equiv) was added in one portion at 0 °C. After stirring for 1 h at 0 °C, the reaction mixture was removed from the 0 °C cooling bath and stirred for 19 h at 23 °C. TLC analysis (2% methanol in ethyl acetate, $KMnO_4$) showed full consumption of vicinal diol intermediate (not shown). A saturated solution of NH_4Cl (5.0 mL) was added, and the mixture was concentrated in vacuo to remove methanol. The residue was transferred to a separatory funnel containing 40 mL of water and 50 mL of dichloromethane. The mixture was extracted, the organic layer was separated, and the aqueous layer was extracted with dichloromethane (3 × 50 mL). Combined organic layers were dried over anhydrous $MgSO_4$, and the dried solution was filtered through cotton and concentrated in vacuo. The residue was purified by flash-column chromatography (60% ethyl acetate in cyclohexane) to afford β-deutero-β-hydroxy ketone **38** as a white solid (264 mg, 64%).

β-Deutero-β-hydroxy ketone **38:** **TLC** (60% ethyl acetate in cyclohexane, $KMnO_4$): R_f = 0.49. **^{1}H NMR** (500 MHz, $CDCl_3$) δ: 4.31–4.24 (m, 1H), 3.88 (d, J = 10.1 Hz, 1H), 3.31 (s, 3H), 3.23 (s, 3H), 2.71–2.62 (m, 2H), 2.53–2.43 (m, 2H), 2.36 (d, J = 2.0 Hz, 1H), 1.34 (s, 3H), 1.31 (s, 3H). **^{13}C{^{1}H} NMR** (126 MHz, $CDCl_3$) δ: 205.5, 100.5, 99.4, 72.4, 67.5 (t, J = 23.0 Hz), 63.4, 48.3, 48.1, 46.2, 44.9, 17.9, 17.8. **FTIR** (neat), cm^{-1}: 3447, 2993, 2950, 2928, 2834, 1716, 1457, 1431, 1377, 1262, 1218, 1170, 1118, 1092, 1072, 1034, 944, 926, 905, 883, 869, 849, 816, 791, 736, 632, 577, 529, 505, 435, 413. **HRMS** (APCI+) *m/z*: [M − CH_3OH + H]$^+$ Calcd for $C_{11}H_{16}DO_5$ 230.1133; Found 230.1131.

Synthesis of β-Deutero Enone 1-*d*

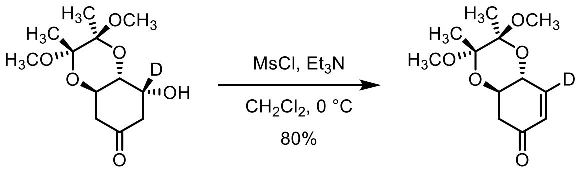

38 **1**-*d*

Triethylamine (0.42 mL, 3.03 mmol, 3.0 equiv) was added in one portion to a stirred solution of β-deutero-β-hydroxy ketone **38** (264 mg, 1.01 mmol, 1 equiv) in dichloromethane (5.0 mL) at 23 °C. The reaction mixture was placed in a 0 °C cooling bath (ice–sodium chloride–water) and stirred at this temperature for ca. 5 min. Methanesulfonyl chloride (0.12 mL, 1.51 mmol, 1.5 equiv) was added dropwise over ca. 1 min at 0 °C. The reaction mixture was stirred for 1 h 20 min at 0 °C. TLC analysis (40% ethyl acetate in cyclohexane, $KMnO_4$) showed full consumption of the starting material **38**. Then, 5 mL of water was added, and the reaction mixture was transferred to a separatory funnel containing 20 mL of water and 30 mL of dichloromethane. After layer separation, the organic layer was collected, and the aqueous layer was extracted with dichloromethane (2 × 30 mL). Combined organic layers were dried over anhydrous $MgSO_4$, and the dried solution was filtered through cotton and concentrated in vacuo. The residue was purified by flash-column chromatography (gradient elution with 0–30% ethyl acetate in cyclohexane) to afford β-deutero enone **1**-*d* as a white solid (195 mg, 80%).

β-Deutero enone **1**-*d*: **TLC** (40% ethyl acetate in cyclohexane, $KMnO_4$): R_f = 0.61. **^{1}H NMR** (500 MHz, $CDCl_3$) δ: 5.99 (dd, J = 2.9, 1.5 Hz, 1H), 4.50 (dd, J = 9.1, 2.7 Hz, 1H), 4.04 (ddd, J = 13.7, 9.2, 4.9 Hz, 1H), 3.32 (s, 3H), 3.26 (s, 3H), 2.73 (ddd, J = 16.5, 4.9, 1.1 Hz, 1H), 2.48 (dd, J = 16.5, 13.4 Hz, 1H), 1.37 (s, 3H), 1.33 (s, 3H). **^{13}C{^{1}H} NMR** (126 MHz, $CDCl_3$) δ: 196.9, 148.3 (t, J = 24.9 Hz), 130.2, 101.0, 99.9, 69.4, 68.3, 48.3, 48.3, 42.2, 17.9, 17.8. **FTIR** (neat), cm^{-1}: 3004, 2967, 2953, 2930, 2855, 2837, 1673, 1455, 1429, 1380, 1344, 1282, 1265, 1251, 1216, 1197, 1171, 1143, 1126, 1093, 1075, 1046, 1034, 1011, 943, 922, 907, 882, 875, 848, 791, 684, 551, 512, 468, 453, 437. **HRMS** (APCI+) *m/z*: $[M + H]^+$ Calcd for $C_{12}H_{18}DO_5$ 244.1290; Found 244.1288.

Synthesis of α'-Fluoro-β-deutero Enone 10-*d*

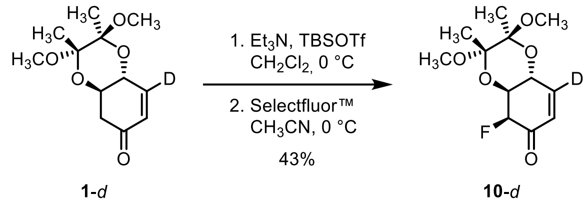

Triethylamine (0.45 mL, 3.21 mmol, 4.0 equiv) was added dropwise over ca. 1 min to a stirred solution of β-deutero enone **1**-*d* (195 mg, 0.803 mmol, 1 equiv) in dichloromethane (4.0 mL) at 23 °C. The reaction flask was placed in a 0 °C cooling bath (ice–sodium chloride–water). Then, *tert*-butyldimethylsilyl trifluoromethanesulfonate (0.28 mL, 1.21 mmol, 1.5 equiv) was added dropwise over ca. 1 min at 0 °C. The reaction mixture was stirred at 0 °C for 2 h. The reaction flask was removed from the 0 °C cooling bath, and the mixture was stirred at 23 °C for a further 1 h. Due to incomplete conversion, additional portions of triethylamine (0.22 mL, 1.61 mmol, 2.0 equiv) and *tert*-butyldimethylsilyl trifluoromethanesulfonate (0.18 mL, 0.80 mmol, 1.0 equiv) were added at 23 °C. The reaction mixture was stirred for an additional 1 h until TLC analysis (20% ethyl acetate in cyclohexane, $KMnO_4$) showed full consumption of the starting material **1**-*d*. The reaction mixture was concentrated in vacuo (rotavap) and then under high vacuum for ca. 10 min. The reaction flask containing the crude TBS enol ether (not depicted) was purged with argon, and anhydrous acetonitrile (6.0 mL) was added. The resulting solution was cooled in a 0 °C cooling bath (ice–sodium chloride–water), and the mixture was stirred at this temperature for 10 min. Selectfluor™ (427 mg, 1.205 mmol, 1.5 equiv) was added in one portion at 0 °C, and the reaction mixture was stirred for 1 h at this temperature. TLC analysis (20% ethyl acetate in cyclohexane) showed full consumption of the TBS enol ether, with minor hydrolysis of the silyl enol ether detected. The reaction flask was removed from the 0 °C cooling bath, and the reaction mixture was concentrated in vacuo. The residue was diluted with dichloromethane (40 mL) and transferred to a separatory funnel containing 40 mL of water. After extraction, the organic layer was separated, and the aqueous layer was extracted with dichloromethane (2 × 40 mL). Combined organic layers were dried over anhydrous $MgSO_4$, and the dried solution was filtered through cotton and concentrated in vacuo. The residue was purified by flash column chromatography (gradient elution with 0–10% ethyl acetate in cyclohexane) to afford α'-fluoro-β-deutero enone **10**-*d* as a white solid (90 mg, 43%).

<u>α'-Fluoro β-deutero enone **10**-*d*:</u> **TLC** (20% ethyl acetate in cyclohexane, $KMnO_4$): R_f = 0.44. **^{1}H NMR** (500 MHz, $CDCl_3$) δ: 6.11–6.04 (m, 1H), 4.87 (dd, J = 8.5, 2.6 Hz, 1H), 4.81 (app dt, J = 51.9, 1.7 Hz, 1H), 4.00 (ddd, J = 34.9, 8.5, 2.1 Hz, 1H), 3.33 (s, 3H), 3.28 (s, 3H), 1.39 (s, 3H), 1.36 (s, 3H). **^{13}C{^{1}H} NMR** (126 MHz, $CDCl_3$) δ: 190.5 (d, J = 16.6 Hz), 149.7 (t, J = 25.5 Hz), 127.7, 101.1, 100.7, 89.9 (d, J = 182.5 Hz), 71.3 (d, J = 18.0 Hz), 64.0 (d, J = 7.4 Hz), 48.4, 48.4, 17.8, 17.6. **^{19}F NMR** (471 MHz, $CDCl_3$) δ: −206.5 (dd, J = 52.3, 34.9 Hz). **FTIR** (neat), cm^{-1}: 3004, 2975, 2949, 2921, 2890, 2842, 1688, 1541, 1472, 1457, 1381,

1263, 1245, 1214, 1169, 1132, 1113, 1104, 1048, 1033, 1006, 959, 927, 914, 882, 853, 827, 789, 726, 663, 569, 446, 420. **HRMS** (APCI+) *m/z*: $[M - CH_3OH + H]^+$ Calcd for $C_{11}H_{13}DFO_4$ 230.0933; Found 230.0934.

Expanded Table for Scheme 6 with Fragment Coupling Conditions

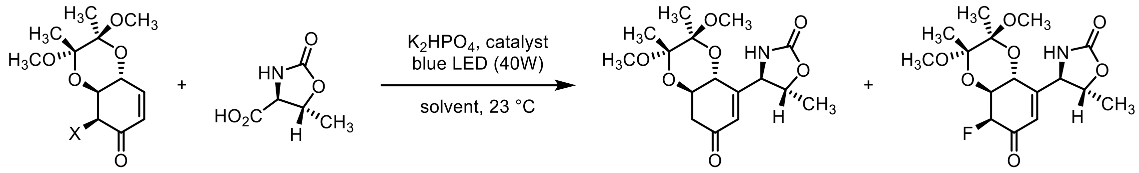

10 (X = F)
32 (X = Cl)
33 (X = OH)
34 (X = OAc)

2 **11** **24**

Entry	Reaction note	Equivalents		Catalyst	Solvent	Isolated yield	
		enone	2			11	24
1[a]	early stop[b] (**10**, X = F)	1	1.2	[Ir^{III}]	DMF	3%	13%
2[a]		1	1.2	4-CzIPN	DMF	11%	11%
3[a]	late stop[c] (**10**, X = F)	1	1.2	[Ir^{III}]	DMF	29%	ca. 10%[d]
4[a]	full conversion of enone **10** (X = F)[e]	1	1.2	[Ir^{III}]	DMF	35%	–
5[a]		1	1.2	4-CzIPN	DMF	32%	–
6[e]	excess **10**	2.0	1	[Ir^{III}]	DMF	43%	–
7[f]		4.0	1	[Ir^{III}]	DMF	ca. 22%[d]	36%
8[e]		4.0	1	[Ir^{III}]	DMF	47%	ca. 24%[d]
9[a]	excess **2**	1	3.0	[Ir^{III}]	DMF	33%	–
10[a]	internal standard[g] (**10**, X = F)	1	1.2	[Ir^{III}]	DMSO-d_6	45%[h]	–
11[a]	enone **32** (X = Cl)	1	1.2	[Ir^{III}]	DMF	ca. 15%[d]	N/A
12[a][g]		1	1.2	[Ir^{III}]	DMSO-d_6	11%[h]	N/A
13[a]	enone **33** (X = OH)	1	1.2	[Ir^{III}]	DMF	–	N/A
14[a][g]		1	1.2	[Ir^{III}]	DMSO-d_6	–	N/A
15[a]	enone **34** (X = OAc)	1	1.2	[Ir^{III}]	DMF	ca. 8%[d]	N/A
16[a][g]		1	1.2	[Ir^{III}]	DMSO-d_6	26%[h]	N/A
17	acridinium catalyst (**10**, X = F)	1	1.2	Mes-MeAcr	DMSO	no reaction	
18	enones **10** and **1**	1:1	1.0	[Ir^{III}]	DMF	see below for details	
19	Na_2HPO_4 as base (**10**, X = F)	1	1.2	[Ir^{III}]	DMF	mostly decomposition[i]	
20	DBU as base (**10**, X = F)	1	1.2	[Ir^{III}]	DMF	no consumption of **10**	

[a] Standard conditions: enone (**10**, **32**, **33**, or **34**), acid **2**, 1.2 equiv K_2HPO_4, and 1 mol% of photocatalyst. [b] Unreacted enone **10** observed as the major component of the reaction mixture (TLC analysis). [c] Enone **10** almost fully consumed (TLC analysis). [d] Indicated yield is approximate due to inseparable impurities. [e] Reaction was allowed to proceed overnight (16–19 h). [f] Reaction time was 1 h. [g] 1,3,5-Trimethoxybenzene (0.35 equiv) was used as an internal NMR standard. [h] Yield determined by NMR against 1,3,5-trimethoxybenzene as the internal standard. [i] Trace fluorinated product **24** isolated (ca. 6%, impure).
Abbreviations: 4-CzIPN = 2,4,5,6-tetrakis(9*H*-carbazol-9-yl)isophthalonitrile, [Ir^{III}] = catalyst **3**, Mes-MeAcr = 9-mesityl-10-methylacridinium perchlorate.

Competition Experiment

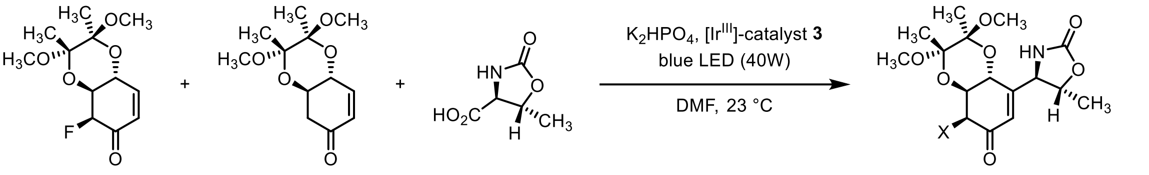

10 (1 equiv) **1** (1.0 equiv) **2** (1.0 equiv) **11** (X = H, major) + **24** (X = F, minor)

Pseudoaxial fluorinated enone **10** (100 mg, 0.384 mmol, 1 equiv), enone **1** (93 mg, 0.384 mmol, 1.0 equiv) oxazolidinone **2** (56 mg, 0.384 mmol, 1.0 equiv), dipotassium phosphate (67 mg, 0.384 mmol, 1.0 equiv), and [Ir^{III}]-catalyst **3** (4.3 mg, 0.0038 mmol, 0.01 equiv) were added to the reaction flask. The flask was evacuated and backfilled with argon (3 cycles). Anhydrous *N*,*N*-dimethylformamide (3.0 mL) was added at 23 °C, and the reaction mixture was degassed by bubbling argon through the solution for 20 min (outlet needle) with stirring at 23 °C. A double-layered balloon filled with argon was attached, and the reaction mixture was stirred for 2 h at 23 °C (a cooling fan was set up to maintain the ambient temperature of the reaction mixture) while being irradiated by a blue LED lamp (40W Kessil® A160WE Tuna Blue lamp) placed about 4 cm away from the reaction flask. TLC analyses (20% acetone in dichloromethane and 20% ethyl acetate in cyclohexane, $KMnO_4$) showed the formation of two new UV-active spots and the preferential consumption of the fluorinated enone **10**. The blue LED was turned off, and the reaction mixture was transferred to a separatory funnel containing 20 mL of ethyl acetate and 20 mL of ice-cold water. The mixture was extracted, the organic layer was separated, and the aqueous layer was extracted with ethyl acetate (2 × 20 mL). Combined organic layers were washed with ice-cold water (3 × 30 mL), 10% aqueous lithium chloride solution (2 × 30 mL), and brine (1 × 30 mL). The washed organic layer was dried over anhydrous $MgSO_4$. The dried solution was filtered through cotton and concentrated in vacuo. A ^1H NMR spectrum of the residual crude mixture was measured. The fluorinated enone **10** was consumed preferentially (see NMR spectra, page S66) and the major products observed corresponded to the atypical fluorinated and non-fluorinated adducts **24** and **11**, respectively.

Radical Trapping Experiment

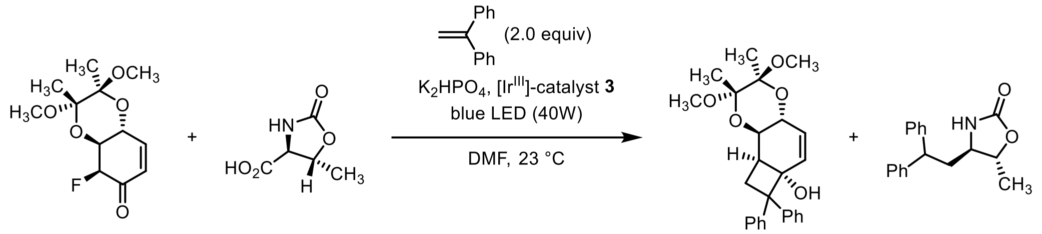

10 (1 equiv) **2** (1.2 equiv) **39** (9% from **10**) **40** (26% from **2**)

Pseudoaxial fluorinated enone **10** (100 mg, 0.384 mmol, 1 equiv), oxazolidinone **2** (67 mg, 0.461 mmol, 1.2 equiv), dipotassium phosphate (80 mg, 0.192 mmol, 1.2 equiv), and [Ir^{III}]-catalyst **3** (4.3 mg, 0.0038 mmol, 0.01 equiv) were added to a reaction flask. The flask was evacuated and backfilled with argon (3 cycles). Anhydrous *N*,*N*-dimethylformamide (3.0 mL) and 1,1-diphenylethylene (0.13 mL, 0.77 mmol, 2.0 equiv) were added at 23 °C, and the reaction mixture was degassed by bubbling argon through the solution for 20 min (outlet needle) with stirring at 23 °C. A double-layered balloon filled with argon was attached, and the reaction mixture was stirred for 70 h at 23 °C (a cooling fan was set up to maintain the ambient temperature of the reaction mixture) while being irradiated by a blue LED lamp (40W Kessil® A160WE Tuna Blue lamp) placed about 4 cm away from the reaction flask. TLC analysis (40% ethyl acetate in cyclohexane, $KMnO_4$) showed full consumption of starting material **10**. The blue LED was turned off, and the reaction mixture was transferred to a separatory funnel containing 20 mL of ethyl acetate and 20 mL of ice-cold water. The mixture was extracted, the organic layer was separated, and the aqueous layer was extracted with ethyl acetate (2 × 20 mL). Combined organic layers were washed with ice-cold water (3 × 30 mL), 10% aqueous solution of LiCl (2 × 30 mL) and brine (1 × 30 mL). The washed organic layer was dried over anhydrous $MgSO_4$, and the dried solution was filtered through cotton and concentrated in vacuo. The residue was purified by flash-column chromatography (40% ethyl acetate in cyclohexane) to afford separately adducts **39** and **40**, both containing several impurities. The first fraction containing **39** was repurified by a second flash-column chromatography (20% ethyl acetate in cyclohexane) to afford cyclobutane **39** as a white solid (15 mg, 9% from **10**). The second fraction containing **40** was purified by a second flash-column chromatography (5% acetone in dichloromethane) to afford oxazolidinone **40** as an off-white solid (33 mg, 26% from **2**).

Cyclobutane **39:** **TLC** (20% ethyl acetate in cyclohexane, $KMnO_4$): R_f = 0.29. ^{1}**H NMR** (500 MHz, $CDCl_3$) δ: 7.62–7.59 (m, 2H), 7.38–7.33 (m, 2H), 7.31–7.26 (m, 2H), 7.25–7.21 (m, 3H), 7.19–7.14 (m, 1H), 5.95 (ddd, *J* = 10.4, 2.7, 1.6 Hz, 1H), 5.68 (dd, *J* = 10.4, 1.5 Hz, 1H), 4.33 (ddd, *J* = 9.2, 2.7, 1.6 Hz, 1H), 3.82 (dd, *J* = 9.2, 4.1 Hz, 1H), 3.24 (s, 3H), 3.21 (s, 3H), 3.03 (dd, *J* = 12.2, 8.7 Hz, 1H), 2.72 (dddd, *J* = 10.5, 8.6, 4.3, 1.6 Hz, 1H), 2.14 (t, *J* = 11.6 Hz, 1H), 2.04 (s, 1H), 1.33 (s, 3H), 1.31 (s, 3H). ^{13}**C{^{1}H} NMR** (126 MHz, $CDCl_3$) δ: 146.1, 140.2, 130.7, 129.0 (2C), 128.6 (2C), 128.5, 128.1 (2C), 127.8 (2C), 127.2, 126.2, 100.6,

100.4, 78.2, 66.8, 66.2, 62.7, 48.1, 47.9, 42.8, 25.1, 18.0, 18.0. **FTIR** (neat), cm^{-1}: 3455, 3056, 3022, 2993, 2949, 2926, 2853, 2833, 1773, 1733, 1716, 1699, 1684, 1598, 1494, 1457, 1448, 1376, 1213, 1132, 1111, 1035, 1007, 949, 908, 883, 862, 848, 748, 735, 702, 676, 659, 608, 541. **HRMS** (APCI−) *m/z*: $[M + Cl]^-$ Calcd for $C_{26}H_{30}O_5Cl$ 457.1787; Found 457.1793.

Oxazolidinone **40:** **TLC** (5% acetone in dichloromethane, $KMnO_4$): R_f = 0.42. **^{1}H NMR** (500 MHz, $CDCl_3$) δ: 7.33–7.29 (m, 4H), 7.25–7.19 (m, 6H), 5.00 (br s, 1H), 4.33 (app quint, *J* = 6.3 Hz, 1H), 3.98 (t, *J* = 8.0 Hz, 1H), 3.38–3.26 (m, 1H), 2.31 (dd, *J* = 8.0, 6.5 Hz, 2H), 1.35 (d, *J* = 6.3 Hz, 3H). **^{13}C{^{1}H} NMR** (126 MHz, $CDCl_3$) δ: 158.7, 143.5, 143.2, 129.1 (2C), 129.1 (2C), 127.8 (2C), 127.7 (2C), 127.2, 127.1, 79.1, 58.8, 48.9, 40.9, 19.9. **FTIR** (neat), cm^{-1}: 3255, 3061, 3027, 2978, 2929, 1739, 1678, 1599, 1494, 1451, 1386, 1248, 1231, 1142, 1112, 1053, 976, 947, 908, 790, 771, 730, 698, 647, 616, 595, 567. **HRMS** (APCI+) *m/z*: $[M + H]^+$ Calcd for $C_{18}H_{20}NO_2$ 282.1489; Found 282.1487.

Classic Giese Reaction with the α'-Hydroxy Enone 33

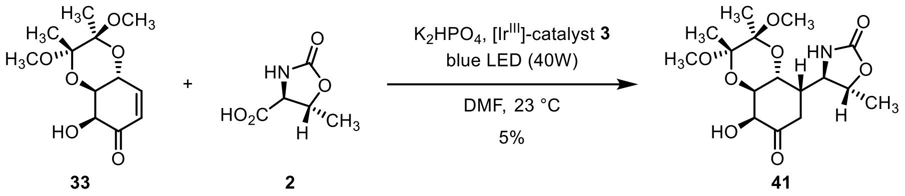

Oxazolidinone **2** (86 mg, 0.590 mmol, 1.2 equiv), dipotassium phosphate (103 mg, 0.590 mmol, 1.2 equiv), and [Ir^{III}]-catalyst **3** (5.5 mg, 0.0049 mmol, 0.01 equiv) were added to the reaction flask. The flask was evacuated and backfilled with argon (3 cycles). A solution of α'-hydroxy enone **33** (127 mg, 0.491 mmol, 1 equiv) in anhydrous *N*,*N*-dimethylformamide (4.0 mL) was added at 23 °C, and the reaction mixture was degassed by bubbling argon through the solution for 20 min (outlet needle) with stirring at 23 °C. A double-layered balloon filled with argon was attached, and the reaction mixture was stirred for 4 h at 23 °C (a cooling fan was set up to maintain the ambient temperature of the reaction mixture) while being irradiated by a blue LED lamp (40W Kessil® A160WE Tuna Blue lamp) placed about 4 cm away from the reaction flask. TLC analysis (20% acetone in dichloromethane, $KMnO_4$) showed full consumption of starting material **33**. The blue LED was turned off, and the reaction mixture was transferred to a separatory funnel containing 20 mL of ethyl acetate and 20 mL of ice-cold water. The mixture was extracted, the organic layer was separated, and the aqueous layer was extracted with ethyl acetate (2 × 30 mL). Combined organic layers were washed with ice-cold water (3 × 30 mL), 10% aqueous solution of LiCl (2 × 30 mL) and brine (1 × 30 mL). The washed organic layer was dried over anhydrous $MgSO_4$. The dried solution was filtered through cotton and concentrated in vacuo. The residue was purified by flash-column chromatography (20% acetone in dichloromethane) to afford Giese product **41** as a yellow solid (9 mg, 5%).

Giese product **41:** **TLC** (20% ethyl acetate in cyclohexane, $KMnO_4$): R_f = 0.43. **^{1}H NMR** (500 MHz, $CDCl_3$) *δ*: 5.90 (br s, 1H), 4.60 (dd, *J* = 10.8, 5.0 Hz, 1H), 4.33 (app quint, *J* = 6.3 Hz, 1H), 4.10 (d, *J* = 2.1 Hz, 1H), 3.88 (dd, *J* = 10.8, 3.1 Hz, 1H), 3.34 (dd, *J* = 11.1, 6.4 Hz, 1H), 3.29 (s, 3H), 3.24 (s, 3H), 3.17 (dd, *J* = 14.2, 6.3 Hz, 1H), 2.80 (br s, 1H), 2.36 (ddd, *J* = 11.3, 6.9, 2.4 Hz, 1H), 2.17 (dt, *J* = 14.2, 1.6 Hz, 1H), 1.48 (d, *J* = 6.3 Hz, 3H), 1.33 (s, 3H), 1.32 (s, 3H). **^{13}C{^{1}H} NMR** (126 MHz, $CDCl_3$) *δ*: 205.0, 157.8, 100.3, 99.9, 77.7, 76.1, 67.9, 67.2, 58.6, 48.5, 48.5, 41.2, 36.6, 21.0, 18.0, 17.5. **FTIR** (neat), cm^{-1}: 3438, 2923, 2853, 1727, 1457, 1412, 1378, 1322, 1249, 1211, 1131, 1113, 1077, 1036, 976, 932, 888, 871, 848, 767, 735, 701, 580, 526, 506, 474, 430. **HRMS** (APCI−) *m/z*: $[M + Cl]^-$ Calcd for $C_{16}H_{25}NO_8Cl$ 394.1274; Found 394.1268.

X-Ray Crystallography

Diffraction data were collected on a Rigaku OD (Oxford Diffraction) Synergy DW Custom diffraction system with a hybrid photon counting X-ray detector HyPix-6000HE using Mo Kα radiation generated by a microfocus rotating anode X-ray generator MicroMax-007HF DW. *CrysAlisPro* was used for data collection and data reduction.[5] The structures were solved by the direct methods and refined by full matrix least-squares methods on F^2 using *SHELXT* and *SHELXL*.[6,7] All non-hydrogen atoms were refined anisotropically and the hydrogen atoms were refined as riding on their carrier atoms. Crystal data and refinement parameters are gathered in Table S1. The supplementary crystallographic data for this paper can be obtained free of charge from The Cambridge Crystallographic Data Centre using https://www.ccdc.cam.ac.uk/structures/.

Table S1. Crystallographic information for compounds **10** and **24**.

Compound	**10**	**24**
CCDC No.	2534271	2534272
Crystal data		
Chemical formula	$C_{12}H_{17}FO_5$	$C_{16}H_{22}FNO_7$
M_r	260.25	359.34
Crystal system, space group	Monoclinic, $P2_1$	Orthorhombic, $P2_12_12_1$
Temperature (K)	120.02(10)	120.02(10)
a (Å)	6.78375(8)	9.32963(6)
b (Å)	7.11224(10)	10.58137(7)
c (Å)	12.57793(16)	17.36413(11)
α (°)	90	90
β (°)	91.9456(11)	90
γ (°)	90	90
V (Å^3)	606.506(14)	1714.191(19)
Z	2	4
μ (mm^{-1})	1.025	0.992
Crystal size (mm)	0.300 × 0.200 × 0.050	0.300 × 0.200 × 0.100
Data collection		
Absorption correction	Multi-scan; CrysAlisPro 1.171.44.115a (Rigaku Oxford Diffraction, 2025). Empirical absorption correction using spherical harmonics, implemented in SCALE3 ABSPACK scaling algorithm.	Multi-scan; CrysAlisPro 1.171.44.117a (Rigaku Oxford Diffraction, 2025). Empirical absorption correction using spherical harmonics, implemented in SCALE3 ABSPACK scaling algorithm.
T_{min}/T_{max}	0.65375	0.86285
Measured, unique, observed [$I > 2\sigma(I)$] data	6734, 2264, 2217	24154, 3619, 3600
R_{int}	0.0298	0.0201
$(\sin \theta/\lambda)_{max}$ (Å^{-1})	0.6328	0.6332
Refinement		
$R[F^2 > 2\sigma(F^2)]$, $wR(F^2)$, S	0.0298, 0.0812, 1.057	0.0254, 0.0696, 1.055
Reflections, parameters, restraints	2264, 168, 1	3619, 232, 0
$\Delta\rho_{max}$, $\Delta\rho_{min}$ (e Å^{-3})	0.258, −0.163	0.252, −0.177

Figure S1. Molecular structure of **10**. Thermal ellipsoids are drawn at the 50% probability level.

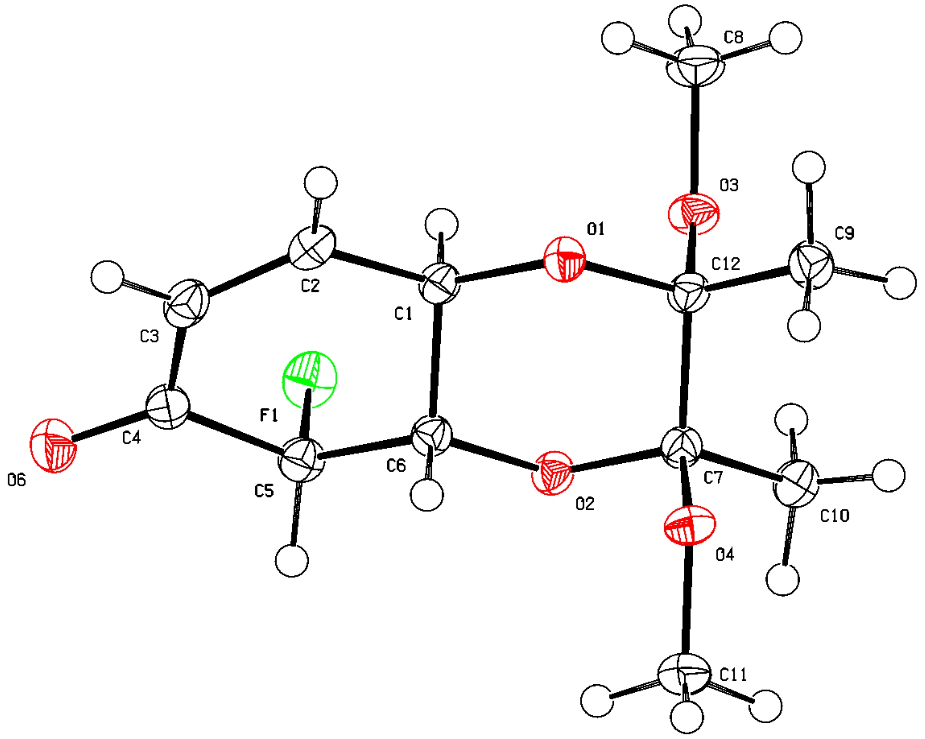

Figure S2. Molecular structure of **24**. Thermal ellipsoids are drawn at the 50% probability level.

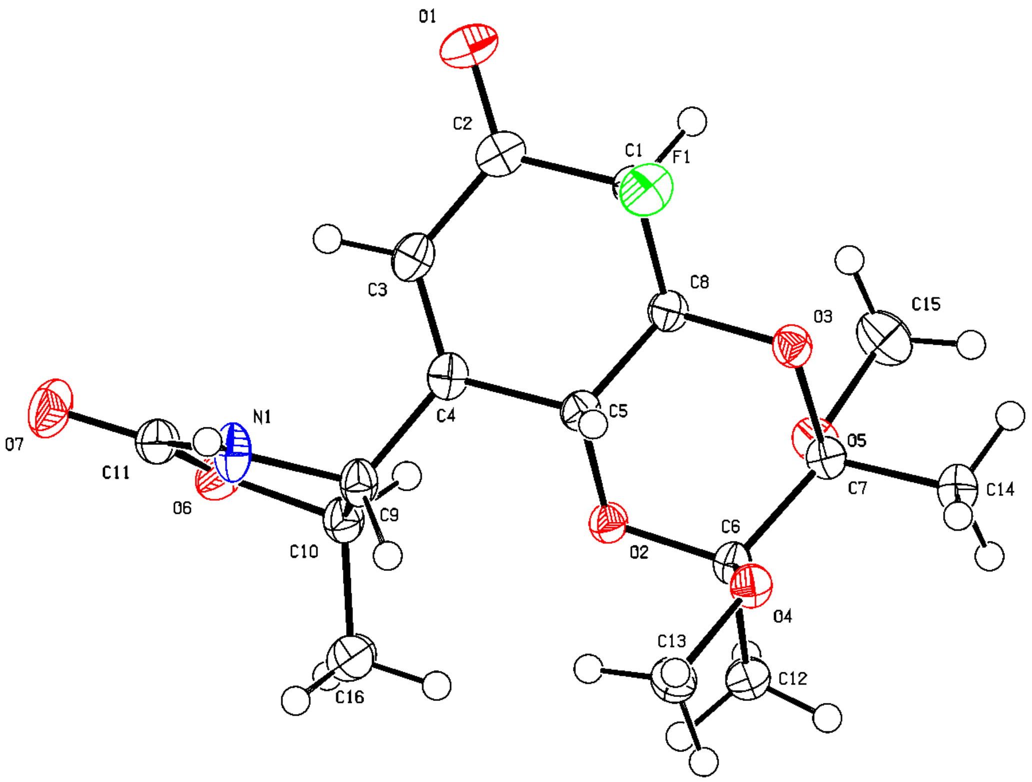

Computational Data

Computations were performed with the 6-31+G** basis set of atomic orbitals using Becke's three parameter exchange[8] and Lee-Yang-Parr's correlation functional.[9] All computations were executed with the Gaussian 09 program package.[10] All intermediates and transition states found were verified with frequency calculations. Reaction free energies were calculated for optimized geometries computed assuming *N*,*N*-dimethylformamide as solvent, represented with the PCM solvation method.[11]

Optimized structures mentioned in the paper:

27, epimer 1
Total electronic energy: -1304.75666878 Hartree
Number of imaginary frequencies: 0

F	-1.25789500	2.63313200	-1.79345300
O	0.70639300	4.36222800	-0.32192800
O	-0.46343000	-1.01109000	0.14674200
O	-2.52909300	0.86119600	-0.07967600
O	-2.01804300	-1.47395100	-1.54437600
O	-2.66700400	-0.49136600	1.82360200
O	3.68391100	-1.40545000	1.09611300
O	5.52925900	-0.08332900	1.14887900
N	3.70874800	0.36820100	-0.22300900
C	-1.00287800	2.67178600	-0.39834800
H	-1.69225800	3.40701800	0.02242900
C	0.44605200	3.14419200	-0.23413200
C	1.46684500	2.17085000	-0.00952300
C	1.23840300	0.70385400	0.16957500
C	-0.16464700	0.30603000	-0.32524700
H	-0.18801400	0.31148500	-1.42222300
C	-1.79238200	-1.47221300	-0.14918300
C	-2.83745300	-0.44759100	0.42045600
C	-1.20413600	1.29557400	0.20548100
H	-1.07423800	1.39404700	1.29374000
C	2.37895100	-0.16699700	-0.46196500
H	2.19313900	-0.26338900	-1.53599300
C	2.53699700	-1.56916500	0.19866700
H	1.68521700	-1.81324600	0.82987800
C	4.41999200	-0.33183300	0.70209900
C	-1.89217800	-2.85467400	0.48518000
H	-2.84480200	-3.32373100	0.22747900
H	-1.81792900	-2.77208500	1.56922300
H	-1.07912600	-3.48967600	0.12657400
C	-1.26651600	-2.40317700	-2.33085600
H	-1.61997600	-3.42994300	-2.18827600
H	-0.19800000	-2.34844300	-2.10196000
H	-1.42592300	-2.11181800	-3.37031200
C	-4.26813500	-0.72924500	-0.02435800
H	-4.62103000	-1.67139000	0.40196600
H	-4.31506500	-0.79004600	-1.11122300
C	-3.55302500	0.31257300	2.61181900
H	-3.12762800	0.32757100	3.61663400
H	-4.55703000	-0.12287700	2.65483100
C	2.83611400	-2.68902300	-0.78473900
H	3.06680600	-3.61118100	-0.24458500
H	3.68605800	-2.43468300	-1.42632900

H	1.96469400	-2.87186600	-1.41999000
H	-3.61336500	1.33655100	2.22908600
H	-4.92649300	0.07621000	0.30856000
H	4.13056500	1.13191400	-0.72969800
H	2.47241400	2.56219500	0.10882900
H	1.27382800	0.52023600	1.25910700

27, epimer 2
Total electronic energy: -1304.75365209 Hartree
Number of imaginary frequencies: 0

F	-1.01932600	3.29676100	-0.45142200
O	1.78313800	3.79095400	0.56374800
O	-0.76662000	-0.87904100	-0.89059800
O	-2.09623700	1.00628600	0.68824300
O	-2.88780000	-0.30757300	-1.69593900
O	-1.96892300	-1.11543200	1.65964500
O	3.71784300	-1.11566300	1.02347000
O	5.50750400	-1.22663400	-0.36143200
N	3.36209400	-0.79993200	-1.14134600
C	-0.29008200	2.57116100	0.52529900
H	-0.51665300	3.03274100	1.48879800
C	1.19499000	2.75658500	0.18488200
C	1.85780800	1.74751900	-0.57813100
C	1.23062600	0.49141600	-1.08682800
C	-0.29660200	0.46991700	-0.84261200
H	-0.77836200	1.04352300	-1.64208300
C	-2.18221200	-1.02034800	-0.69953700
C	-2.59619800	-0.33934700	0.65656400
C	-0.68950100	1.10741700	0.48820400
H	-0.16699400	0.60640800	1.31508600
C	2.00353600	-0.79347900	-0.62115200
H	1.44049000	-1.64989900	-0.99879400
C	2.26846700	-0.95745100	0.90872500
H	2.03262500	-0.04077000	1.45577200
C	4.30720900	-1.06532900	-0.19984200
C	-2.44926500	-2.52121700	-0.72872900
H	-3.52330100	-2.71729900	-0.68511200
H	-1.96406900	-3.00300500	0.11952400
H	-2.05045800	-2.95180800	-1.64992400
C	-2.73246800	-0.75354500	-3.04826000
H	-3.28253900	-1.68322900	-3.22921000
H	-1.67778100	-0.89529600	-3.30533000
H	-3.15275600	0.03661900	-3.67259700
C	-4.10468800	-0.21886200	0.83827700
H	-4.55821800	-1.20917400	0.92471500
H	-4.54176100	0.29993000	-0.01438300
C	-2.23692300	-0.75755200	3.02105800

H	-1.50290400	-1.29757100	3.62135600
H	-3.24324000	-1.06513300	3.32449100
C	1.57836400	-2.15090000	1.54211800
H	1.87112500	-2.24484300	2.59156300
H	1.84507000	-3.07355300	1.01661500
H	0.49335900	-2.01868900	1.49149000
H	-2.11704800	0.31836100	3.18401800
H	-4.32598500	0.35021600	1.74386100
H	3.57825100	-0.92980900	-2.11996900
H	1.37967800	0.48768200	-2.17786700
H	2.91114200	1.91416700	-0.78713000

8, epimer 1
Total electronic energy: -1205.52405694 Hartree
Number of imaginary frequencies: 0

O	0.86155500	4.37640800	-0.87601400
O	-0.54998900	-0.86274500	0.13735700
O	-2.53677400	1.06220800	-0.26525800
O	-2.08458300	-1.40033000	-1.55086500
O	-2.79256400	-0.15553900	1.71897800
O	3.56846500	-1.31869400	1.21279500
O	5.46385600	-0.07062300	1.13735700
N	3.67009700	0.29464000	-0.29399700
C	-0.94258900	2.79042700	-0.69187000
H	-1.55416800	3.59780200	-0.27904700
C	0.52045500	3.20028000	-0.62996900
C	1.51080500	2.21336900	-0.29485600
C	1.21854500	0.78660500	0.05071400
C	-0.19969900	0.41104500	-0.41525200
H	-0.22083700	0.34397100	-1.51230300
C	-1.88807600	-1.29651800	-0.15317800
C	-2.90964800	-0.19448800	0.30714100
C	-1.19861800	1.47573700	0.03377300
H	-1.10211500	1.61368000	1.11917300
C	2.31468800	-0.20032000	-0.47498400
H	2.12499200	-0.40789100	-1.53267500
C	2.41139900	-1.52642500	0.33677800
H	1.55160900	-1.65896300	0.99009200
C	4.34731200	-0.32351900	0.70974200
C	-2.05416400	-2.62212300	0.58147700
H	-3.01852500	-3.07485900	0.33840000
H	-1.99960000	-2.45904600	1.65748300
H	-1.25878400	-3.31259600	0.29239900
C	-1.35427100	-2.41360200	-2.24706500
H	-1.74178700	-3.41419000	-2.02690300
H	-0.28689600	-2.37437000	-2.00965400
H	-1.49221200	-2.20423800	-3.30925700

C	-4.33613500	-0.45615500	-0.16528400
H	-4.73376000	-1.36009500	0.30253400
H	-4.35493400	-0.57756900	-1.24794200
C	-3.64493400	0.76000700	2.41513500
H	-3.25667800	0.81445700	3.43373400
H	-4.67959200	0.40130600	2.44648900
C	2.65385700	-2.76134500	-0.51546900
H	2.84295200	-3.62839000	0.12323300
H	3.51211100	-2.61967400	-1.18045100
H	1.77173600	-2.97101500	-1.12711300
H	-3.61687200	1.75705600	1.96369200
H	-4.97569000	0.38818600	0.10171600
H	4.11205500	1.00544100	-0.85705200
H	2.53227400	2.57439200	-0.22883000
H	1.24107300	0.71789700	1.15354300
H	-1.22031700	2.68290200	-1.74907000

8, epimer 2
Total electronic energy: -1205.52150856 Hartree
Number of imaginary frequencies: 0

O	1.88551700	3.90278700	0.11941200
O	-0.85459400	-0.78577100	-0.86249400
O	-2.08141400	1.25717000	0.59591900
O	-2.93736600	-0.15565300	-1.72197600
O	-2.10640800	-0.81008700	1.69183700
O	3.67187400	-0.95870200	1.10178100
O	5.42704700	-1.31213600	-0.28702400
N	3.28008900	-0.91652800	-1.07864100
C	-0.20297900	2.72152100	0.33688600
H	-0.32657500	3.22798800	1.29818600
C	1.24924300	2.85094500	-0.10327400
C	1.87612600	1.75434400	-0.79019000
C	1.20609900	0.46716100	-1.14715600
C	-0.31367000	0.53825100	-0.88041700
H	-0.77349800	1.09775800	-1.70571800
C	-2.27489800	-0.83925700	-0.67381700
C	-2.66279700	-0.05046500	0.63155600
C	-0.65925100	1.26897700	0.41167600
H	-0.19046800	0.76623100	1.26636100
C	1.93356200	-0.78344700	-0.54168800
H	1.32846600	-1.65553500	-0.80088200
C	2.22571200	-0.76901500	0.99155300
H	2.02759800	0.21482400	1.42413900
C	4.23568300	-1.08911000	-0.12799200
C	-2.62397900	-2.32247100	-0.61300800
H	-3.70721300	-2.45714000	-0.56320200
H	-2.16799500	-2.77785800	0.26547800

H	-2.24750000	-2.82960700	-1.50425900
C	-2.78674200	-0.68859200	-3.04237400
H	-3.37936100	-1.59993300	-3.17749000
H	-1.73711300	-0.89650300	-3.27403600
H	-3.15960500	0.08173100	-3.71964000
C	-4.16459600	0.17043600	0.77854100
H	-4.68042500	-0.78442300	0.90607400
H	-4.55600100	0.67087900	-0.10671700
C	-2.34732600	-0.33780200	3.02187900
H	-1.66636800	-0.89693200	3.66593700
H	-3.37797700	-0.53412100	3.33681900
C	1.51305700	-1.85402100	1.77738500
H	1.81842400	-1.82607700	2.82709100
H	1.74518100	-2.84241000	1.36793100
H	0.43103600	-1.69876000	1.72369400
H	-2.13430300	0.73226500	3.11257200
H	-4.36407800	0.79729500	1.65052100
H	3.47189400	-1.17075400	-2.03753000
H	1.33136500	0.32949000	-2.23240100
H	2.92168900	1.88436400	-1.05612100
H	-0.82450900	3.25732700	-0.39331400

10
Total electronic energy: -943.487823707 Hartree
Number of imaginary frequencies: 0

F	-2.23797400	-0.07251500	1.91005600
O	-4.45694200	0.58811100	-0.10593200
O	0.62312100	-1.08347000	-1.02232800
O	0.20141400	0.98109700	0.84366300
O	1.85385200	-1.34915100	0.94823800
O	1.50725200	1.63365800	-0.98306400
C	-2.19706900	0.67472300	0.70441400
H	-2.40318500	1.71361100	0.96969900
C	-3.32862500	0.11302400	-0.18469600
C	-2.99102600	-1.00561800	-1.07786500
H	-3.80251200	-1.41197900	-1.67345800
C	-1.73734100	-1.49459700	-1.16688500
C	-0.61953700	-0.96684900	-0.32456300
H	-0.56547400	-1.55116700	0.60478000
C	1.74337100	-0.61169000	-0.25298000
C	1.49093900	0.87642300	0.21036600
C	-0.85655200	0.49880600	0.02646100
H	-0.89421900	1.07208500	-0.91079400
C	2.95445700	-0.75812700	-1.16706100
H	3.87113300	-0.50613800	-0.62851100
H	2.85420500	-0.09704800	-2.02726900
H	3.02764700	-1.78771500	-1.52417400

C	2.15491900	-2.74523700	0.83123100
H	3.20389500	-2.90652500	0.56099200
H	1.50683900	-3.23496900	0.09738500
H	1.97071300	-3.17310400	1.81800000
C	2.49427100	1.36142900	1.24987500
H	3.49744100	1.40647200	0.81920200
H	2.50328700	0.68383200	2.10293500
C	1.36670000	3.05414500	-0.85645300
H	1.17483200	3.42516600	-1.86455100
H	2.28416000	3.51404800	-0.47426400
H	0.52548700	3.31746100	-0.20737100
H	2.21555300	2.35839500	1.59822300
H	-1.50465500	-2.31033500	-1.84701500

28
Total electronic energy: -1304.29382307 Hartree
Number of imaginary frequencies: 0

F	-1.08868000	2.76208300	-1.72509400
O	0.83584200	4.26915300	0.17528300
O	-0.56236300	-1.09025800	0.08497700
O	-2.53286000	0.86526700	-0.26671100
O	-2.01393000	-1.58260200	-1.68994100
O	-2.93221200	-0.49931400	1.59303400
O	4.43117000	-0.94951700	0.89839800
O	5.63795500	-0.82466500	-1.01711800
N	3.38853600	-0.23298500	-0.92273000
C	-0.88352500	2.64309900	-0.28945600
H	-1.58816300	3.35824800	0.14324700
C	0.54712000	3.01695000	0.04300700
C	1.47730600	1.95434900	0.17099800
H	2.50340100	2.25081400	0.38645600
C	1.19388400	0.59466200	0.01040200
C	-0.20112400	0.22007200	-0.41239500
H	-0.29669800	0.19465300	-1.50988000
C	-1.87623500	-1.51153200	-0.27628300
C	-2.93520100	-0.43307400	0.17318500
C	-1.20474100	1.22589200	0.13472600
H	-1.14246800	1.20452600	1.23156700
C	2.26707300	-0.45534900	0.02296800
H	1.81051200	-1.42987000	-0.18733600
C	3.08743600	-0.55702300	1.33749100
H	3.18958900	0.43691700	1.78587200
C	4.57097600	-0.67806800	-0.43054200
C	-2.07659800	-2.86661400	0.39714800
H	-3.03633600	-3.30076600	0.10576200
H	-2.05202000	-2.75355400	1.48050900
H	-1.27758900	-3.54972300	0.09970100

C	-1.15581700	-2.49687400	-2.37678200
H	-1.47436700	-3.53560100	-2.23277000
H	-0.11431000	-2.38779300	-2.05722500
H	-1.23525700	-2.24566000	-3.43624700
C	-4.32121300	-0.66006300	-0.42271700
H	-4.74378500	-1.60137500	-0.06256300
H	-4.25918900	-0.69129500	-1.51008700
C	-3.82245200	0.38175600	2.28375500
H	-3.52010300	0.35200900	3.33231600
H	-4.86309600	0.04758300	2.20408800
C	2.57228300	-1.55520300	2.35214500
H	3.22301600	-1.58551600	3.23064900
H	2.51489600	-2.55851400	1.91839000
H	1.57044300	-1.25879400	2.67778900
H	-3.73958700	1.40862100	1.91318400
H	-4.98683300	0.15705800	-0.13491500
H	3.25246500	-0.18221500	-1.92282300

24
Total electronic energy: -1304.18396933 Hartree
Number of imaginary frequencies: 0

F	-0.96787000	2.94163900	-1.55348400
O	1.25614000	4.11099400	0.20644600
O	-0.53075100	-1.00530600	-0.14867400
O	-2.40658000	1.07439100	0.03441300
O	-2.29367100	-1.19945500	-1.67375600
O	-2.52412000	-0.44674500	1.80644800
O	3.65419300	-1.10886000	1.26800400
O	5.57090300	-0.21563400	0.44360900
N	3.59713900	-0.27374300	-0.78047600
C	-0.67387300	2.73835400	-0.18095800
H	-1.23952400	3.48600200	0.37834500
C	0.83991600	2.98002700	-0.02698300
C	1.73142100	1.82756100	-0.19922500
H	2.79404100	2.03779500	-0.13408300
C	1.28055300	0.57172300	-0.41822400
C	-0.19713000	0.31661900	-0.57962400
H	-0.44938200	0.41788400	-1.64478300
C	-1.92848400	-1.32054700	-0.31610200
C	-2.80672600	-0.24865200	0.43661000
C	-1.02042700	1.32283800	0.22051800
H	-0.75891900	1.21396000	1.28261400
C	2.20800400	-0.62255400	-0.56404200
H	1.83275300	-1.25674100	-1.37292300
C	2.33977200	-1.47532900	0.74716100
H	1.61437100	-1.16742100	1.49957700
C	4.39116000	-0.49527400	0.30111200

C	-2.09576100	-2.73022600	0.23787100
H	-3.11757400	-3.08238700	0.07783900
H	-1.88001800	-2.73870000	1.30565500
H	-1.40727700	-3.41314200	-0.26418400
C	-1.72373700	-2.13833900	-2.59432100
H	-2.18673900	-3.12562500	-2.49495900
H	-0.64076400	-2.22521200	-2.46117800
H	-1.93372600	-1.74356100	-3.58957300
C	-4.29057700	-0.34217300	0.10179600
H	-4.70362500	-1.28867500	0.45851900
H	-4.43383000	-0.27427200	-0.97614800
C	-3.24678300	0.35979100	2.74546600
H	-2.73806400	0.23009200	3.70202300
H	-4.28489000	0.02535900	2.84354000
C	2.28638800	-2.97462700	0.51137700
H	2.50019600	-3.50845200	1.44125000
H	3.01609600	-3.27861300	-0.24605700
H	1.28639300	-3.25491600	0.17020000
H	-3.22604200	1.41757300	2.46452200
H	-4.83060800	0.47908600	0.57797600
H	3.94678500	0.20117500	-1.60000100

29
Total electronic energy: -1304.18396933 Hartree
Number of imaginary frequencies: 0

O	1.34452400	4.22218400	-0.62778800
O	-0.61439500	-0.86490800	-0.18390200
O	-2.38081000	1.31672300	-0.08506700
O	-2.37229300	-1.02851000	-1.71899200
O	-2.61230800	-0.14045700	1.72965400
O	3.54823900	-0.99264500	1.36003900
O	5.50803000	-0.22497500	0.51126000
N	3.56334100	-0.34136500	-0.75505100
C	-0.53140800	2.85079000	-0.24477800
H	-1.22446200	3.68562800	-0.27062400
C	0.87678800	3.07477200	-0.45477800
C	1.75880500	1.88217800	-0.44621300
H	2.82595000	2.07907700	-0.43279800
C	1.27787800	0.62378600	-0.50974300
C	-0.21307000	0.42235500	-0.65305400
H	-0.47724900	0.51096600	-1.71744700
C	-2.02501100	-1.11429900	-0.35318600
C	-2.85777900	0.02900600	0.34814700
C	-0.98389400	1.48990700	0.12841400
H	-0.75927300	1.32638900	1.19863000
C	2.15608500	-0.61433600	-0.53766500
H	1.77048000	-1.29977800	-1.29870700

C	2.23315400	-1.36015800	0.83961000
H	1.50294100	-0.96817000	1.54713100
C	4.32387900	-0.48484500	0.36253400
C	-2.26845800	-2.49186400	0.25130700
H	-3.30422300	-2.80016600	0.09036900
H	-2.06621700	-2.47030600	1.32154200
H	-1.60748200	-3.22559600	-0.21487500
C	-1.83352300	-2.02429100	-2.59740700
H	-2.33648100	-2.98786500	-2.46472700
H	-0.75597200	-2.14897200	-2.45092700
H	-2.01980200	-1.66037100	-3.60905100
C	-4.33838000	0.00202700	-0.01218900
H	-4.80626600	-0.91049800	0.36494000
H	-4.45938100	0.04311000	-1.09411400
C	-3.30823800	0.73283100	2.62846600
H	-2.82345000	0.60912900	3.59814000
H	-4.36373100	0.45513900	2.71739500
C	2.13485700	-2.87160100	0.72797700
H	2.31077700	-3.33345800	1.70328800
H	2.86943900	-3.26064800	0.01551000
H	1.13323100	-3.14742200	0.38713000
H	-3.22797200	1.77818300	2.31388400
H	-4.84314400	0.86439100	0.42869500
H	3.94087400	0.07395900	-1.59431400

7
Total electronic energy: -361.241685802 Hartree
Number of imaginary frequencies: 0

O	0.09004200	-0.92000400	0.36785400
O	2.27727400	-0.67302700	-0.20873000
N	0.78095800	1.11514100	-0.15413800
C	-0.54600400	1.31042900	0.20695900
H	-1.10599300	2.15664000	-0.16674400
C	-1.10051500	-0.06135700	0.47899300
H	-1.45409100	-0.17503700	1.51138100
C	1.16996300	-0.19281600	-0.02228500
C	-2.17787200	-0.54203000	-0.48902200
H	-2.46056700	-1.57400700	-0.26125600
H	-1.82777400	-0.48359200	-1.52382800
H	-3.06503500	0.09050800	-0.38280600
H	1.43479500	1.83838700	-0.41864100

References

(1) Arthurs, C. L.; Morris, G. A.; Piacenti, M.; Pritchard, R. G.; Stratford, I. J.; Tatic, T.; Whitehead, R. C.; Williams, K. F.; Wind, N. S. The Synthesis of 2-Oxyalkyl-Cyclohex-2-Enones, Related to the Bioactive Natural Products COTC and Antheminone A, Which Possess Anti-Tumour Properties. *Tetrahedron* **2010**, *66* (46), 9049–9060. https://doi.org/10.1016/j.tet.2010.08.072.

(2) Horn, A.; Kazmaier, U. Purified *m*CPBA, a Useful Reagent for the Oxidation of Aldehydes. *Eur. J. Org. Chem.* **2018**, *2018* (20–21), 2531–2536. https://doi.org/10.1002/ejoc.201701645.

(3) Vojáčková, P.; Michalska, L.; Nečas, M.; Shcherbakov, D.; Böttger, E. C.; Šponer, J.; Šponer, J. E.; Švenda, J. Stereocontrolled Synthesis of (−)-Bactobolin A. *J. Am. Chem. Soc.* **2020**, *142* (16), 7306–7311. https://doi.org/10.1021/jacs.0c01554.

(4) Jaiswal, R.; Dickman, M. H.; Kuhnert, N. First Diastereoselective Synthesis of Methyl Caffeoyl- and Feruloyl-Muco-Quinates. *Org. Biomol. Chem.* **2012**, *10* (27), 5266–5277. https://doi.org/10.1039/c2ob25124h.

(5) Rigaku Oxford Diffraction. CrysAlisPro Software System, 2025.

(6) Sheldrick, G. M. *SHELXT* – Integrated Space-Group and Crystal-Structure Determination. *Acta Crystallogr., Sect. A: Found. Adv.* **2015**, *71* (1), 3–8. https://doi.org/10.1107/S2053273314026370.

(7) Sheldrick, G. M. Crystal Structure Refinement with *SHELXL*. *Acta Crystallogr., Sect. C: Struct. Chem.* **2015**, *71* (1), 3–8. https://doi.org/10.1107/S2053229614024218.

(8) Becke, A. D. Density-Functional Thermochemistry. III. The Role of Exact Exchange. *J. Chem. Phys.* **1993**, *98* (7), 5648–5652. https://doi.org/10.1063/1.464913.

(9) Lee, C.; Yang, W.; Parr, R. G. Development of the Colle-Salvetti Correlation-Energy Formula into a Functional of the Electron Density. *Phys. Rev. B* **1988**, *37* (2), 785–789. https://doi.org/10.1103/PhysRevB.37.785.

(10) Frisch, M. J.; Trucks, G. W.; Schlegel, H. B.; Scuseria, G. E.; Robb, M. A.; Cheeseman, J. R.; Scalmani, G.; Barone, V.; Petersson, G. A.; Nakatsuji, H.; Li, X.; Caricato, M.; Marenich, A.; Bloino, J.; Janesko, B. G.; Gomperts, R.; Mennucci, B.; Hratchian, H. P.; Ortiz, J. V.; Izmaylov, A. F.; Sonnenberg, J. L.; Williams-Young, D.; Ding, F.; Lipparini, F.; Egidi, F.; Goings, J.; Peng, B.; Petrone, A.; Henderson, T.; Ranasinghe, D.; Zakrzewski, V. G.; Gao, J.; Rega, N.; Zheng, G.; Liang, W.; Hada, M.; Ehara, M.; Toyota, K.; Fukuda, R.; Hasegawa, J.; Ishida, M.; Nakajima, T.; Honda, Y.; Kitao, O.; Nakai, H.; Vreven, T.; Throssell, K.; Montgomery, Jr., J. A.; Peralta, J. E.; Ogliaro, F.; Bearpark, M.; Heyd, J. J.; Brothers, E.; Kudin, K. N.; Staroverov, V. N.; Keith, T.; Kobayashi, R.; Normand, J.; Raghavachari, K.; Rendell, A.; Burant, J. C.; Iyengar, S. S.; Tomasi, J.; Cossi, M.; Millam, J. M.; Klene, M.; Adamo, C.; Cammi, R.; Ochterski, J. W.; Martin, R. L.; Morokuma, K.; Farkas, O.; Foresman, J. B.; Fox, D. J. Gaussian 09, Revision A.02, 2016.

(11) Tomasi, J.; Mennucci, B.; Cammi, R. Quantum Mechanical Continuum Solvation Models. *Chem. Rev.* **2005**, *105* (8), 2999–3094. https://doi.org/10.1021/cr9904009.

NMR Spectra

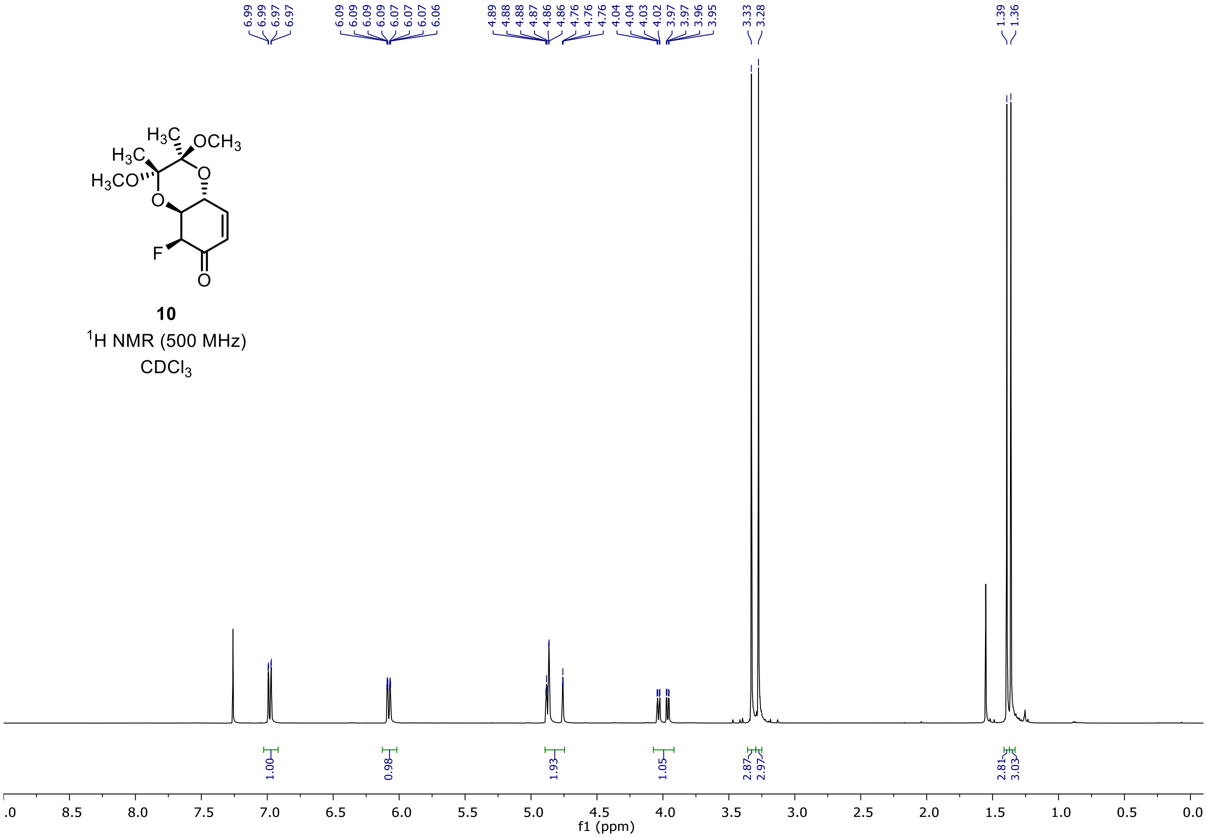

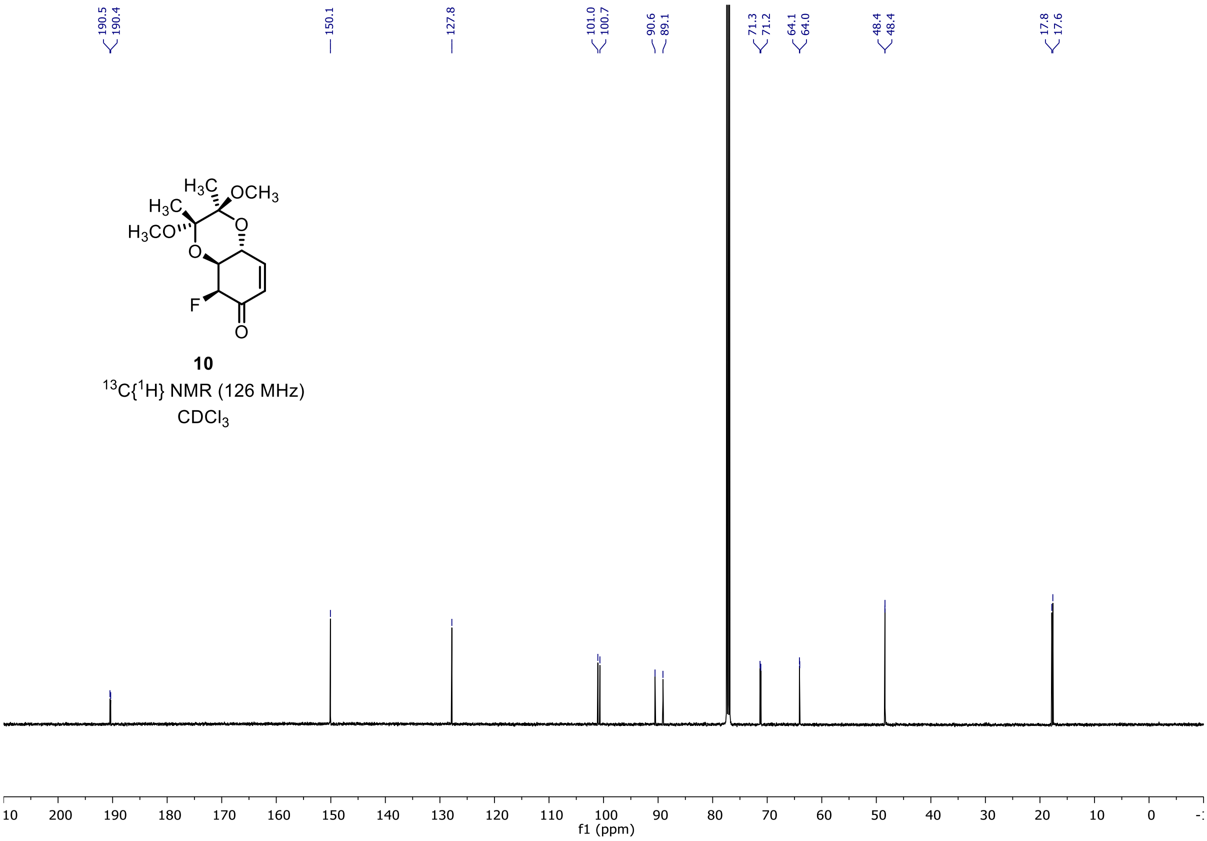

10
^{19}F NMR (471 MHz)
$CDCl_3$

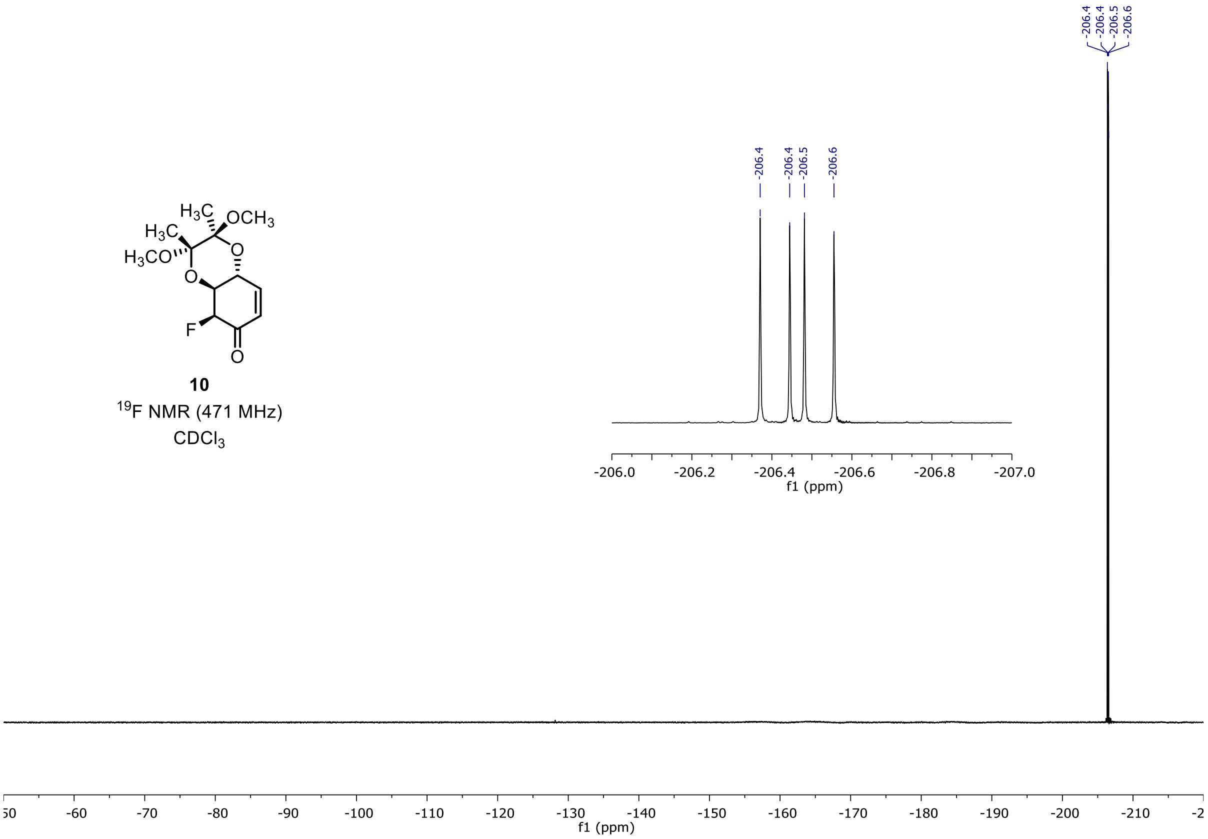

12
^{1}H NMR (500 MHz)
$CDCl_3$

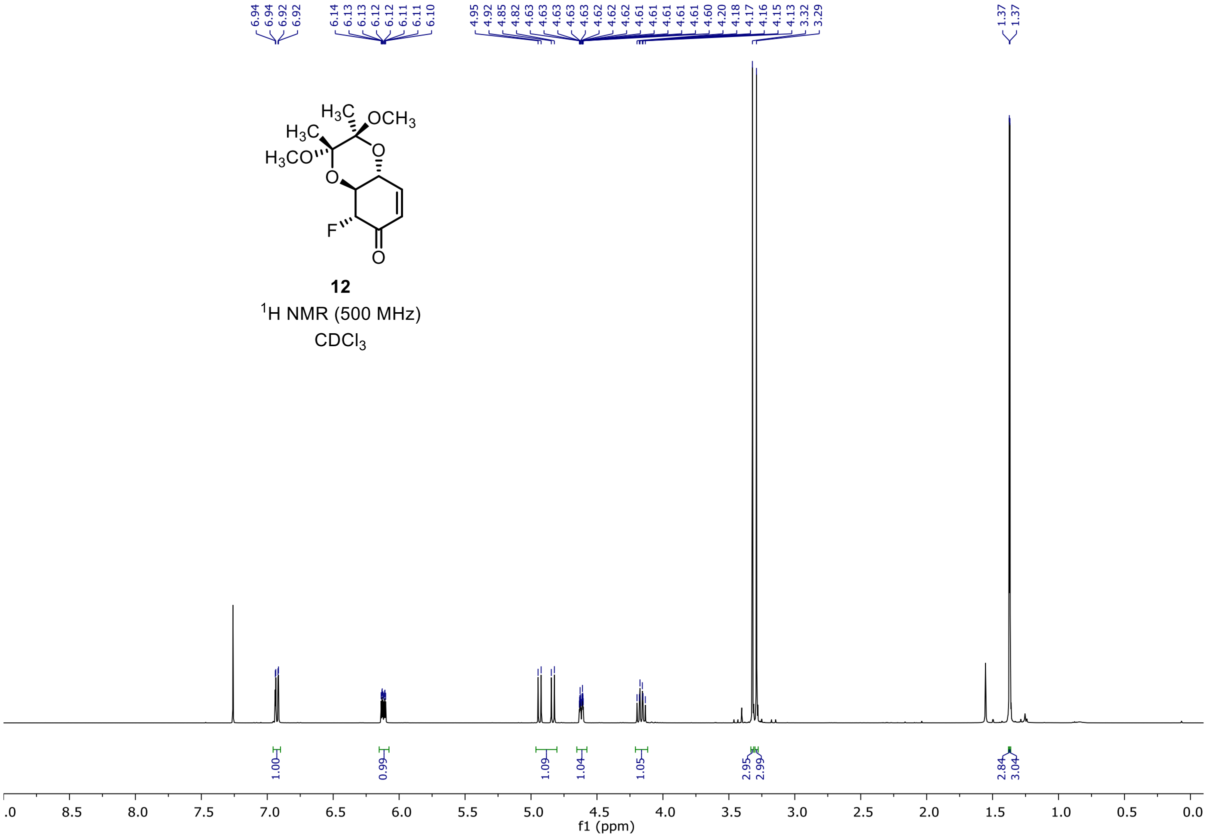

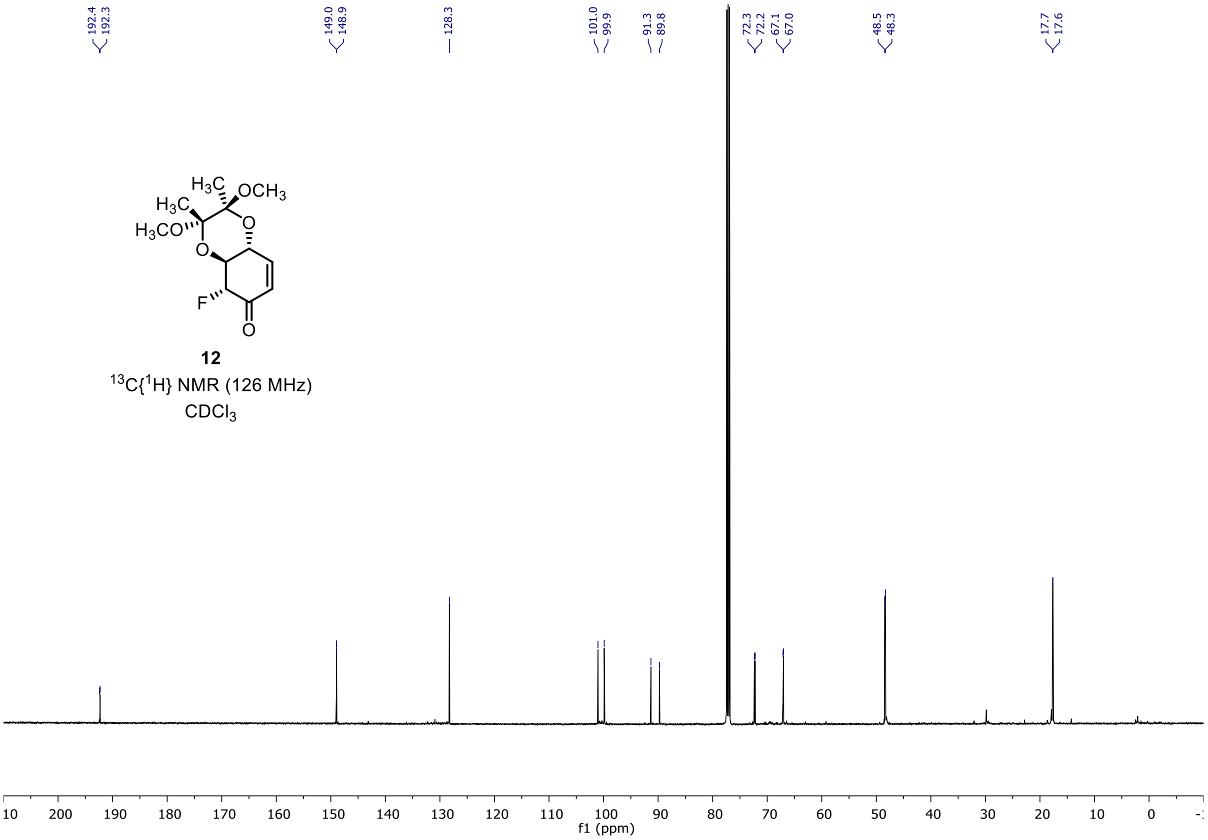
192.4
192.3
149.0
148.9
128.3
101.0
99.9
91.3
89.8
72.3
72.2
67.1
67.0
48.5
48.3
17.7
17.6
H3C
OCH3
H3C
H3CO
O
O
F
O
12
13C{1H} NMR (126 MHz)
CDCl3
f1 (ppm)

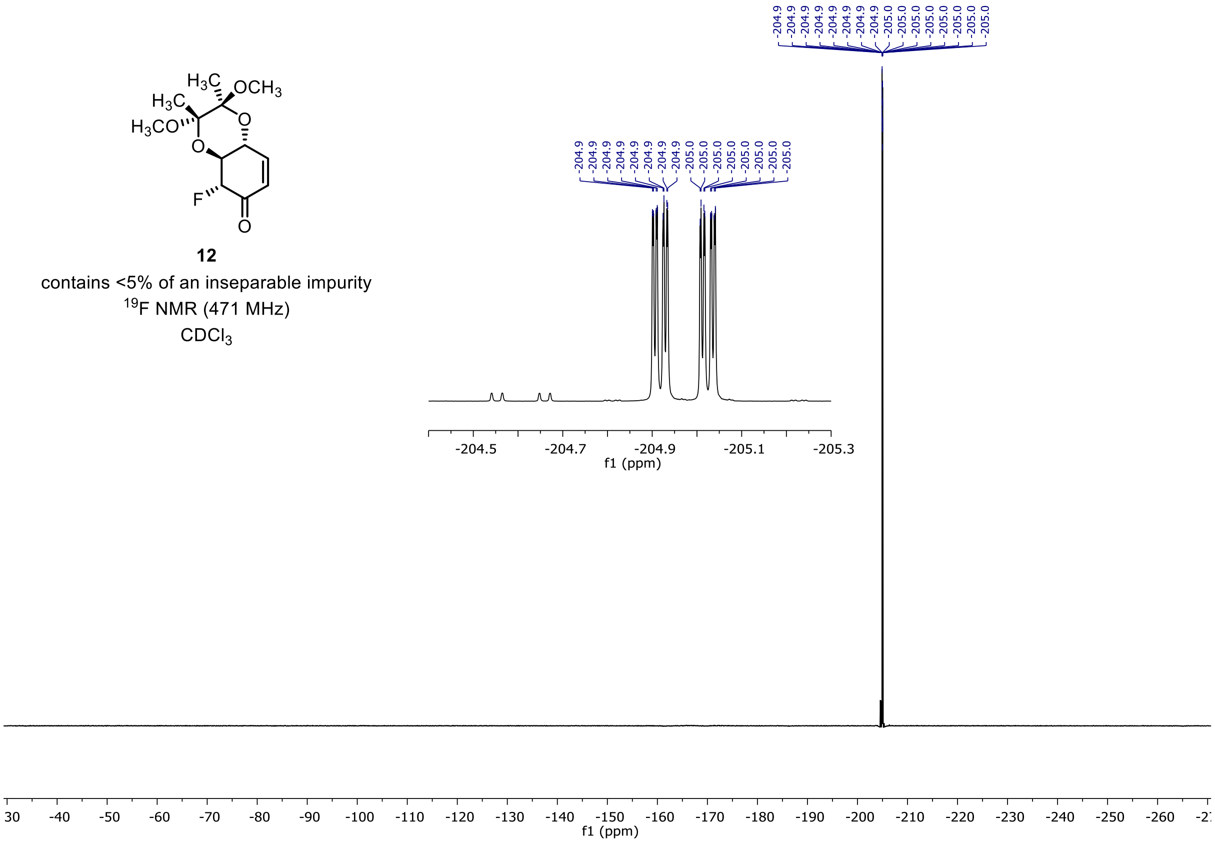
H3C
OCH3
H3C
H3CO
O
O
F
O
12
contains <5% of an inseparable impurity
19F NMR (471 MHz)
CDCl3
-204.5
-204.7
-204.9
-205.1
-205.3
f1 (ppm)

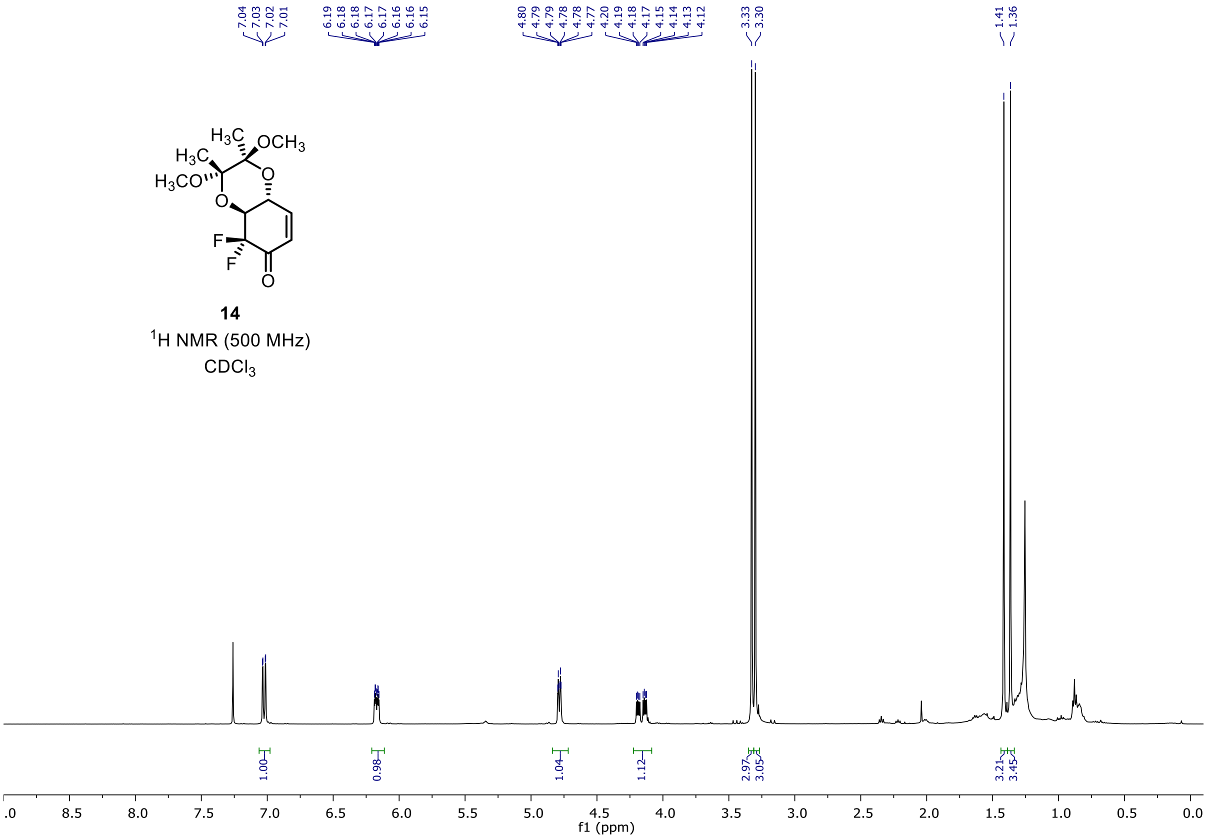
14
^1H NMR (500 MHz)
$CDCl_3$
f1 (ppm)

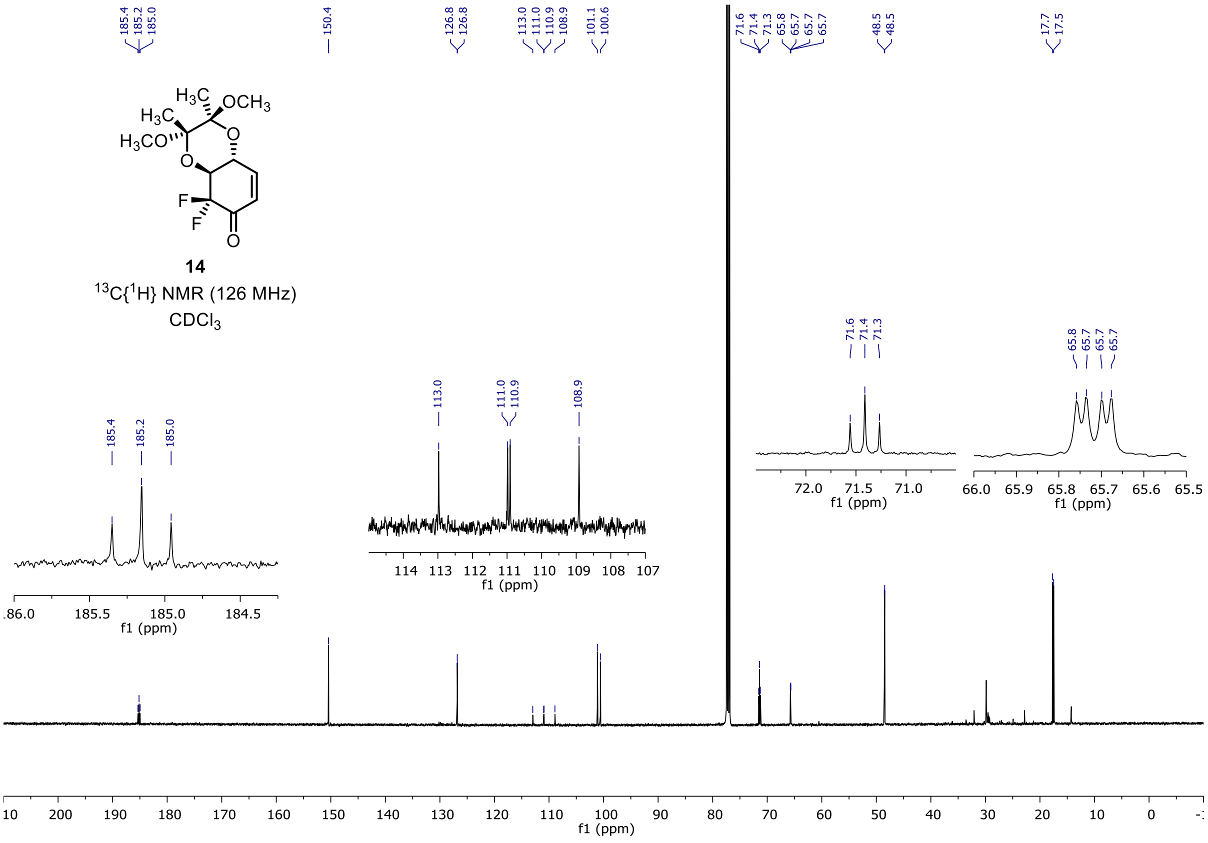
14
$^{13}C\{^1H\}$ NMR (126 MHz)
$CDCl_3$
f1 (ppm)

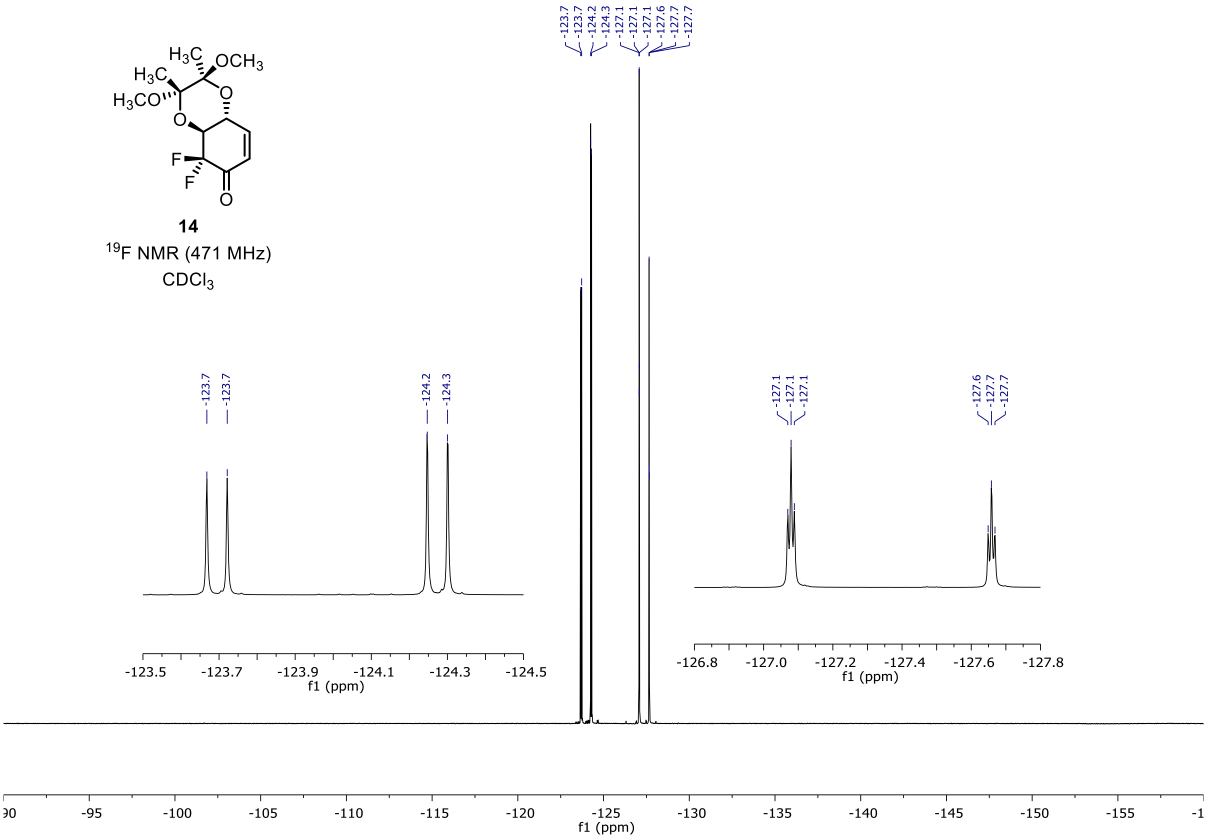
14
^{19}F NMR (471 MHz)
$CDCl_3$
f1 (ppm)

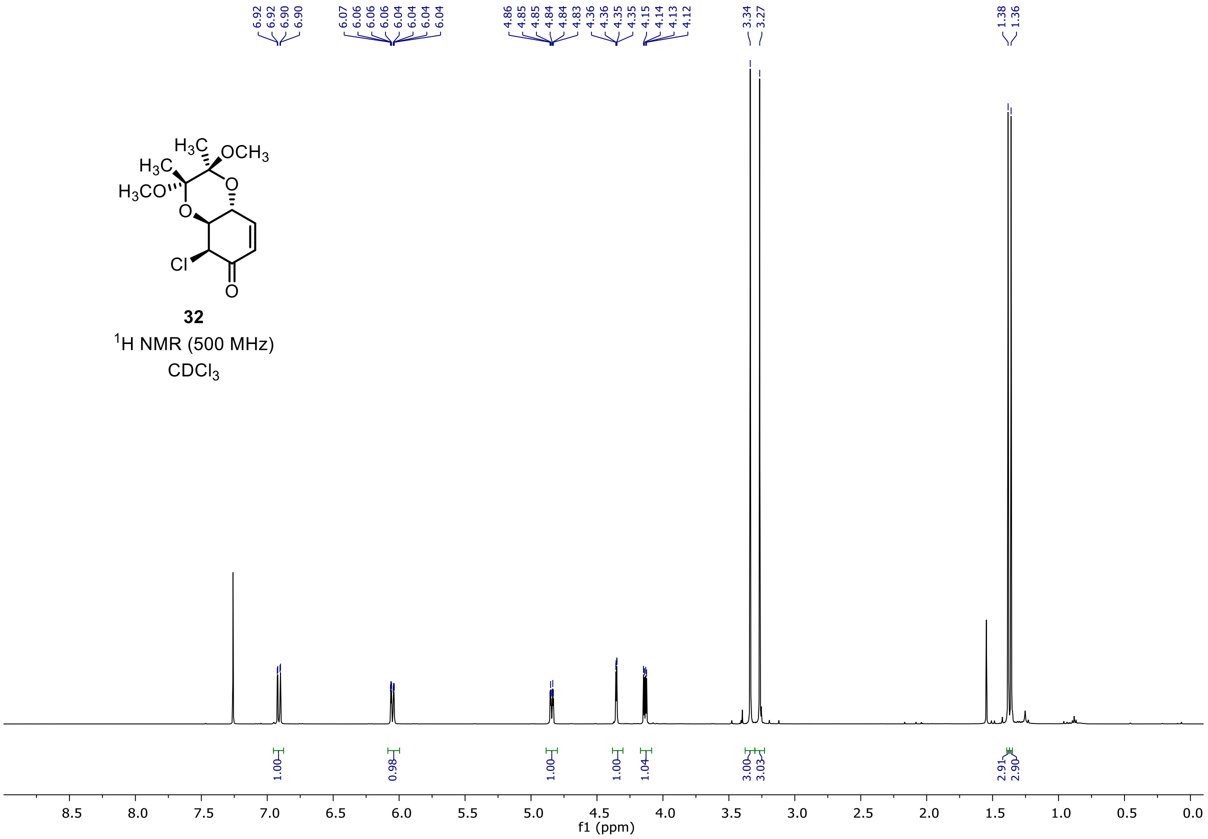
32
^{1}H NMR (500 MHz)
$CDCl_3$
f1 (ppm)

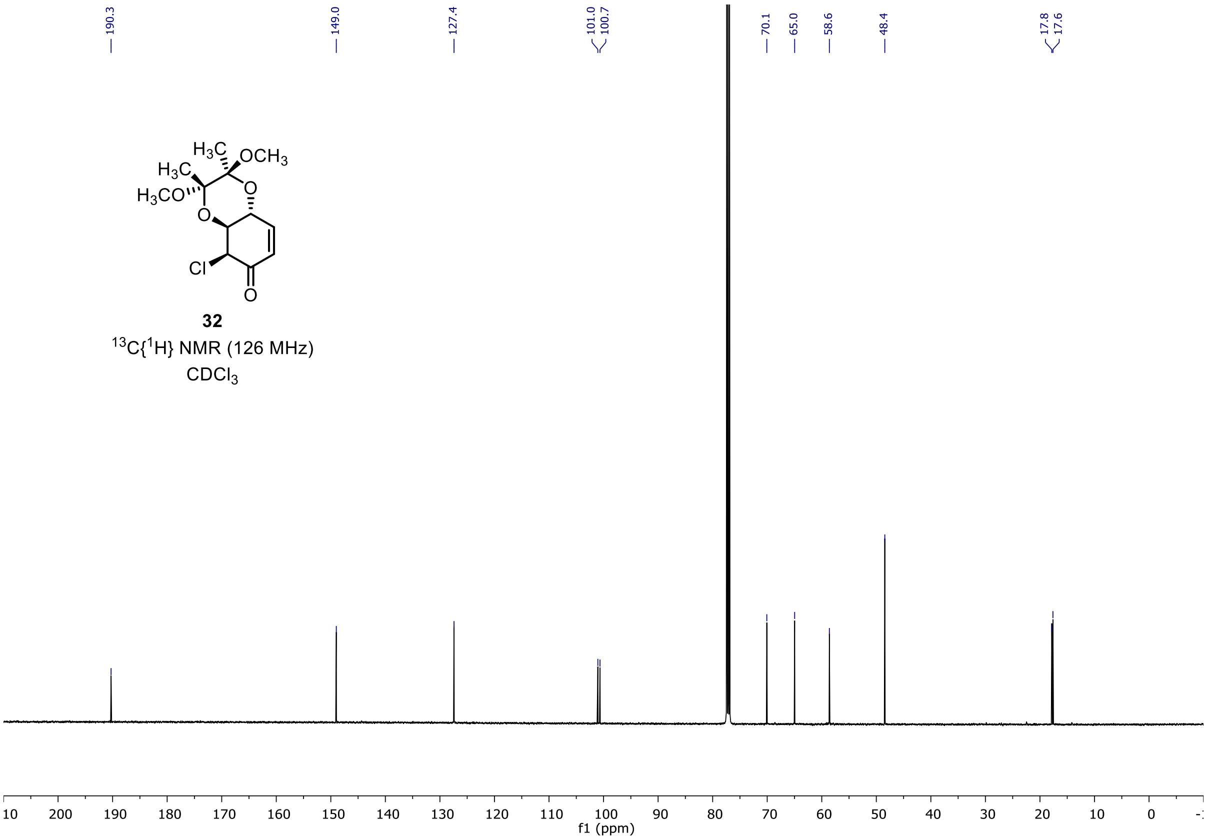

32

$^{13}C\{^1H\}$ NMR (126 MHz)

$CDCl_3$

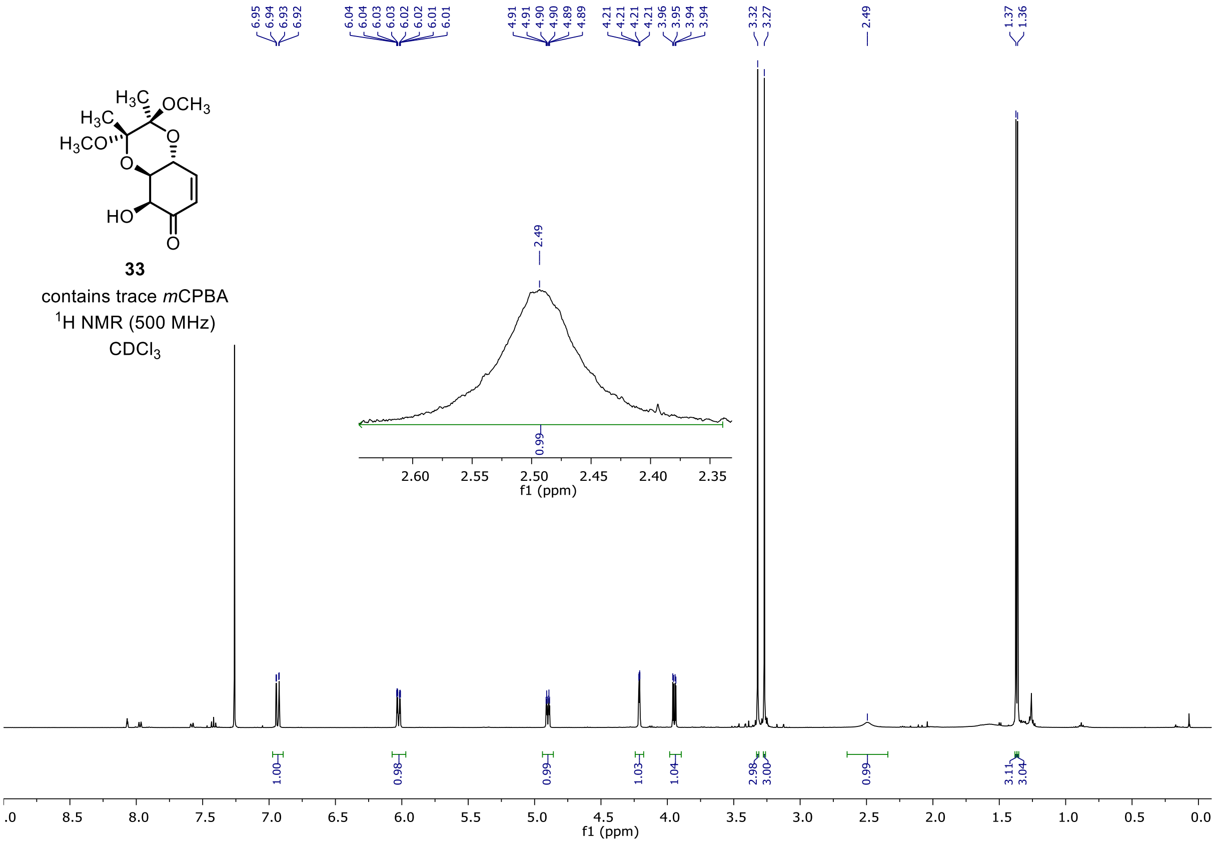

33

contains trace *m*CPBA

^1H NMR (500 MHz)

$CDCl_3$

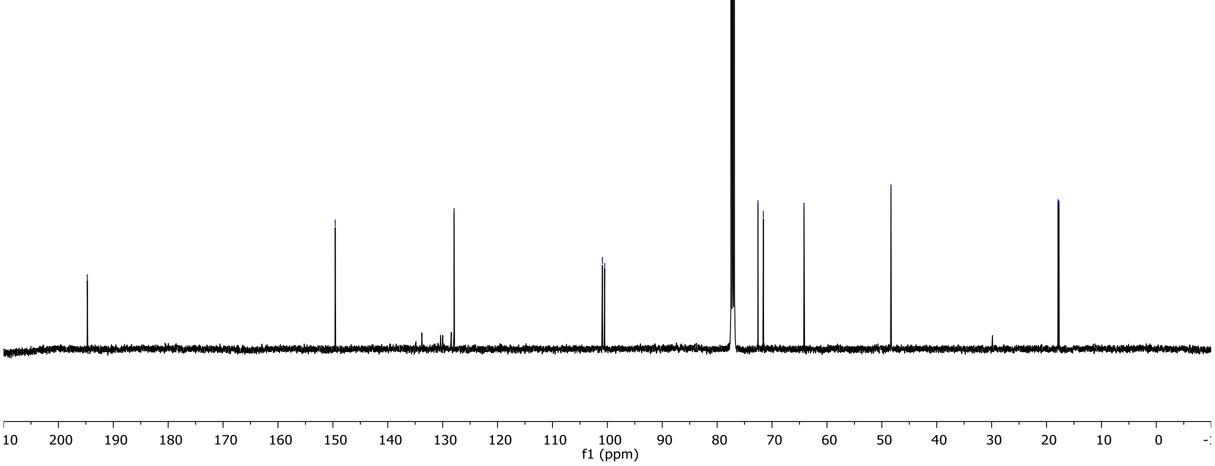

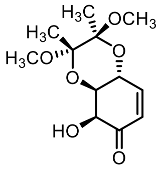

33

contains trace *m*CPBA

$^{13}C\{^{1}H\}$ NMR (126 MHz)

$CDCl_3$

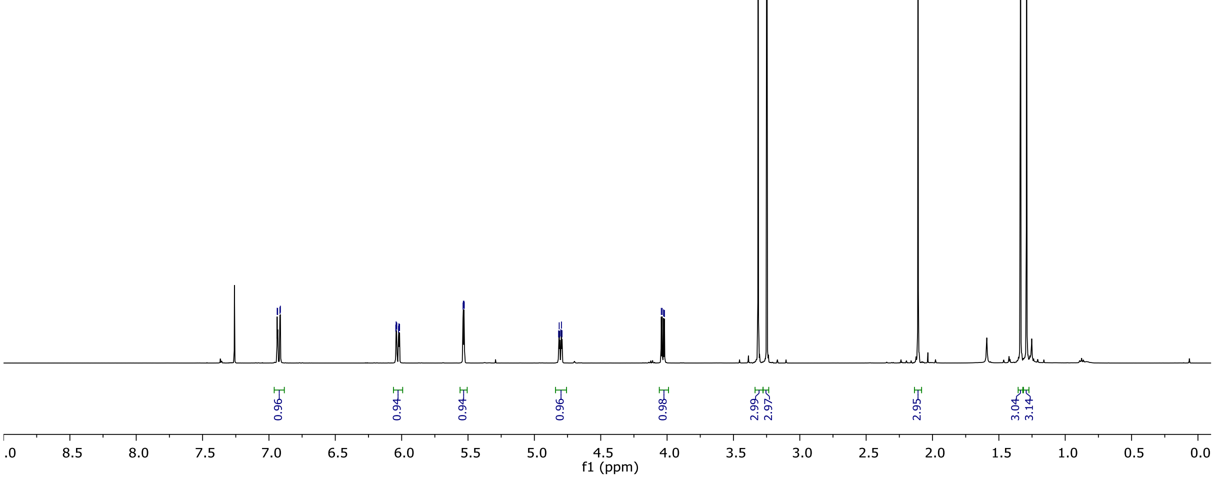

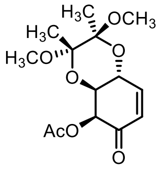

34

^{1}H NMR (500 MHz)

$CDCl_3$

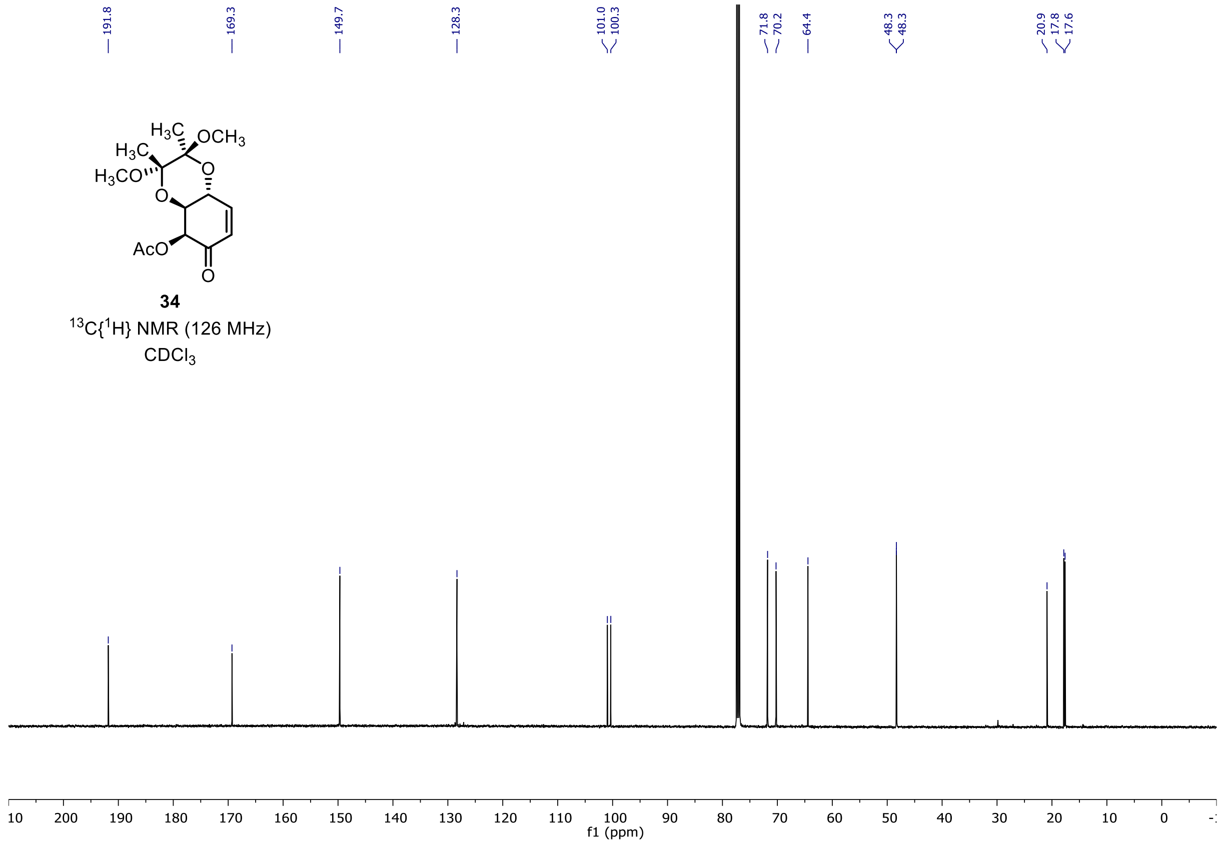

34

$^{13}C\{^1H\}$ NMR (126 MHz)

$CDCl_3$

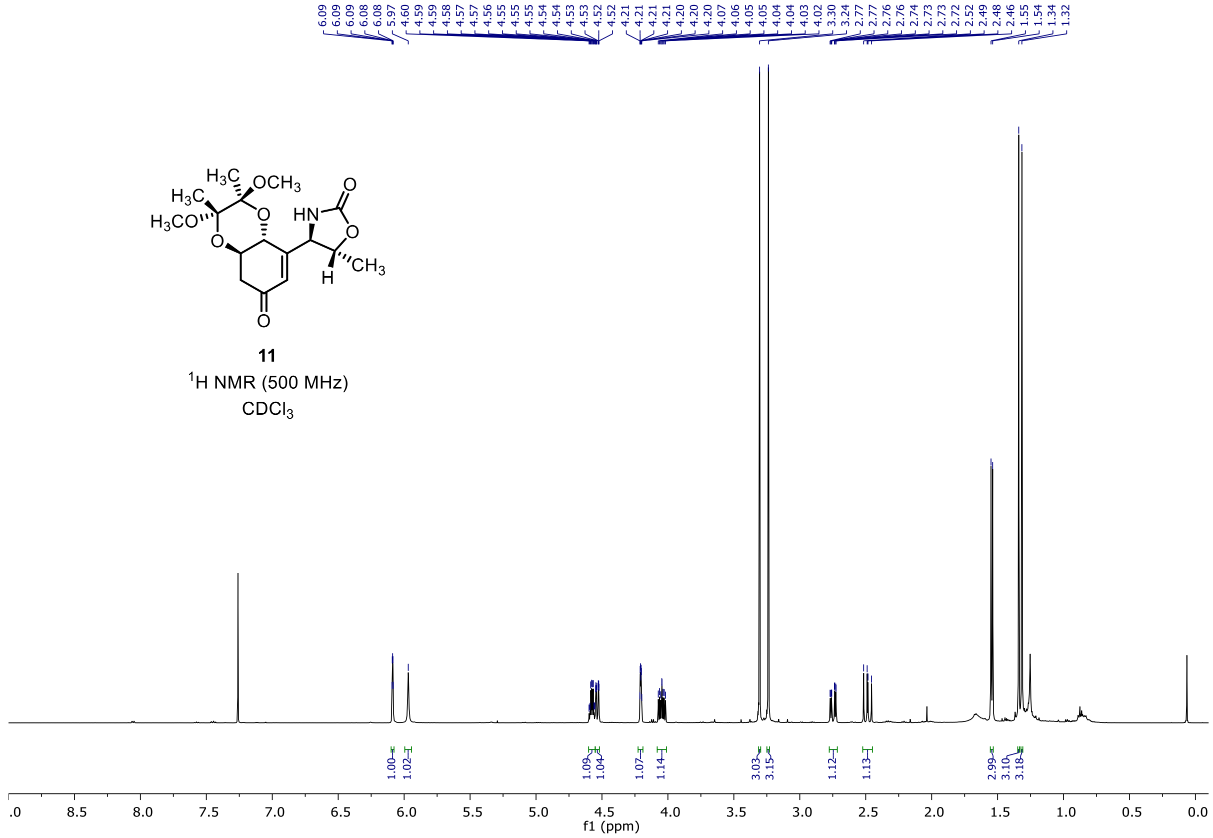

11

^1H NMR (500 MHz)

$CDCl_3$

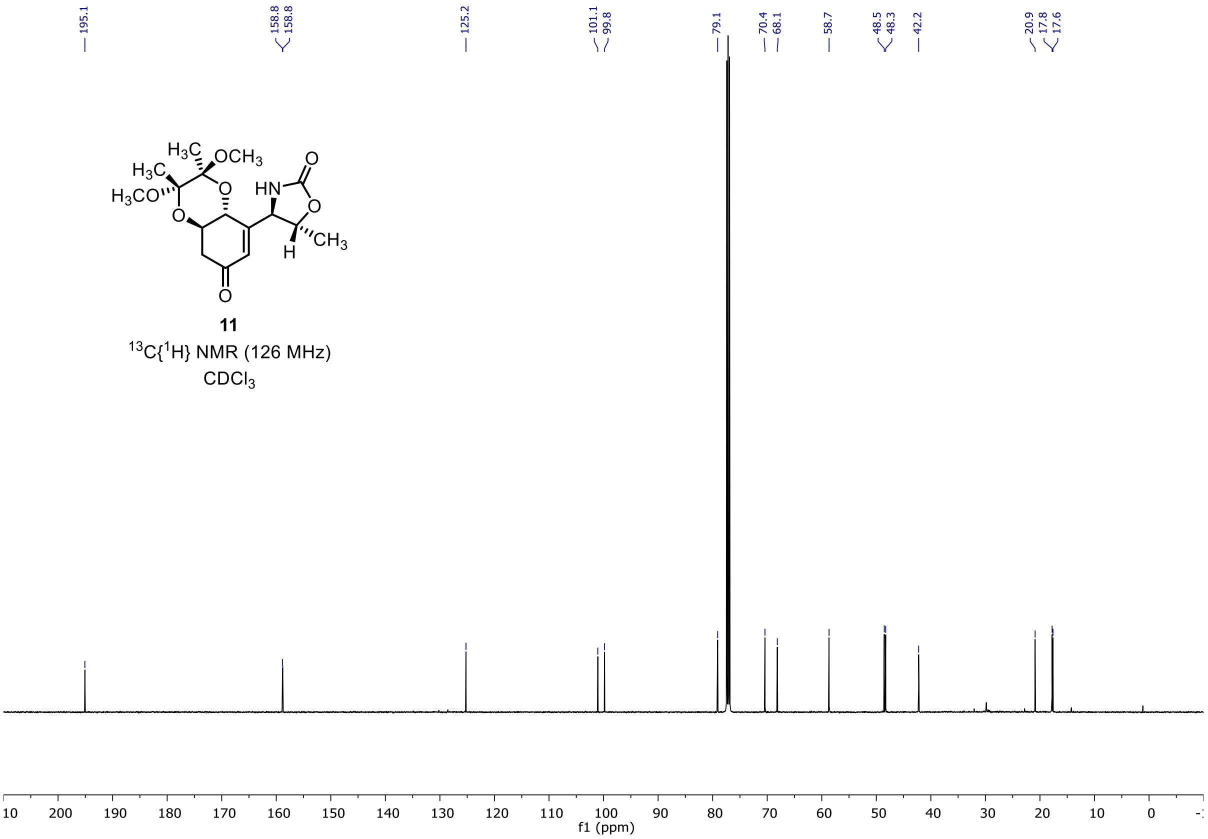
195.1
158.8
158.8
125.2
101.1
99.8
79.1
70.4
68.1
58.7
48.5
48.3
42.2
20.9
17.8
17.6
11
$^{13}C\{^{1}H\}$ NMR (126 MHz)
$CDCl_3$
f1 (ppm)

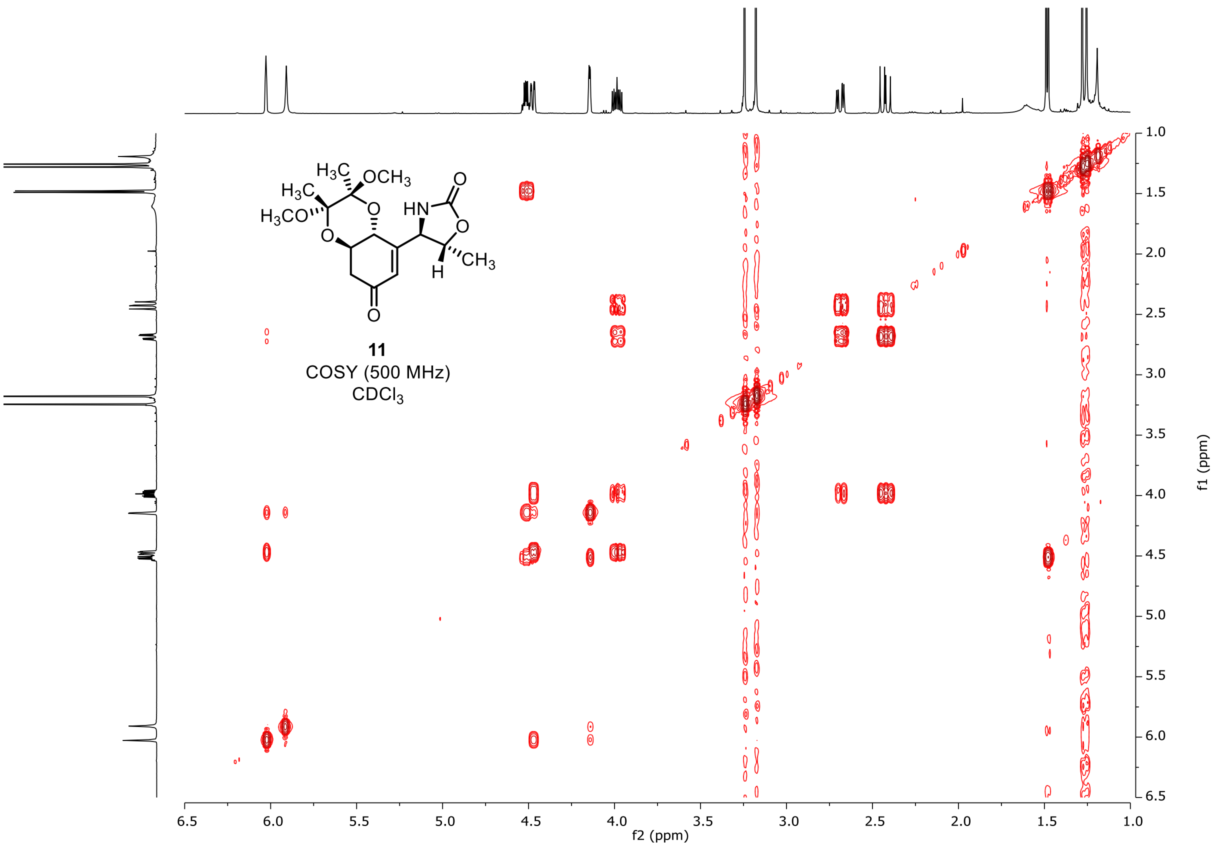
11
COSY (500 MHz)
$CDCl_3$
f2 (ppm)
f1 (ppm)

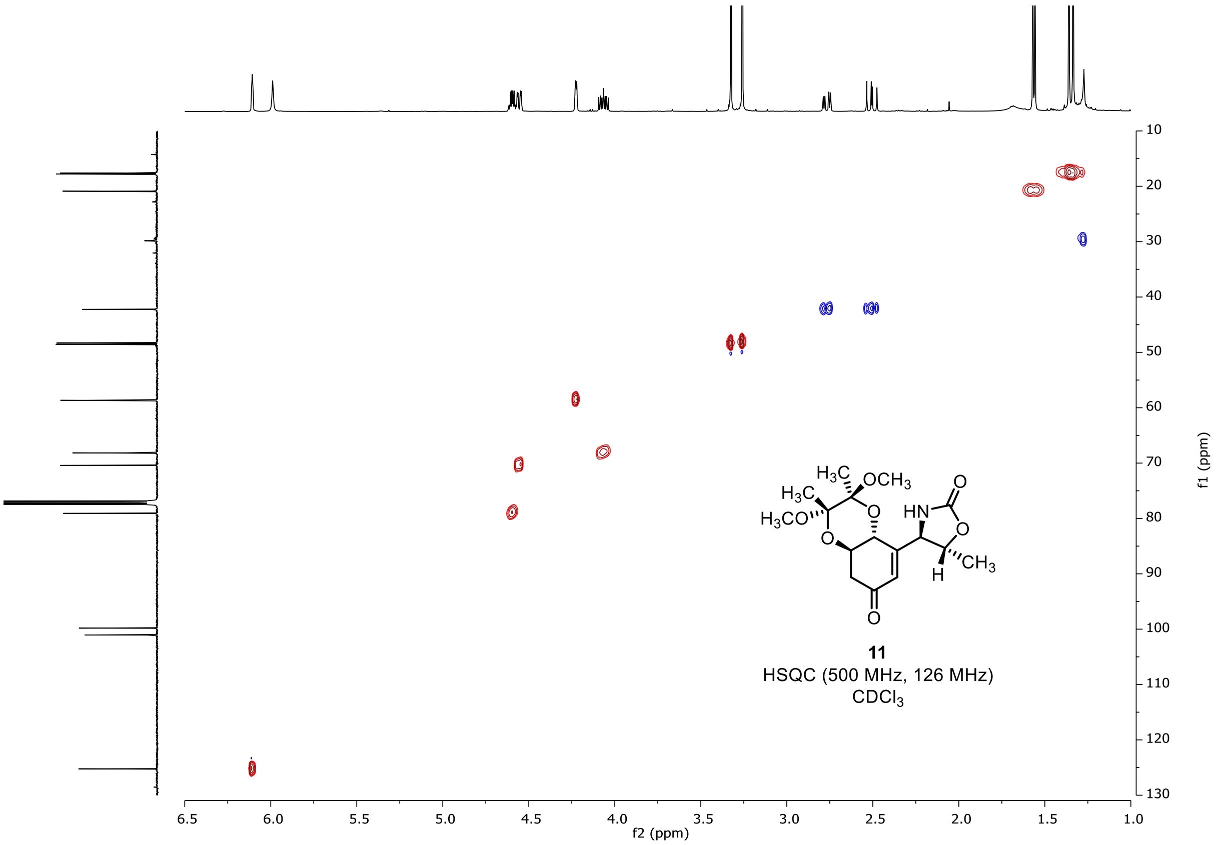

11
HSQC (500 MHz, 126 MHz)
CDCl3
f2 (ppm)
f1 (ppm)

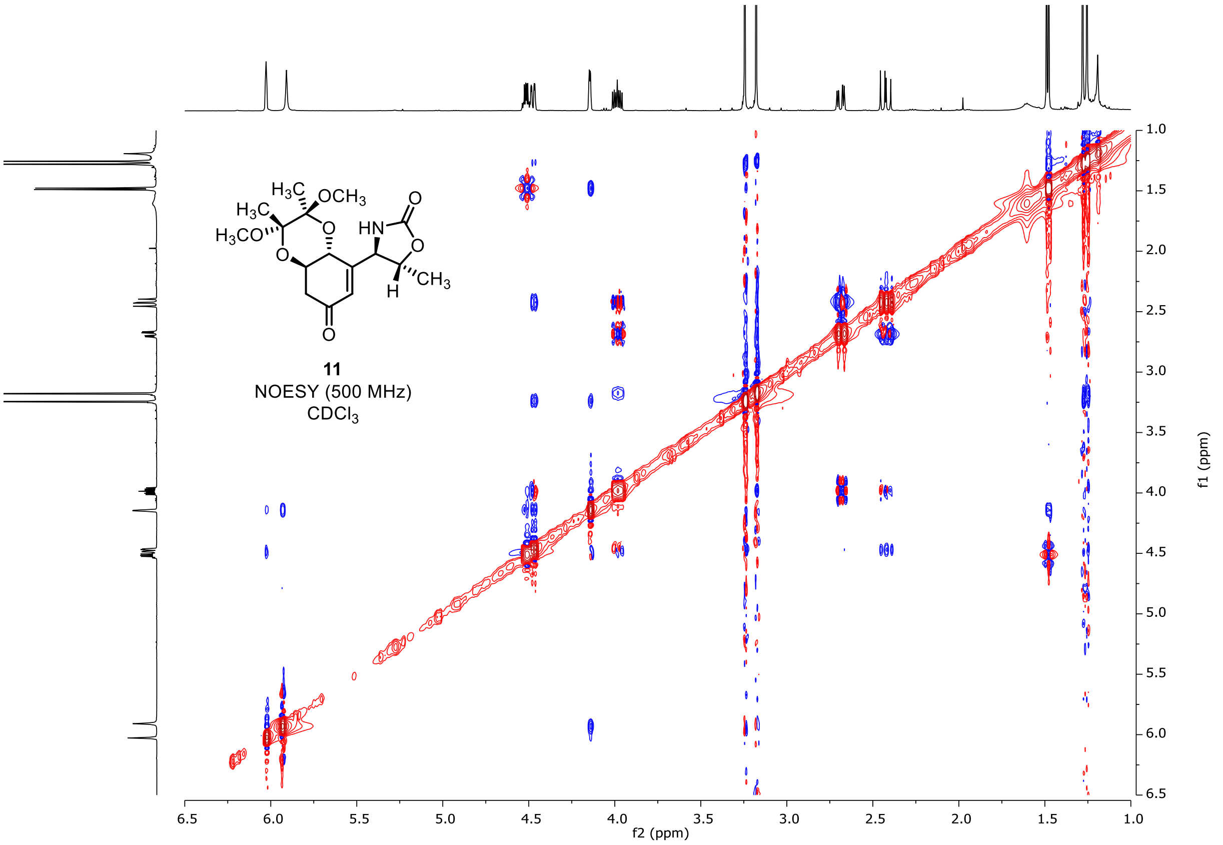

11
NOESY (500 MHz)
CDCl3
f2 (ppm)
f1 (ppm)

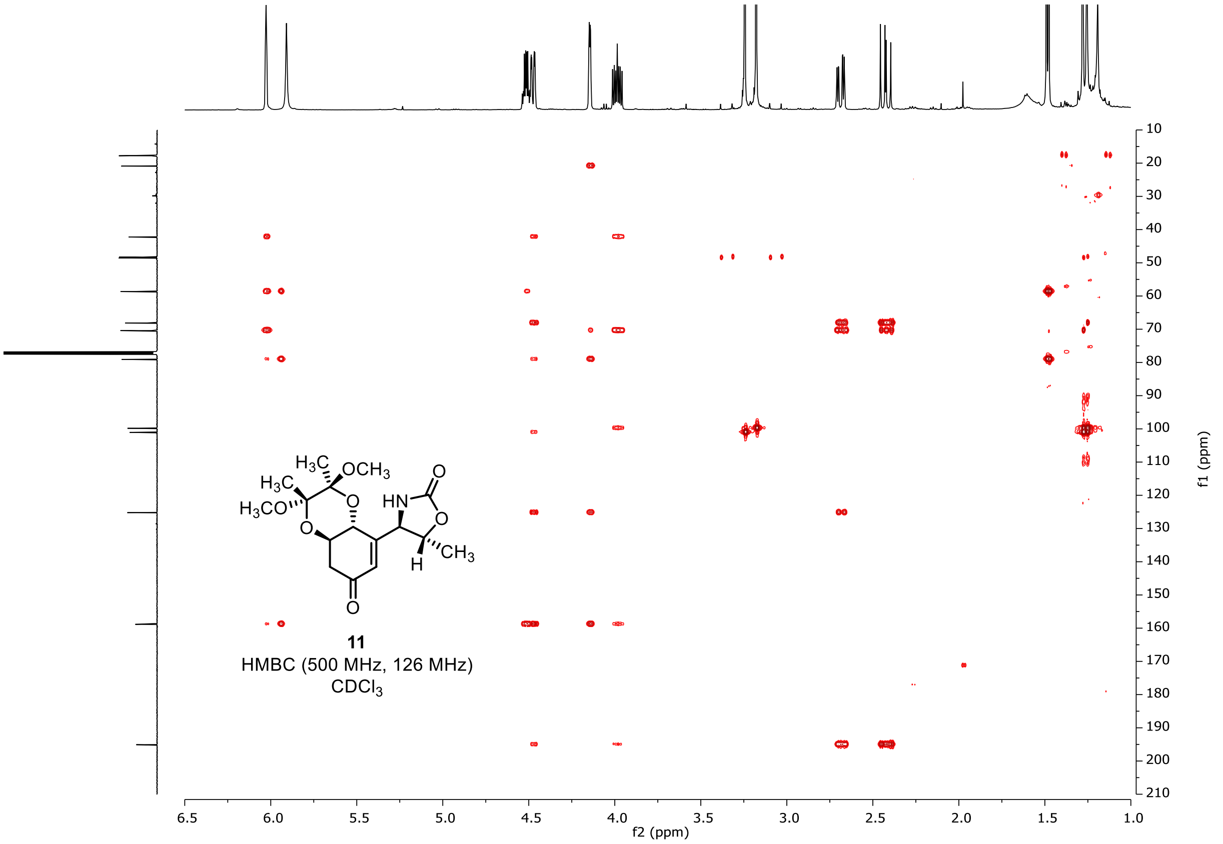

11
HMBC (500 MHz, 126 MHz)
$CDCl_3$

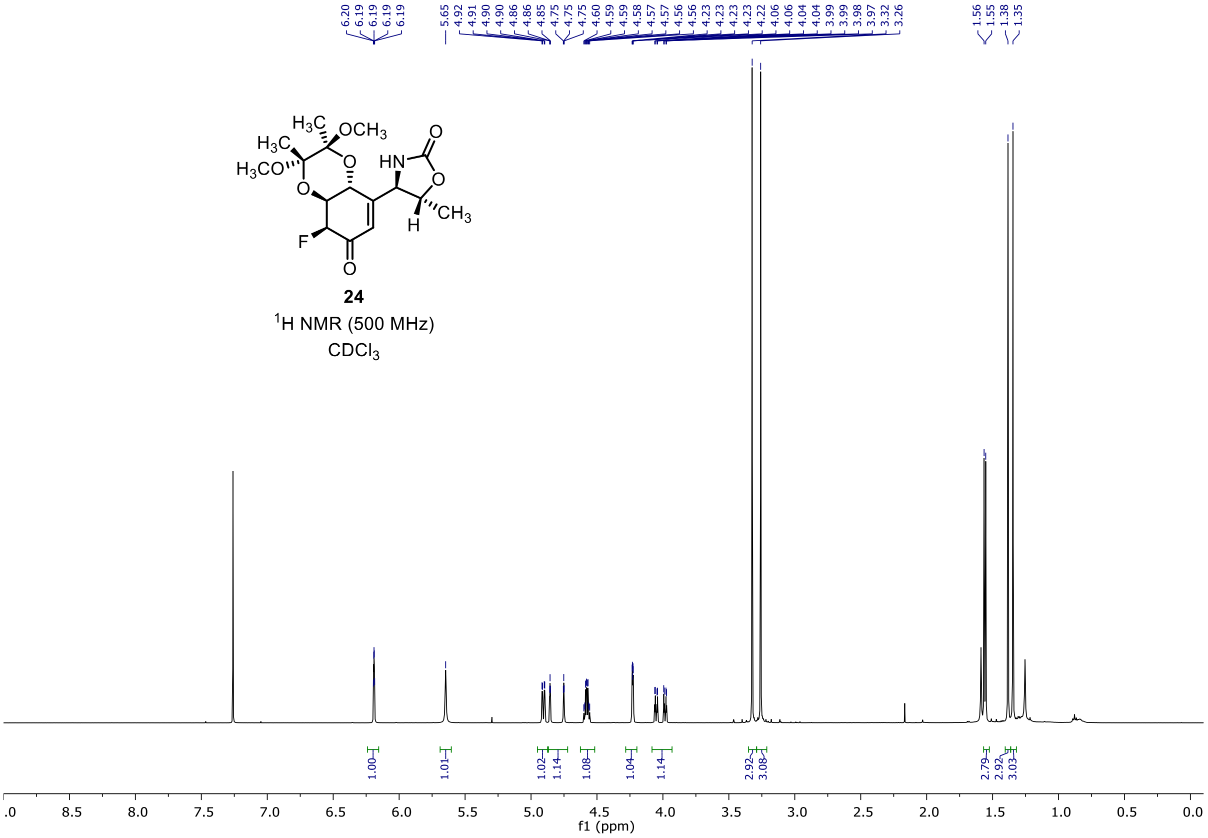

24
^{1}H NMR (500 MHz)
$CDCl_3$

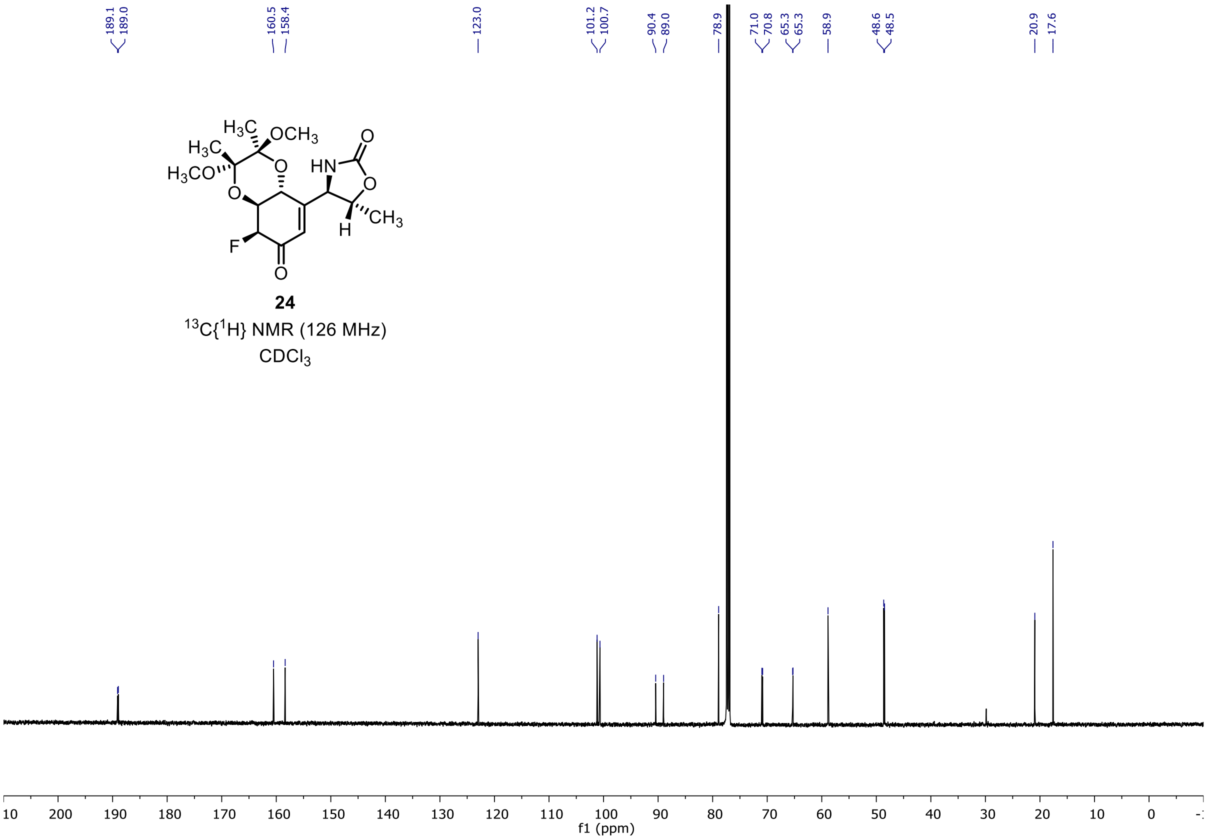
189.1
189.0
160.5
158.4
123.0
101.2
100.7
90.4
89.0
78.9
71.0
70.8
65.3
65.3
58.9
48.6
48.5
20.9
17.6
24
$^{13}C\{^1H\}$ NMR (126 MHz)
$CDCl_3$
f1 (ppm)

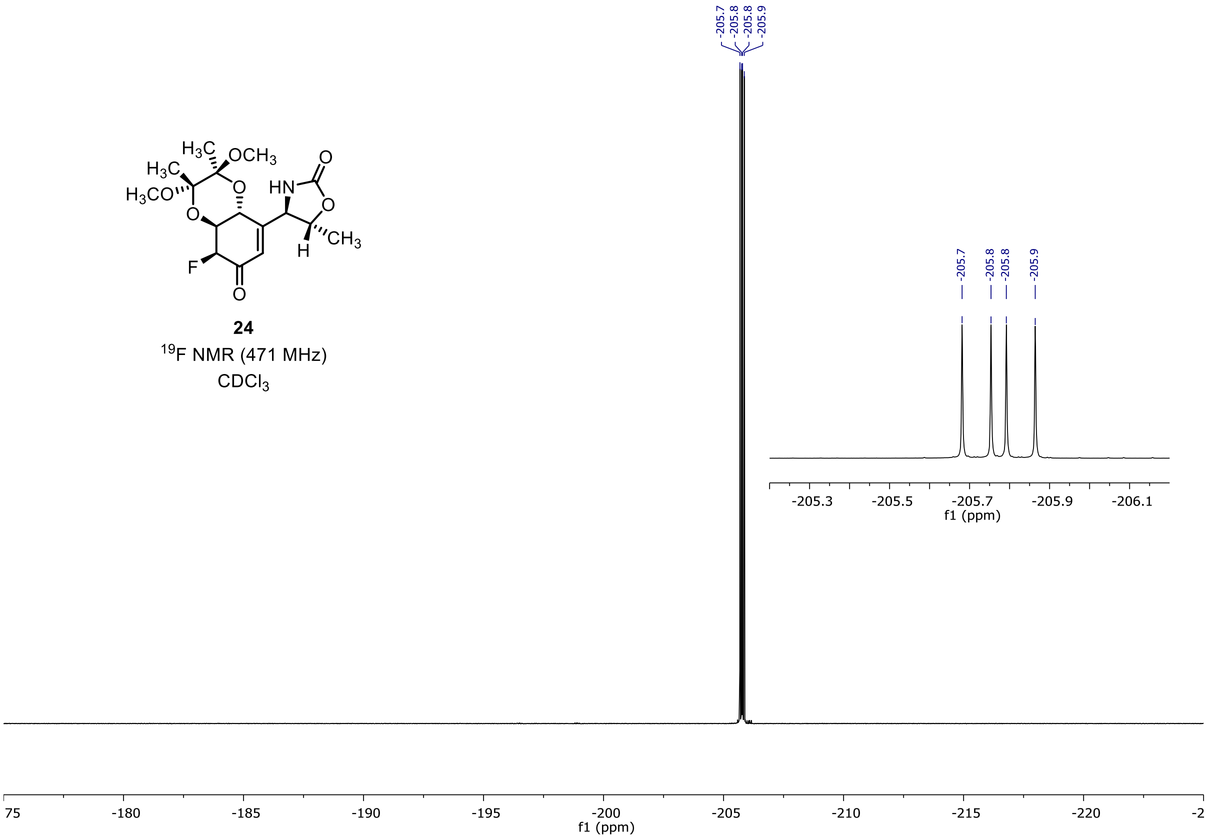
-205.7
-205.8
-205.8
-205.9
24
^{19}F NMR (471 MHz)
$CDCl_3$
f1 (ppm)

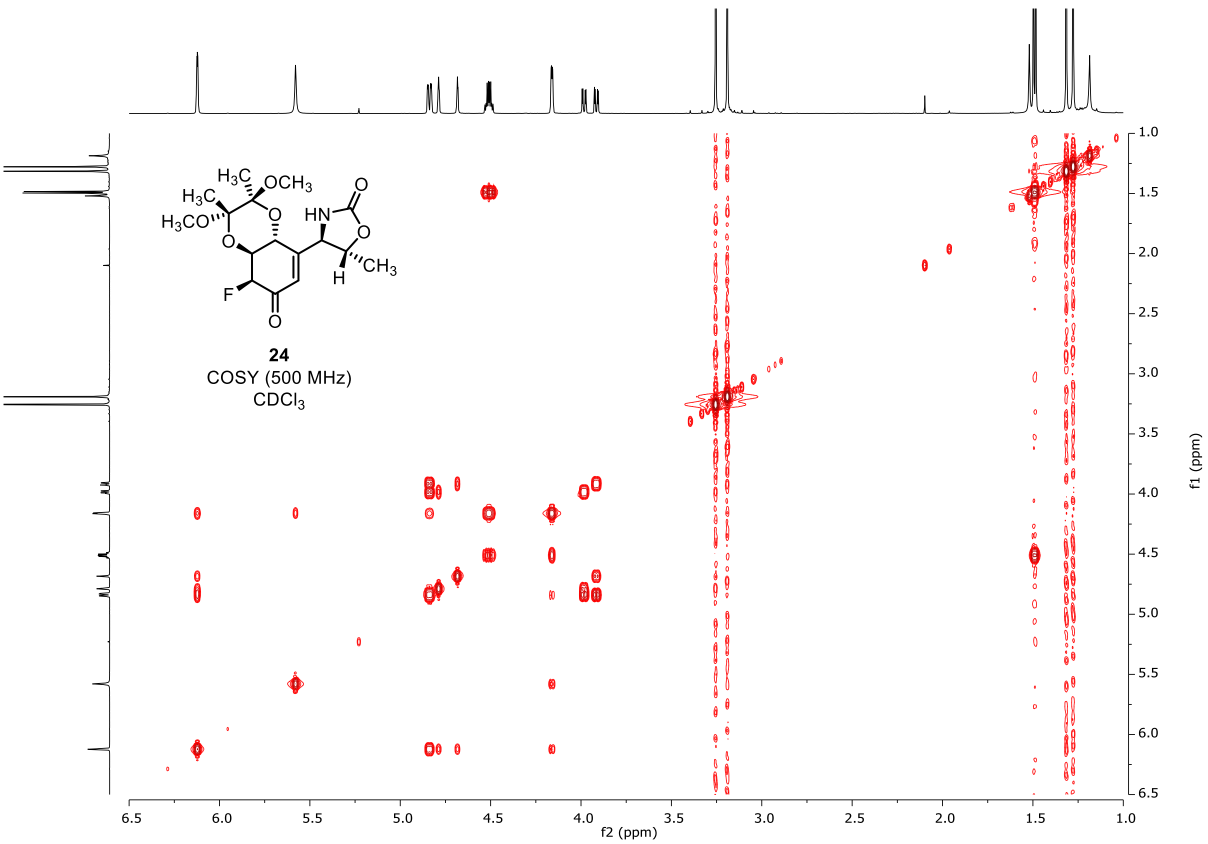

H3C
OCH3
H3C
H3CO
O
HN
O
O
O
CH3
H
F
O
24
COSY (500 MHz)
CDCl3
f2 (ppm)
f1 (ppm)

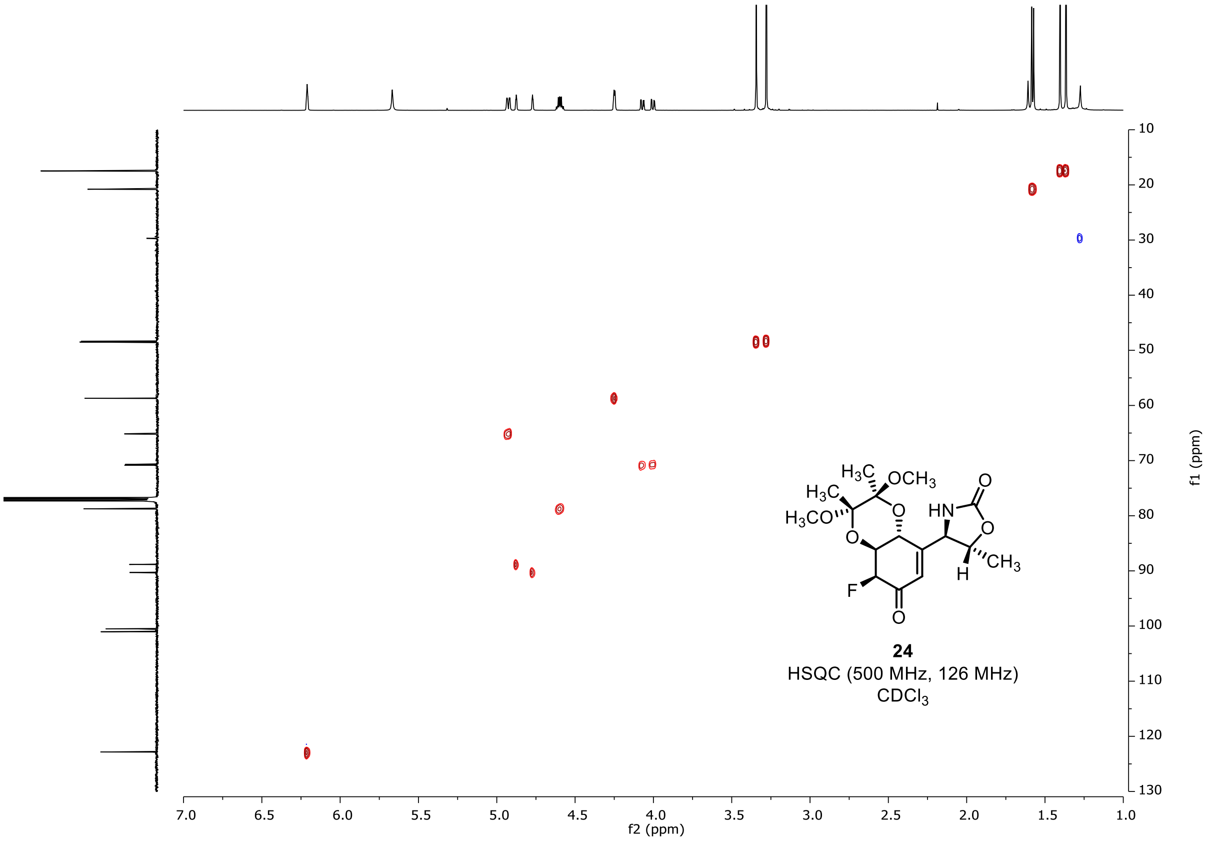

H3C
OCH3
H3C
H3CO
O
HN
O
O
O
CH3
H
F
O
24
HSQC (500 MHz, 126 MHz)
CDCl3
f2 (ppm)
f1 (ppm)

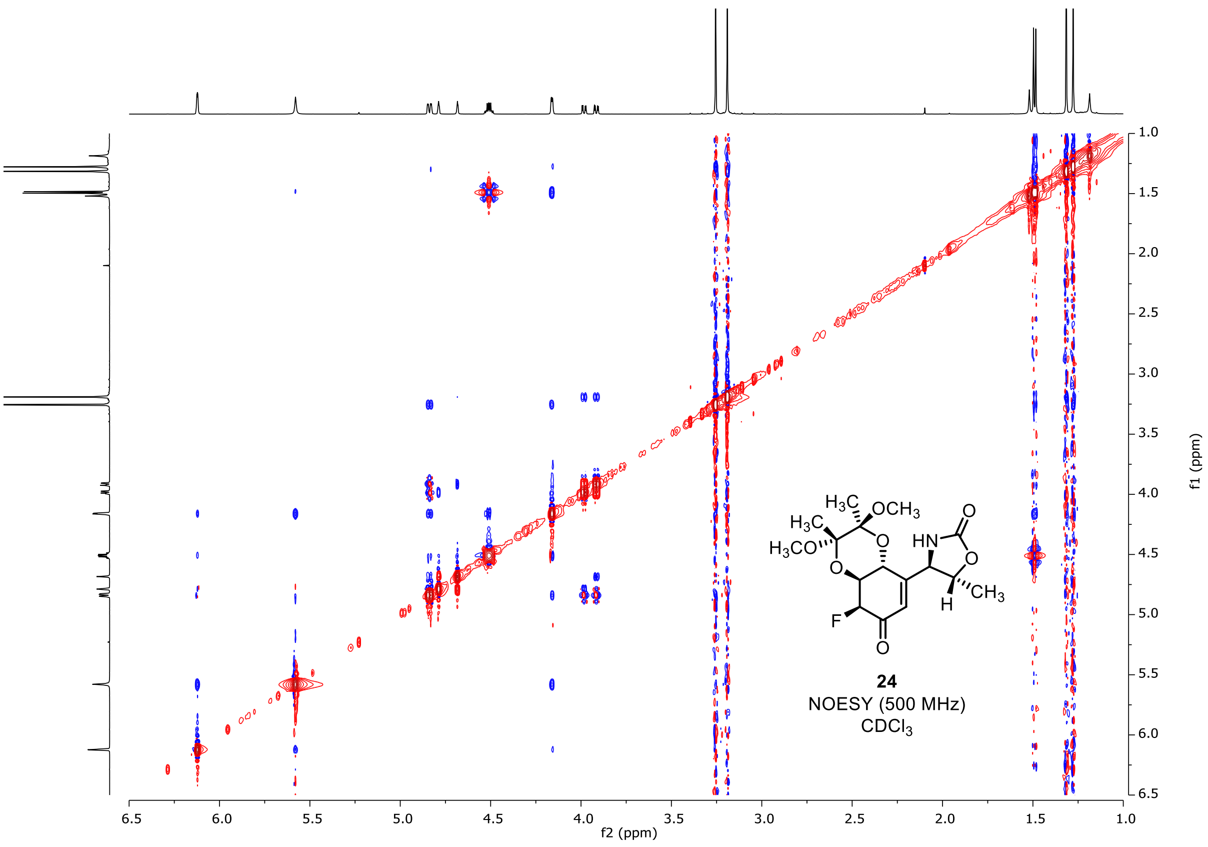
H3C
OCH3
H3C
H3CO
O
HN
O
O
O
CH3
H
F
O
24
NOESY (500 MHz)
CDCl3
f2 (ppm)
f1 (ppm)

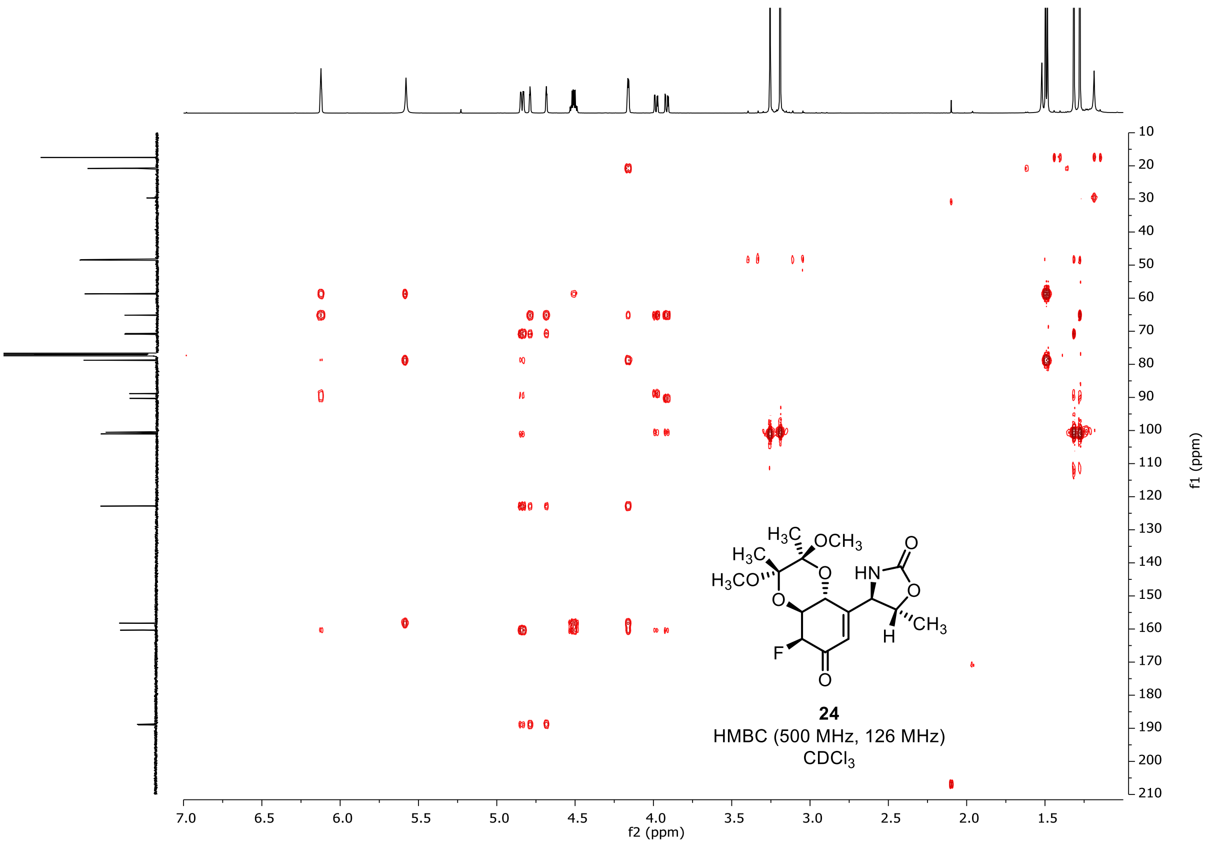
H3C
OCH3
H3C
H3CO
O
HN
O
O
O
CH3
H
F
O
24
HMBC (500 MHz, 126 MHz)
CDCl3
f2 (ppm)
f1 (ppm)

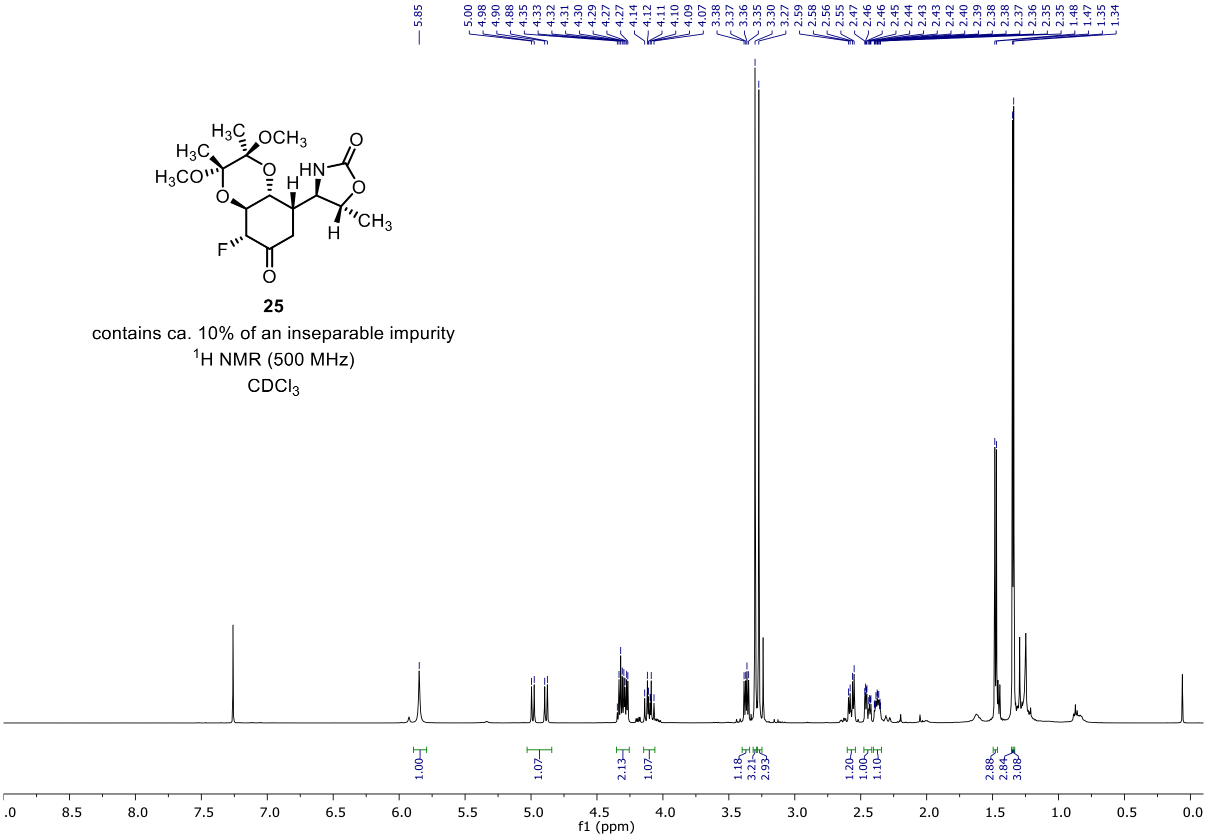

25

contains ca. 10% of an inseparable impurity

^{1}H NMR (500 MHz)

$CDCl_3$

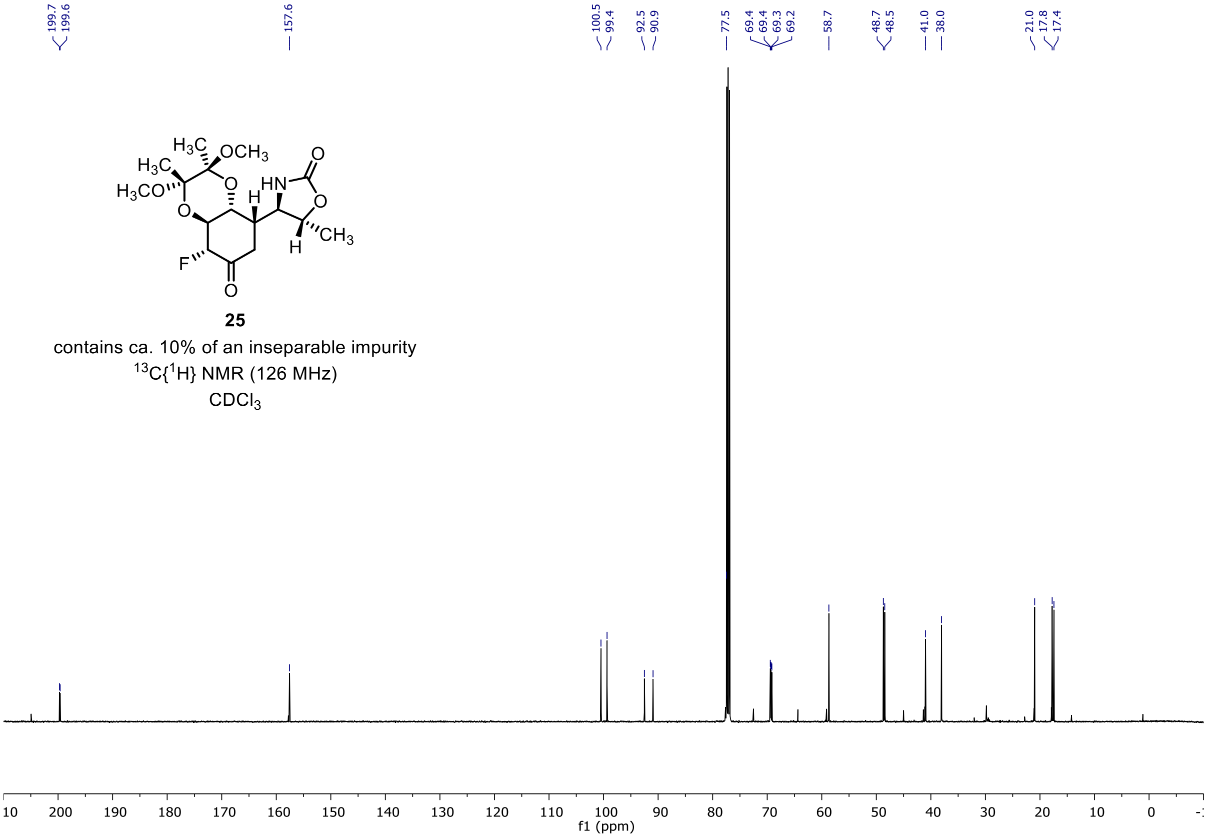

25

contains ca. 10% of an inseparable impurity

$^{13}C\{^{1}H\}$ NMR (126 MHz)

$CDCl_3$

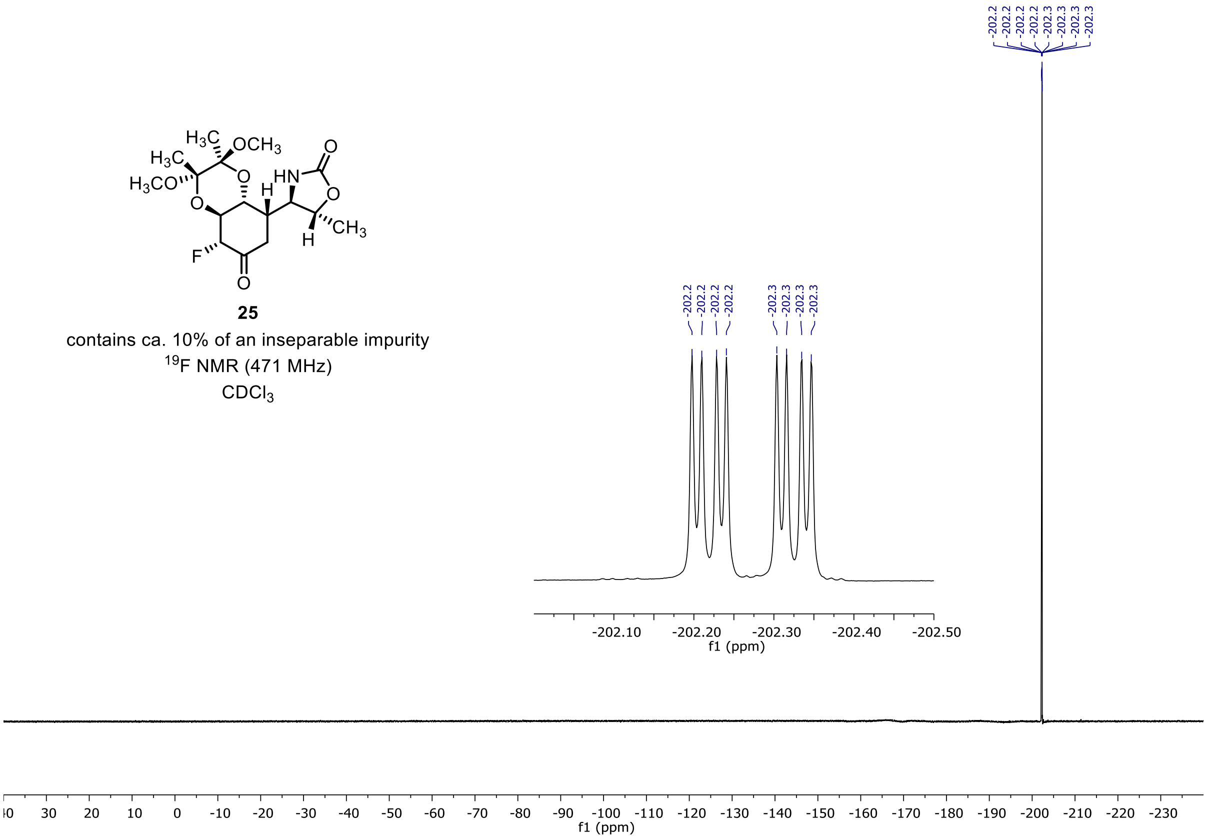

25
contains ca. 10% of an inseparable impurity
^{19}F NMR (471 MHz)
$CDCl_3$

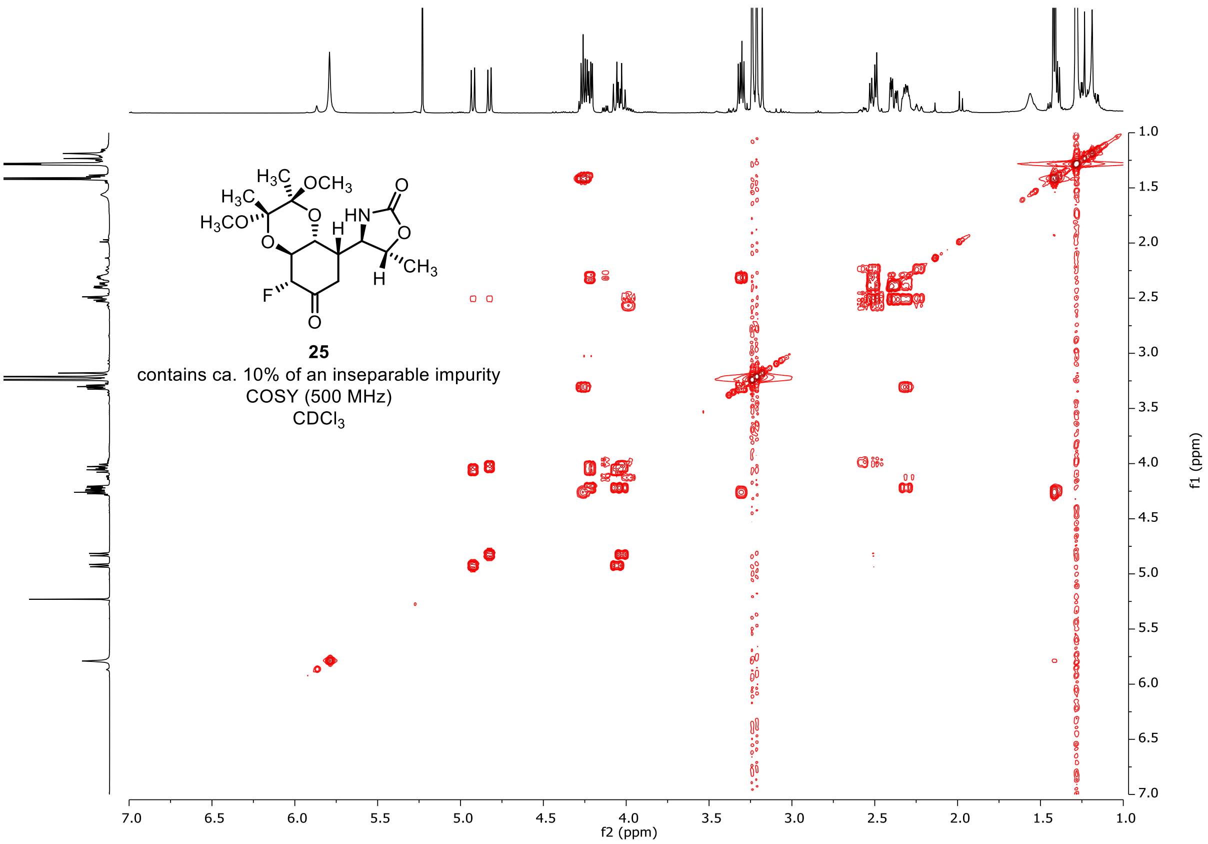

25
contains ca. 10% of an inseparable impurity
COSY (500 MHz)
$CDCl_3$

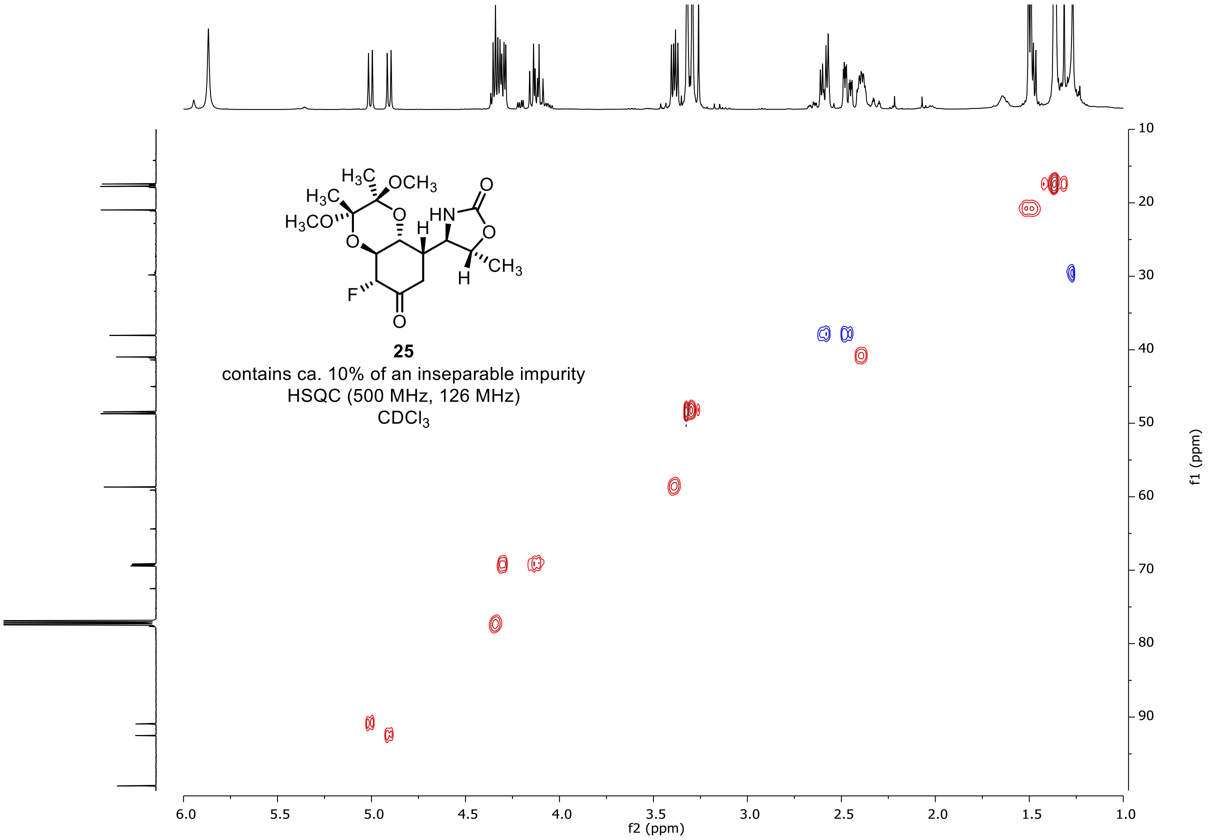

H3C
OCH3
H3C
H3CO
O
O
HN
O
O
H
CH3
H
F
O
25
contains ca. 10% of an inseparable impurity
HSQC (500 MHz, 126 MHz)
CDCl3
f2 (ppm)
f1 (ppm)

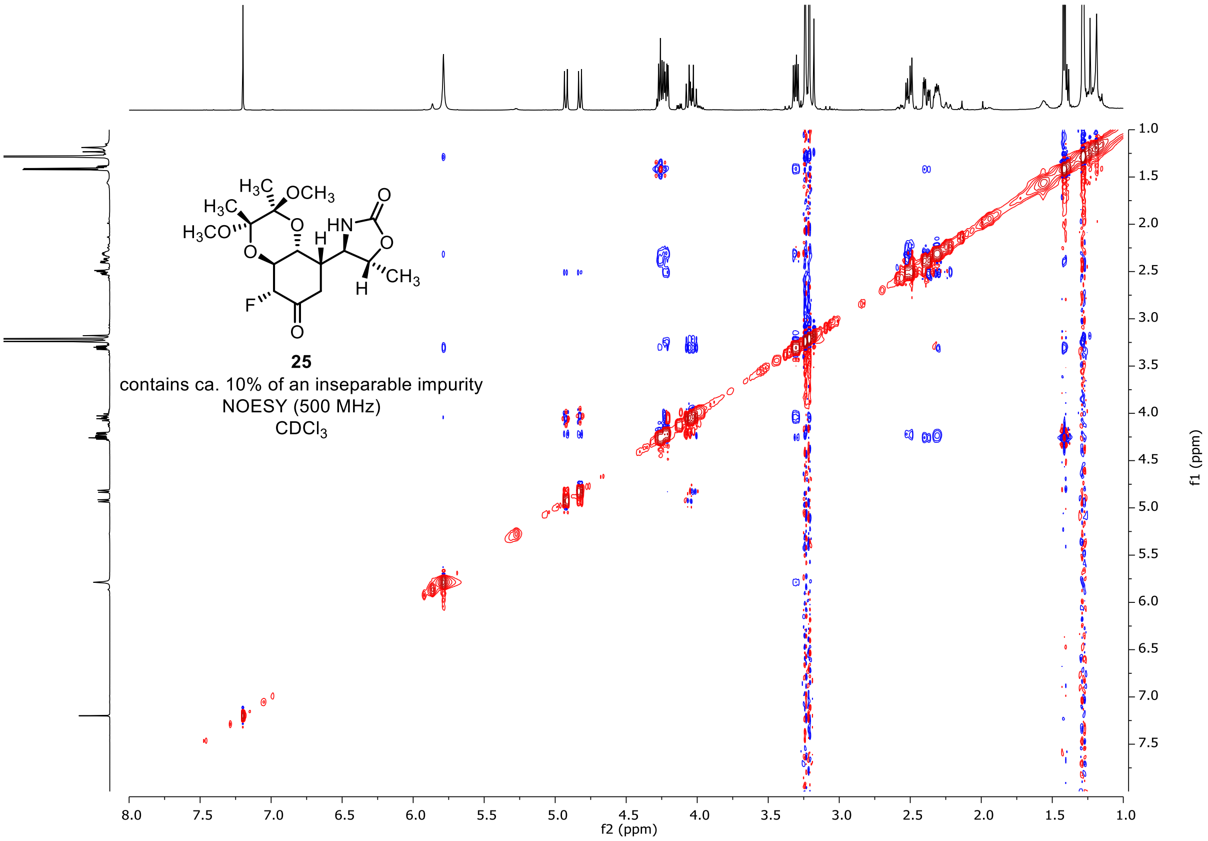

H3C
OCH3
H3C
H3CO
O
O
HN
O
O
H
CH3
H
F
O
25
contains ca. 10% of an inseparable impurity
NOESY (500 MHz)
CDCl3
f2 (ppm)
f1 (ppm)

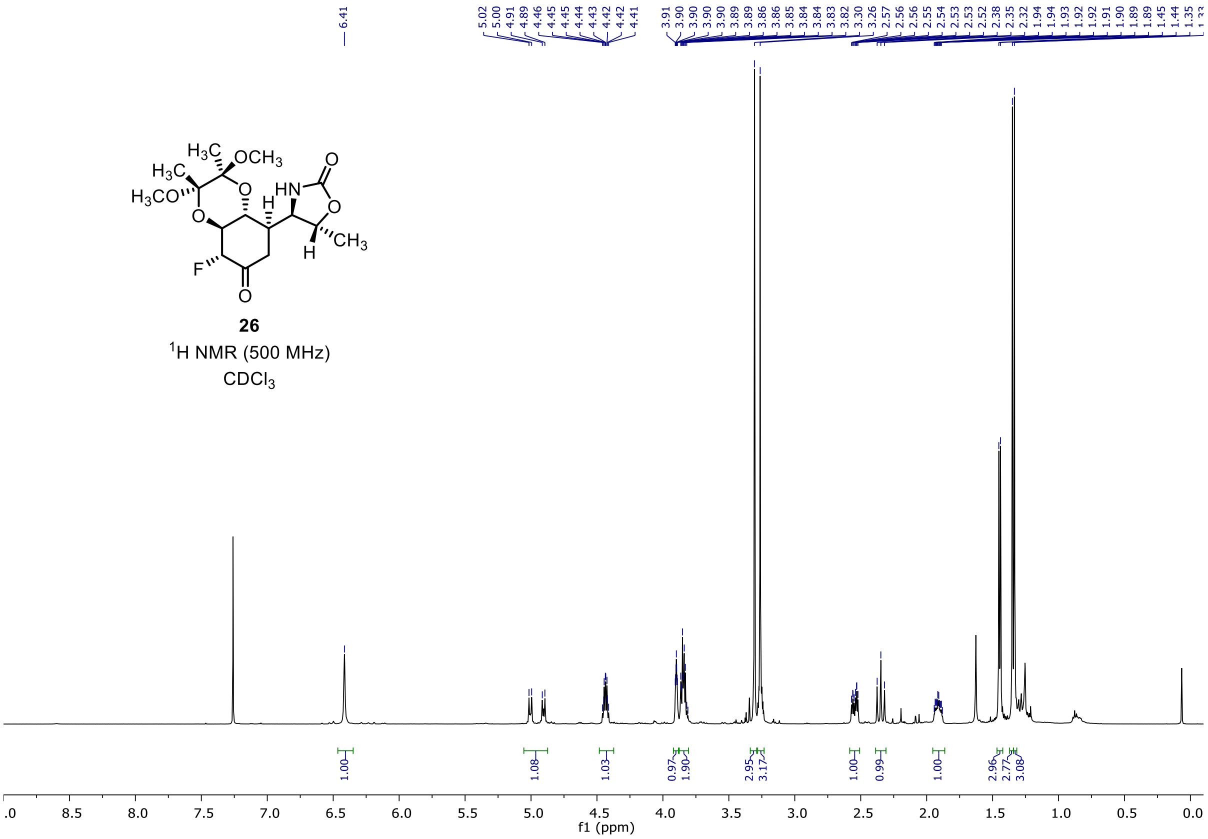

26
1H NMR (500 MHz)
CDCl3
6.41
5.02 5.00 4.91 4.89 4.46 4.45 4.45 4.44 4.43 4.42 4.42 4.41
3.91 3.90 3.90 3.90 3.89 3.89 3.86 3.86 3.85 3.84 3.84 3.84 3.83 3.82 3.30 3.26
2.57 2.56 2.56 2.55 2.54 2.53 2.53 2.52 2.38 2.35 2.32
1.94 1.94 1.93 1.92 1.92 1.91 1.90 1.89 1.89 1.45 1.44 1.35
1.00 1.08 1.03 0.97 1.90 2.95 3.17 1.00 0.99 1.00 2.96 2.77 3.08
f1 (ppm)

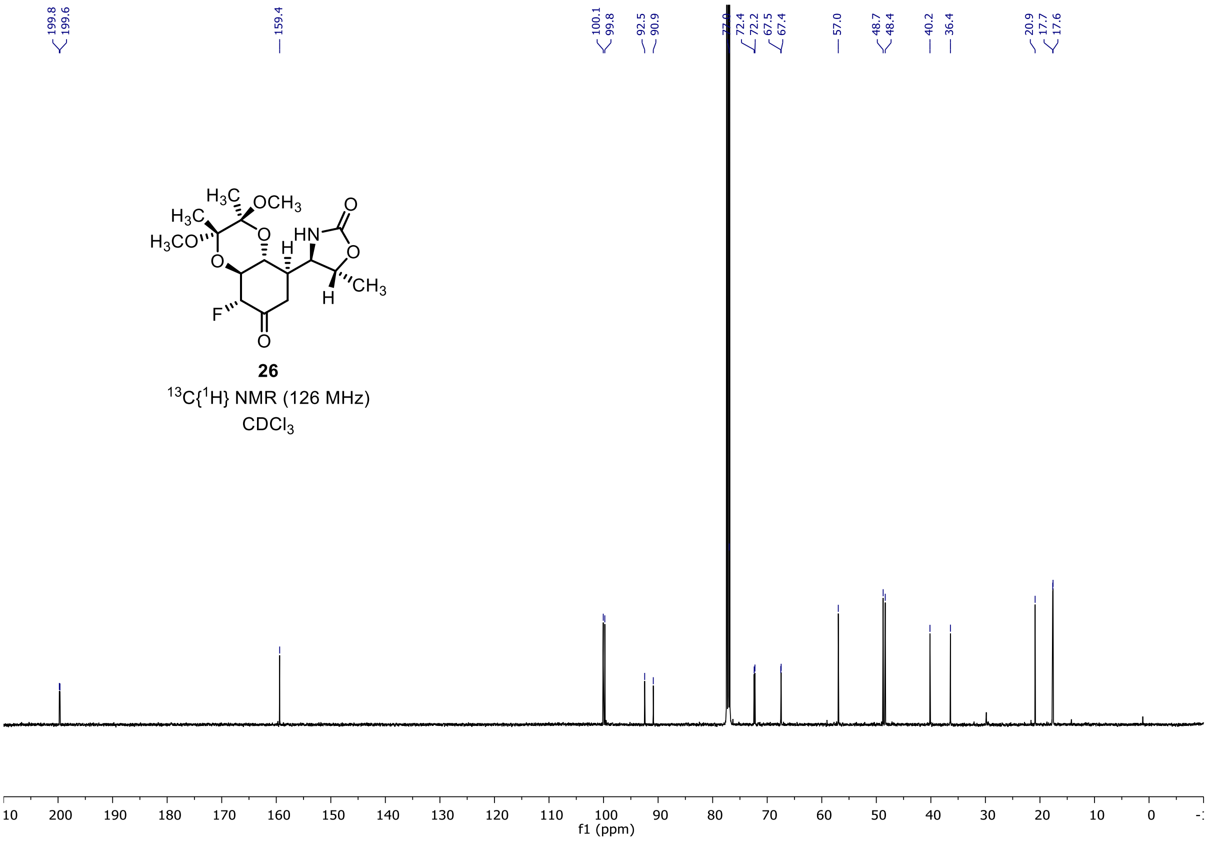

26
13C{1H} NMR (126 MHz)
CDCl3
199.8 199.6 159.4 100.1 99.8 92.5 90.9 72.4 72.2 67.5 67.4 57.0 48.7 48.4 40.2 36.4 20.9 17.7 17.6
f1 (ppm)

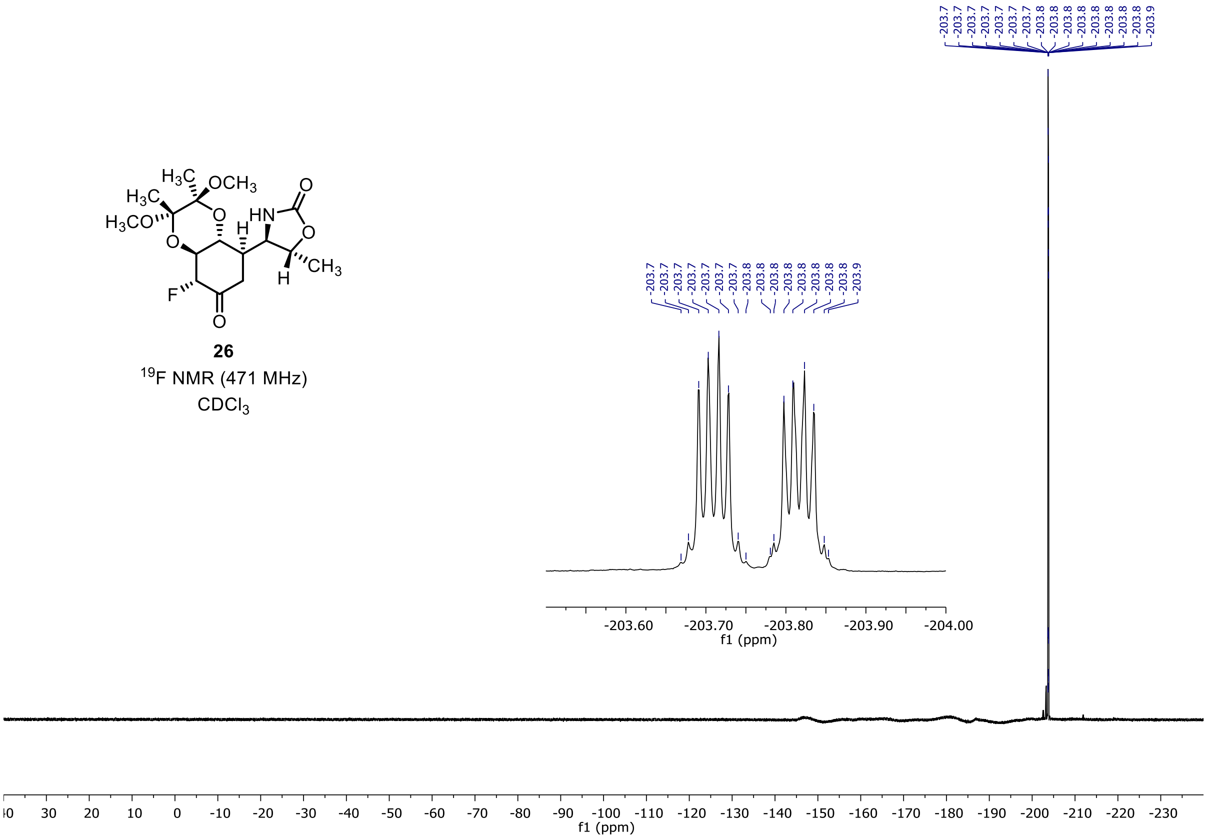
26
19F NMR (471 MHz)
CDCl3
f1 (ppm)

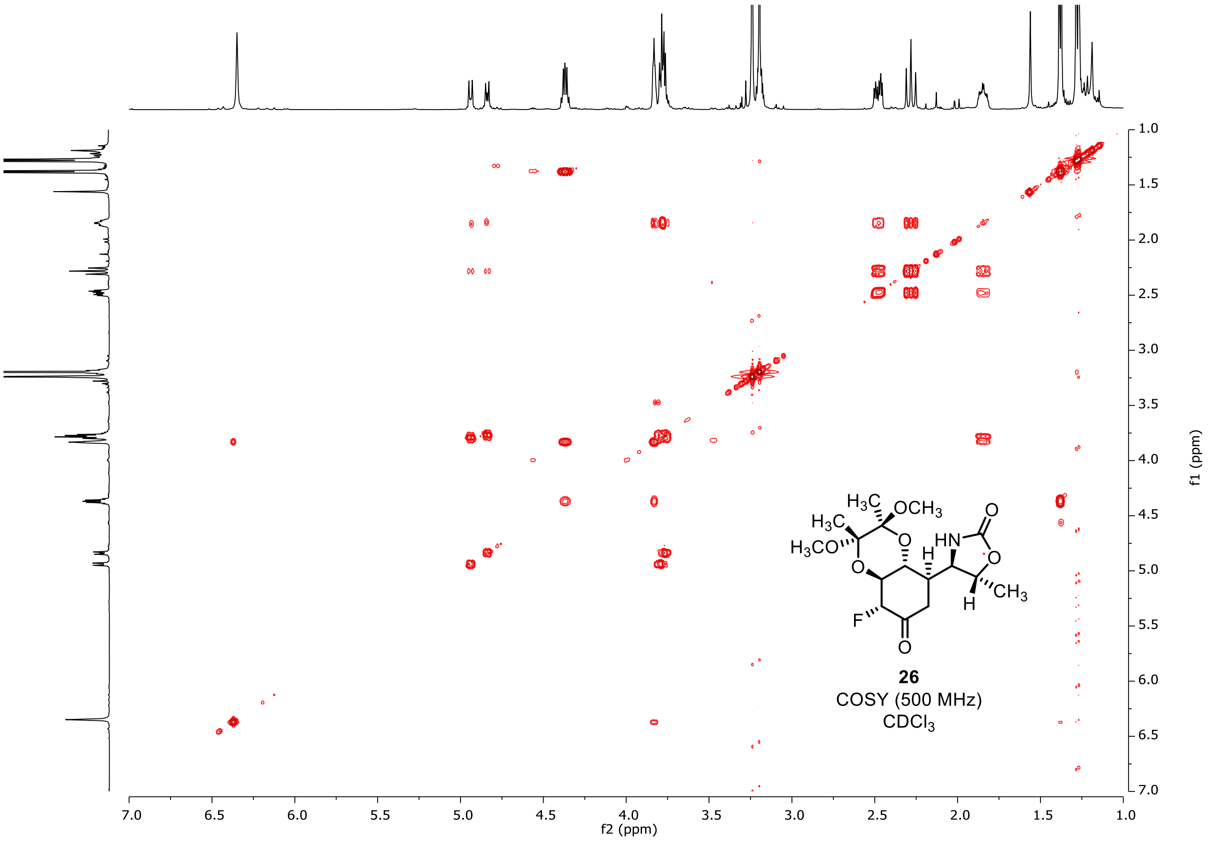
26
COSY (500 MHz)
CDCl3
f2 (ppm)
f1 (ppm)

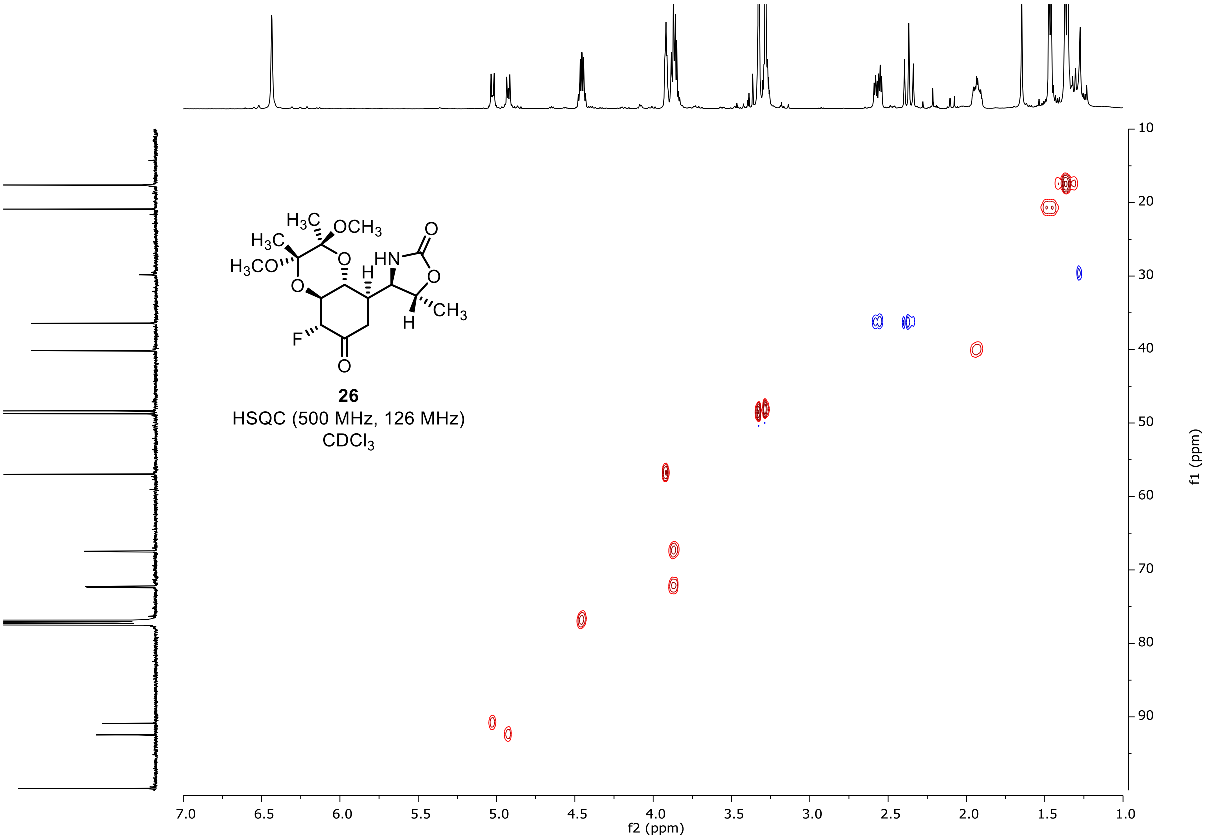

26
HSQC (500 MHz, 126 MHz)
$CDCl_3$
f2 (ppm)
f1 (ppm)

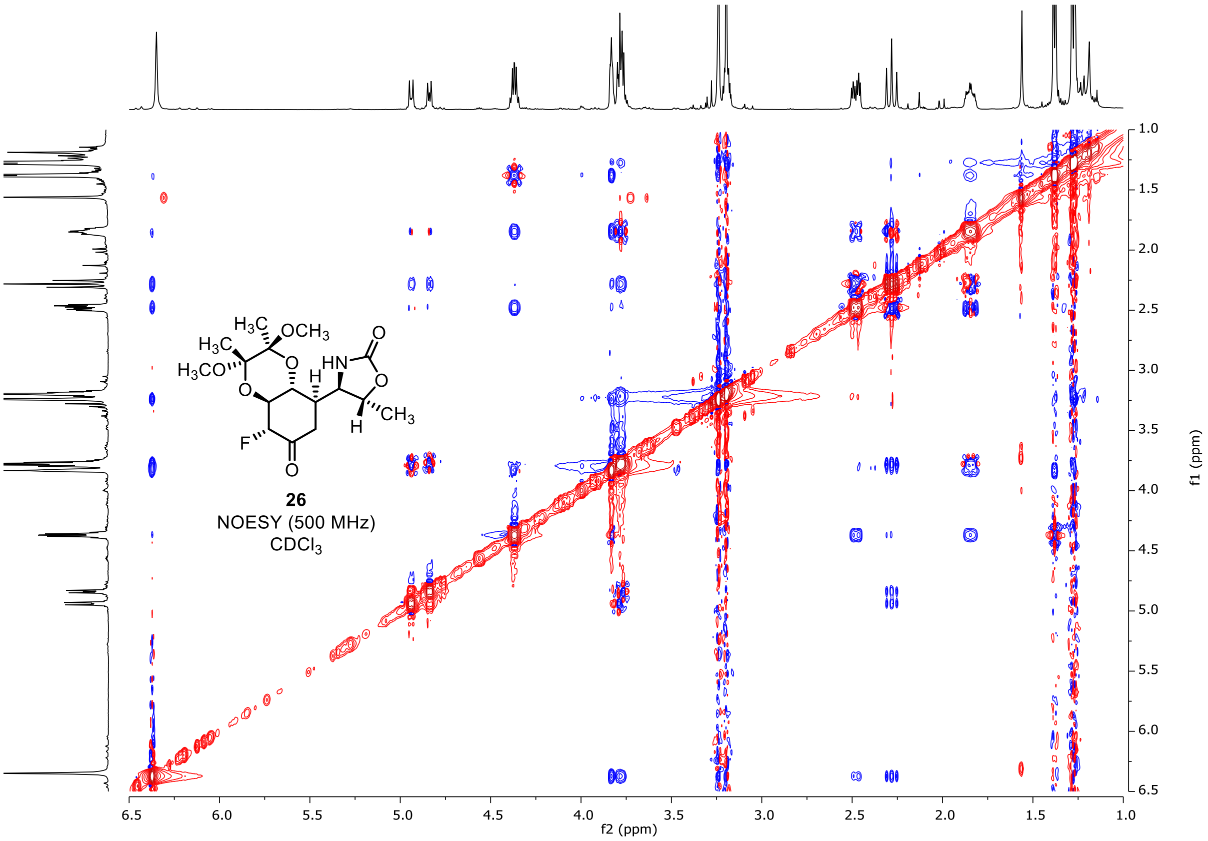

26
NOESY (500 MHz)
$CDCl_3$
f2 (ppm)
f1 (ppm)

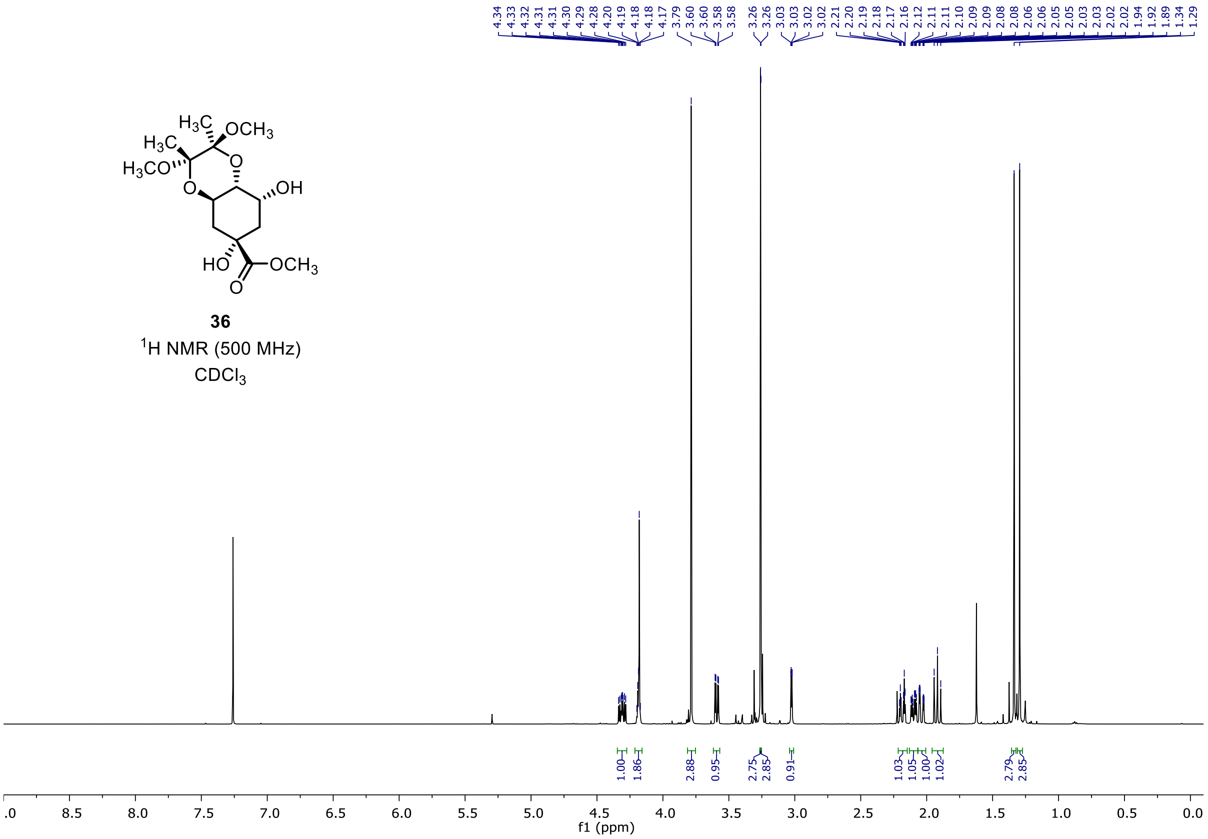

36
^{1}H NMR (500 MHz)
$CDCl_3$

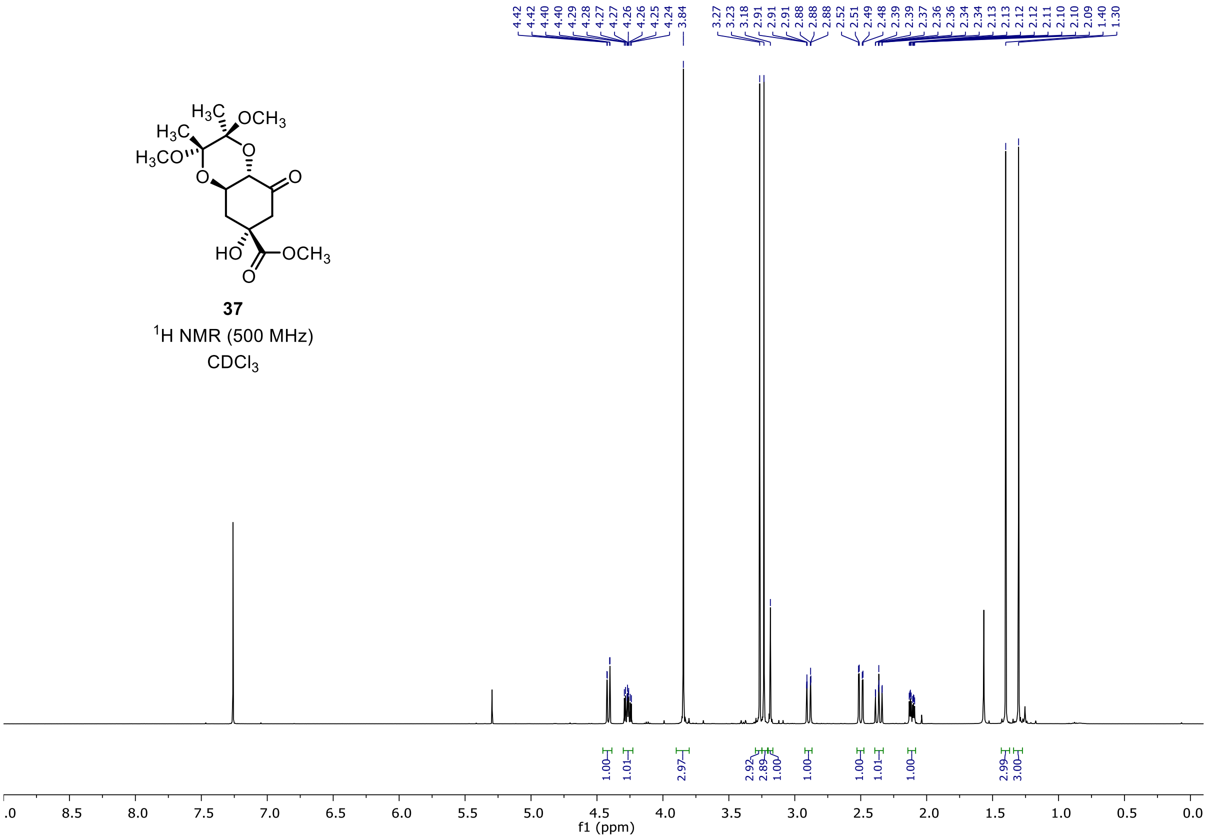

37
^{1}H NMR (500 MHz)
$CDCl_3$

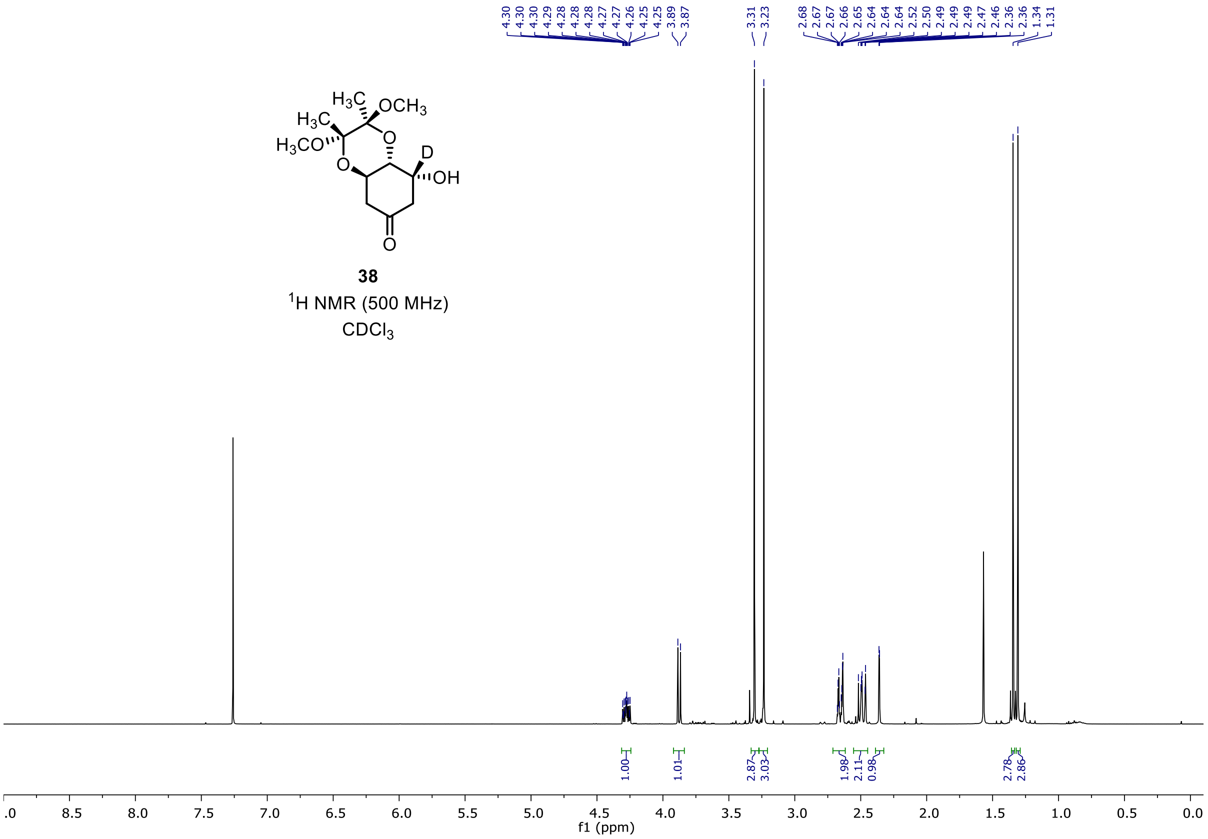

38
^1H NMR (500 MHz)
$CDCl_3$

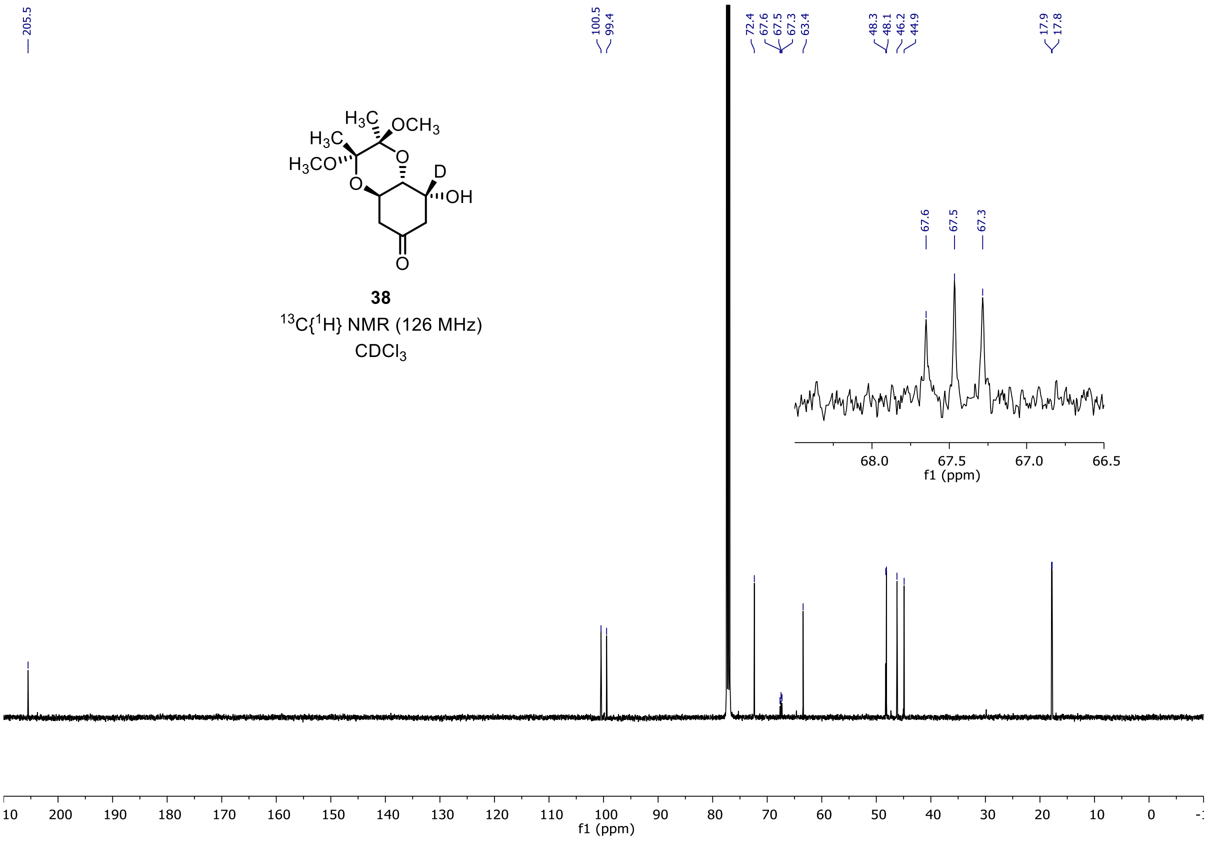

38
$^{13}C\{^1H\}$ NMR (126 MHz)
$CDCl_3$

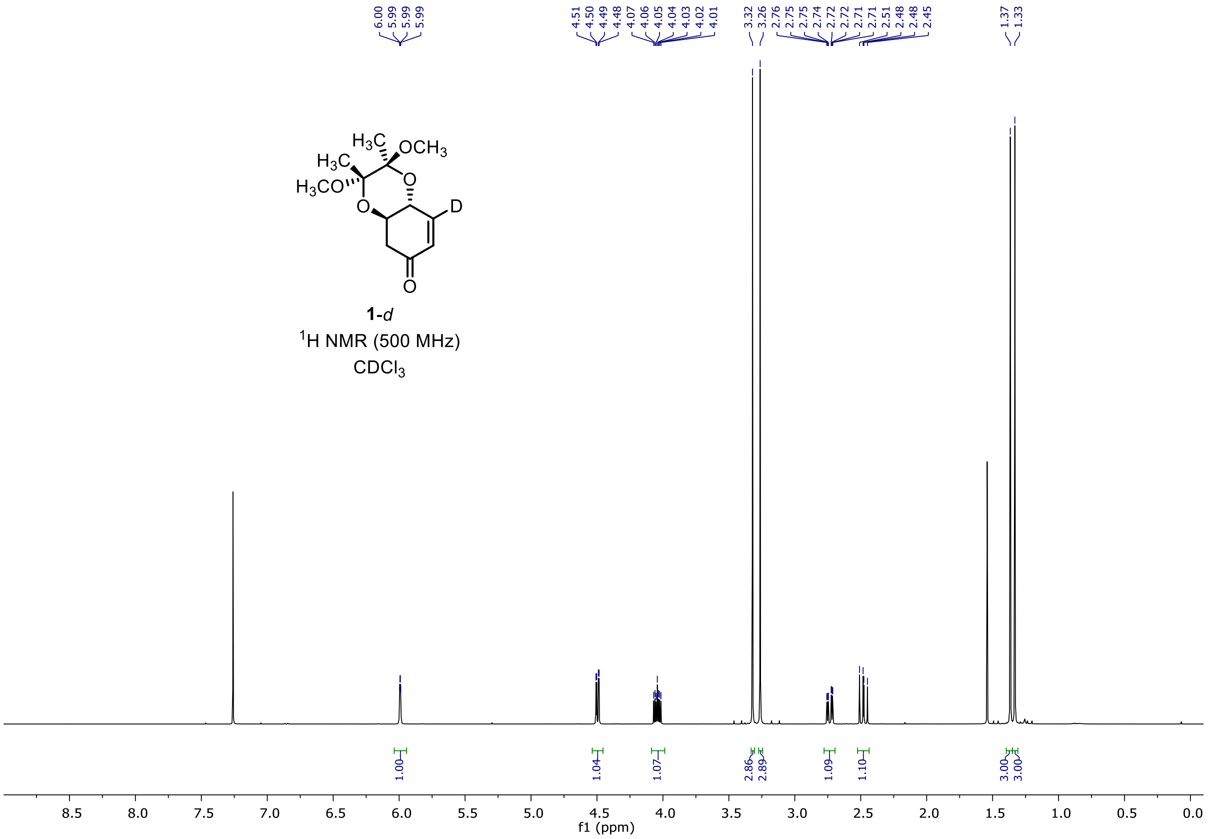
6.00 5.99 5.99 5.99
4.51 4.50 4.49 4.48 4.07 4.06 4.05 4.04 4.03 4.02 4.01
3.32 3.26 2.76 2.75 2.75 2.74 2.72 2.72 2.71 2.71 2.51 2.48 2.48 2.45
1.37 1.33
H3C OCH3 H3C H3CO O O D O
1-d
1H NMR (500 MHz)
CDCl3
1.00 1.04 1.07 2.86 2.89 1.09 1.10 3.00 3.00
f1 (ppm)

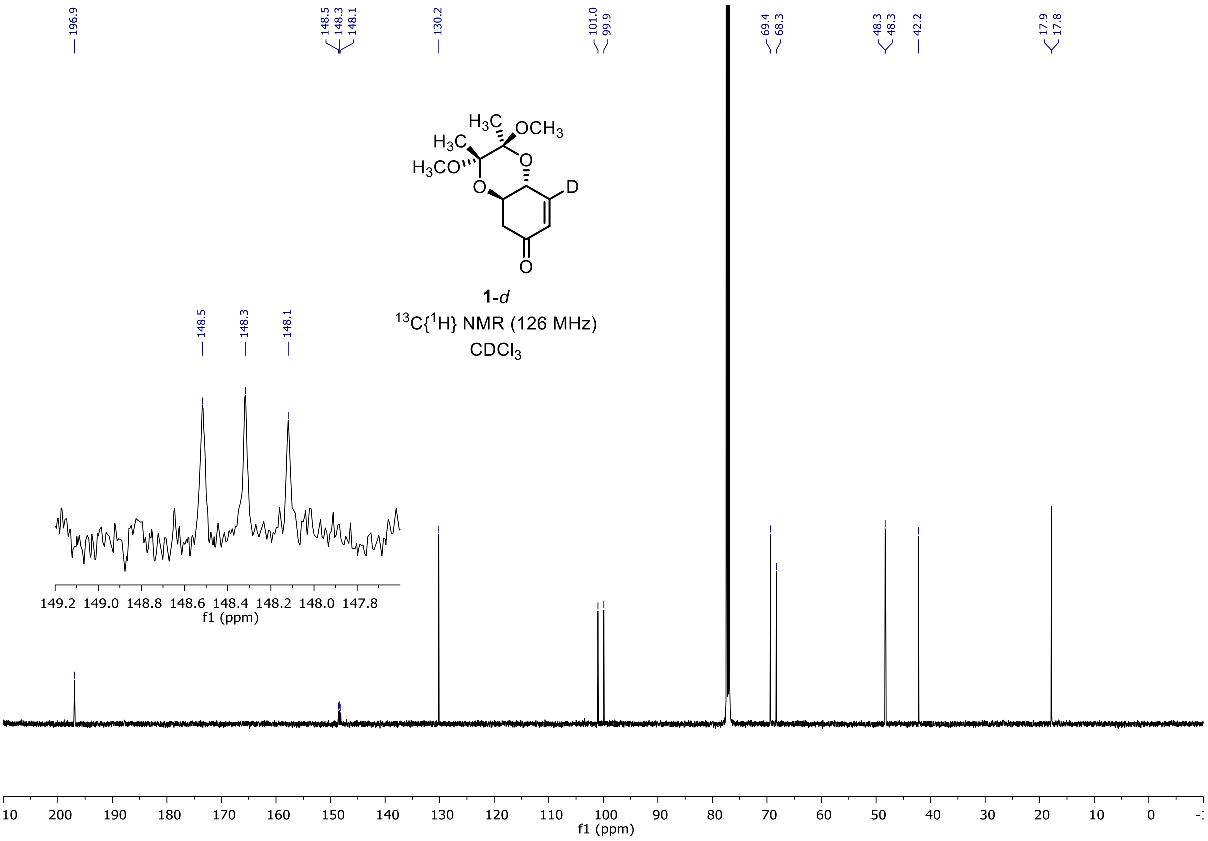
196.9
148.5 148.3 148.1
130.2
101.0 99.9
69.4 68.3
48.3 48.3 42.2
17.9 17.8
H3C OCH3 H3C H3CO O O D O
1-d
13C{1H} NMR (126 MHz)
CDCl3
f1 (ppm)

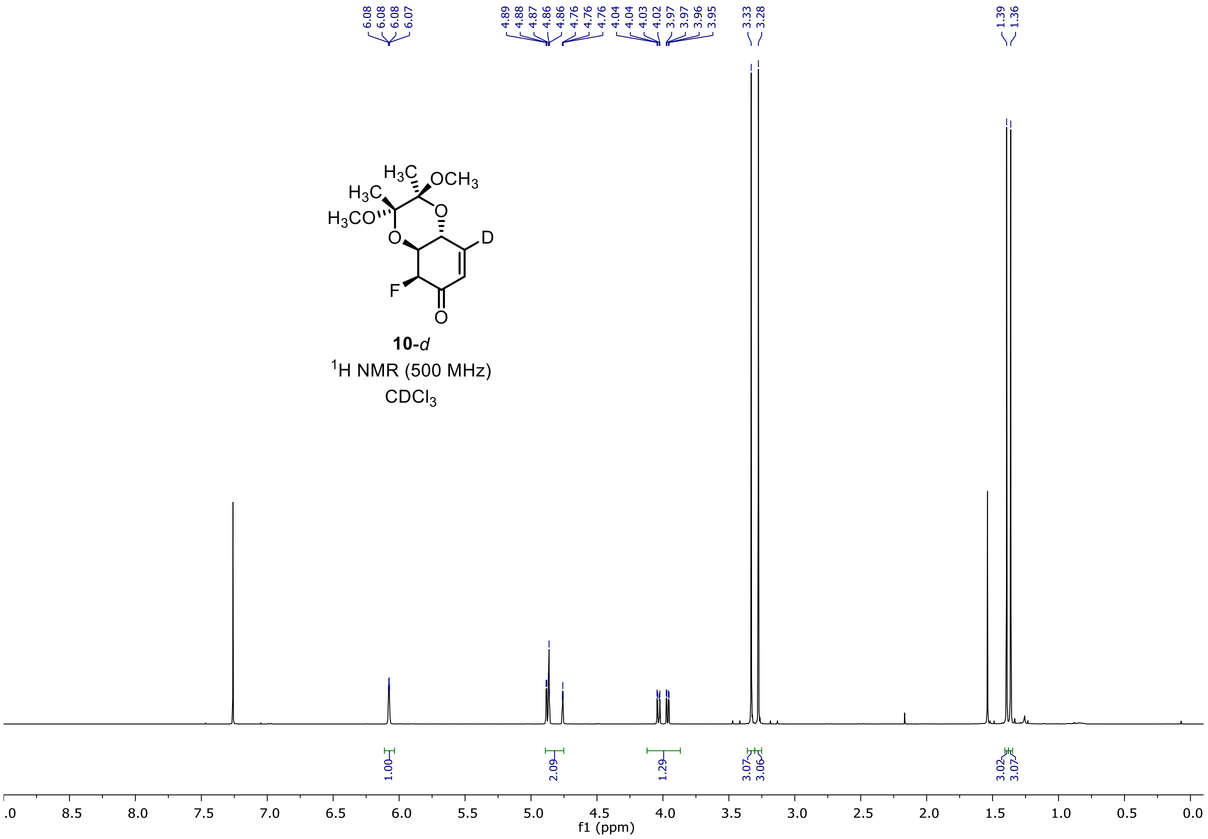
6.08
6.08
6.08
6.07
4.89
4.88
4.87
4.86
4.86
4.76
4.76
4.76
4.04
4.04
4.03
4.02
3.97
3.97
3.96
3.95
3.33
3.28
1.39
1.36
H_3C
OCH_3
H_3C
H_3CO
O
O
D
F
O
10-*d*
^1H NMR (500 MHz)
$CDCl_3$
1.00
2.09
1.29
3.07
3.06
3.02
3.07
f1 (ppm)

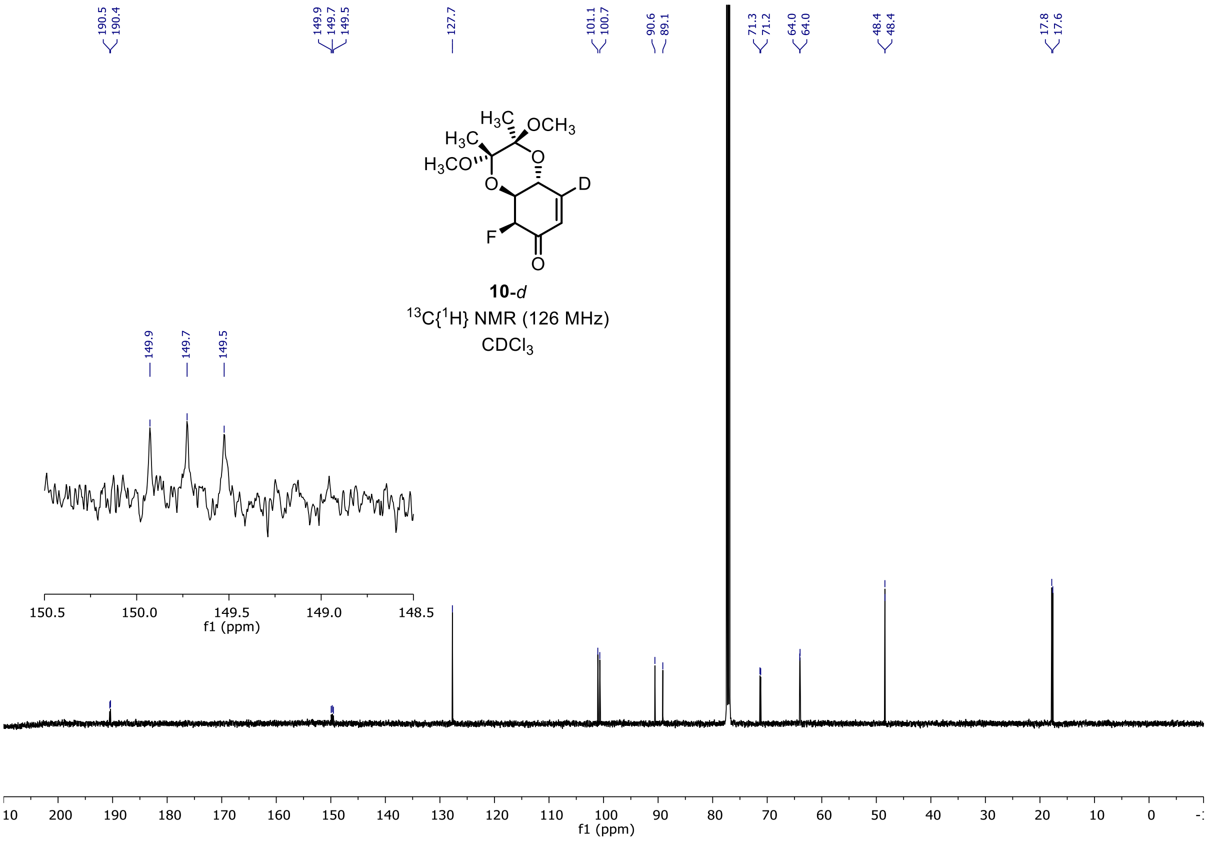
190.5
190.4
149.9
149.7
149.5
127.7
101.1
100.7
90.6
89.1
71.3
71.2
64.0
64.0
48.4
48.4
17.8
17.6
H_3C
OCH_3
H_3C
H_3CO
O
O
D
F
O
10-*d*
$^{13}C\{^1H\}$ NMR (126 MHz)
$CDCl_3$
149.9
149.7
149.5
f1 (ppm)
f1 (ppm)

10-*d*
^{19}F NMR (471 MHz)
$CDCl_3$

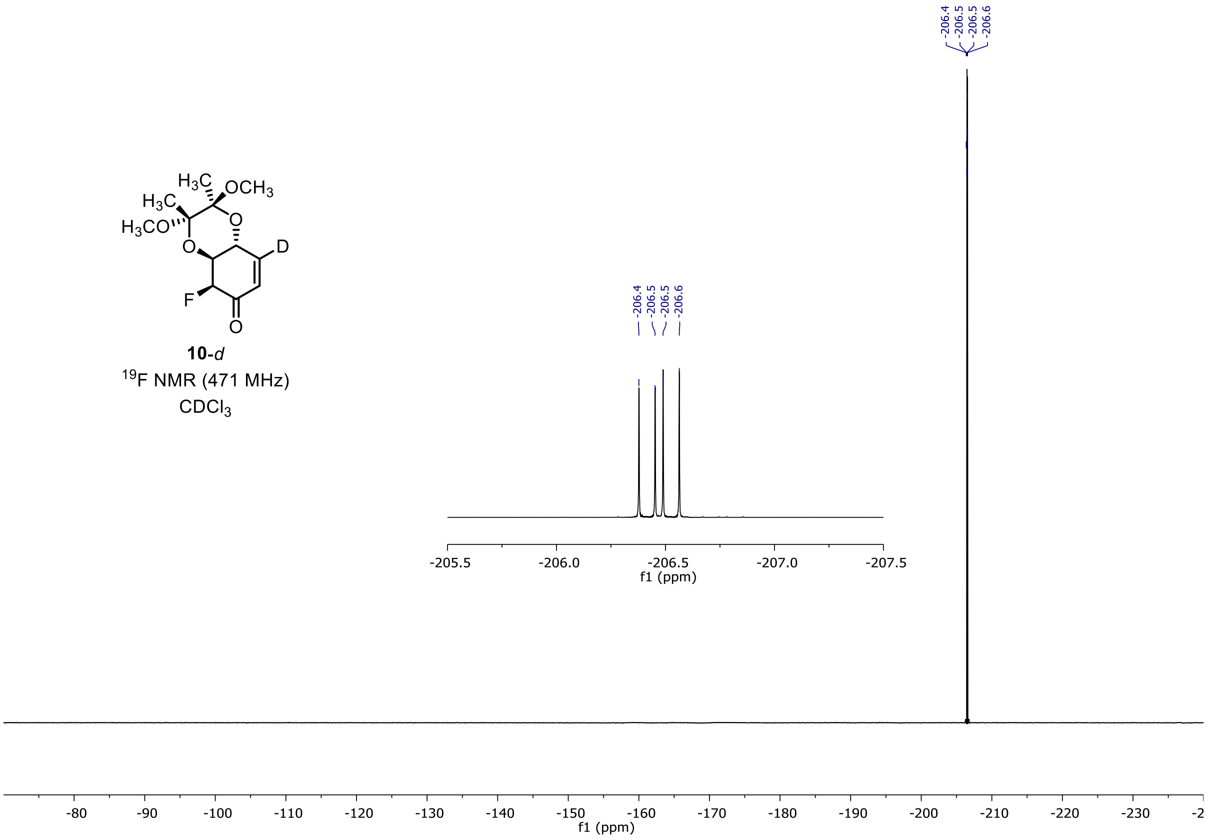

39
^{1}H NMR (500 MHz)
$CDCl_3$

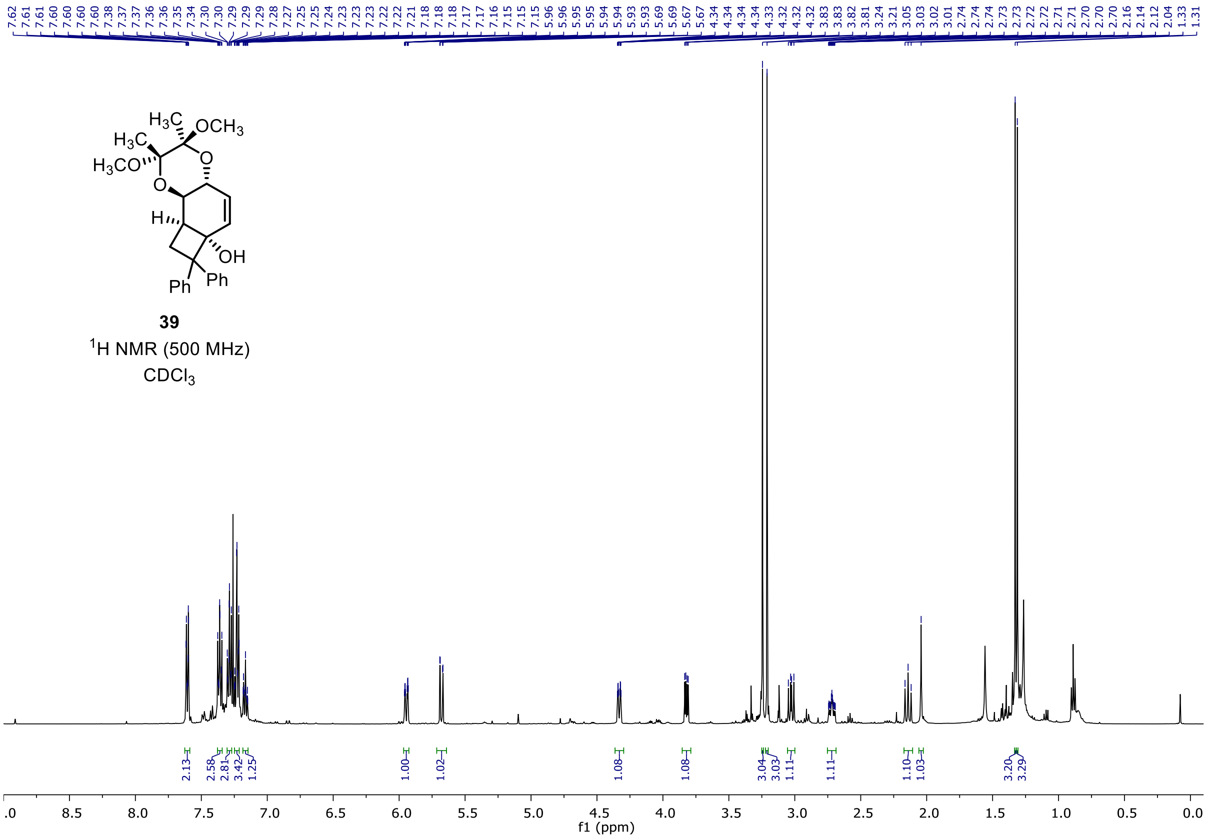

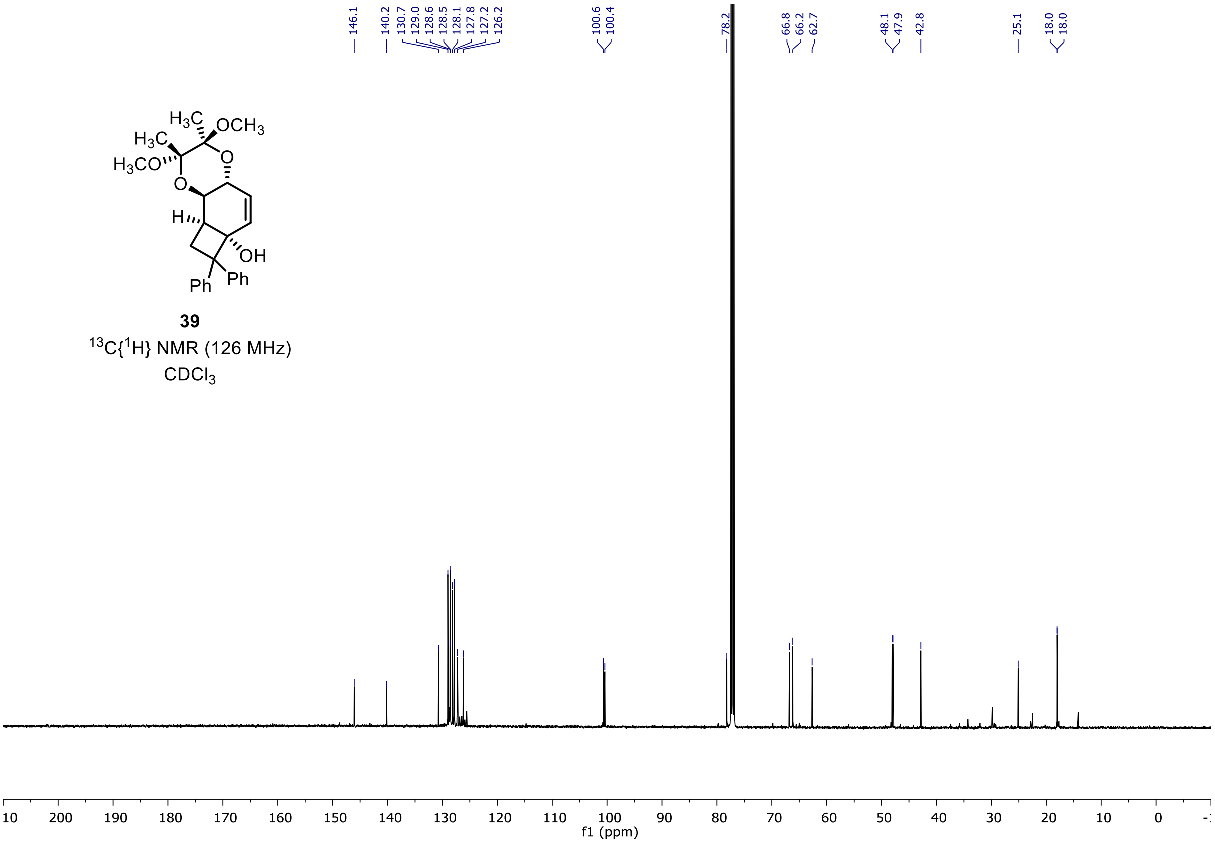
146.1
140.2
130.7
129.0
128.6
128.5
128.1
127.8
127.2
126.2
100.6
100.4
78.2
66.8
66.2
62.7
48.1
47.9
42.8
25.1
18.0
18.0
H3C
OCH3
H3CO
O
H
OH
Ph
39
13C{1H} NMR (126 MHz)
CDCl3
f1 (ppm)

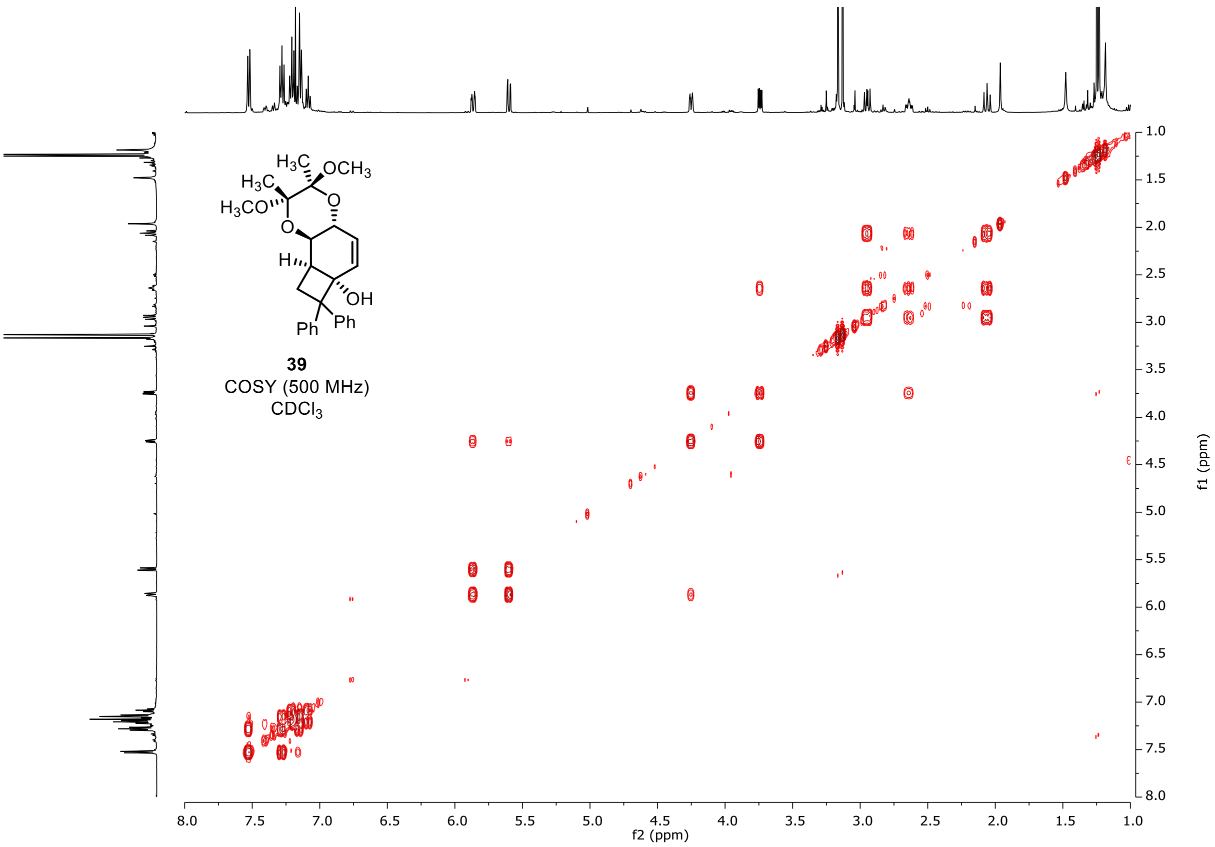
H3C
OCH3
H3CO
O
H
OH
Ph
39
COSY (500 MHz)
CDCl3
f2 (ppm)
f1 (ppm)

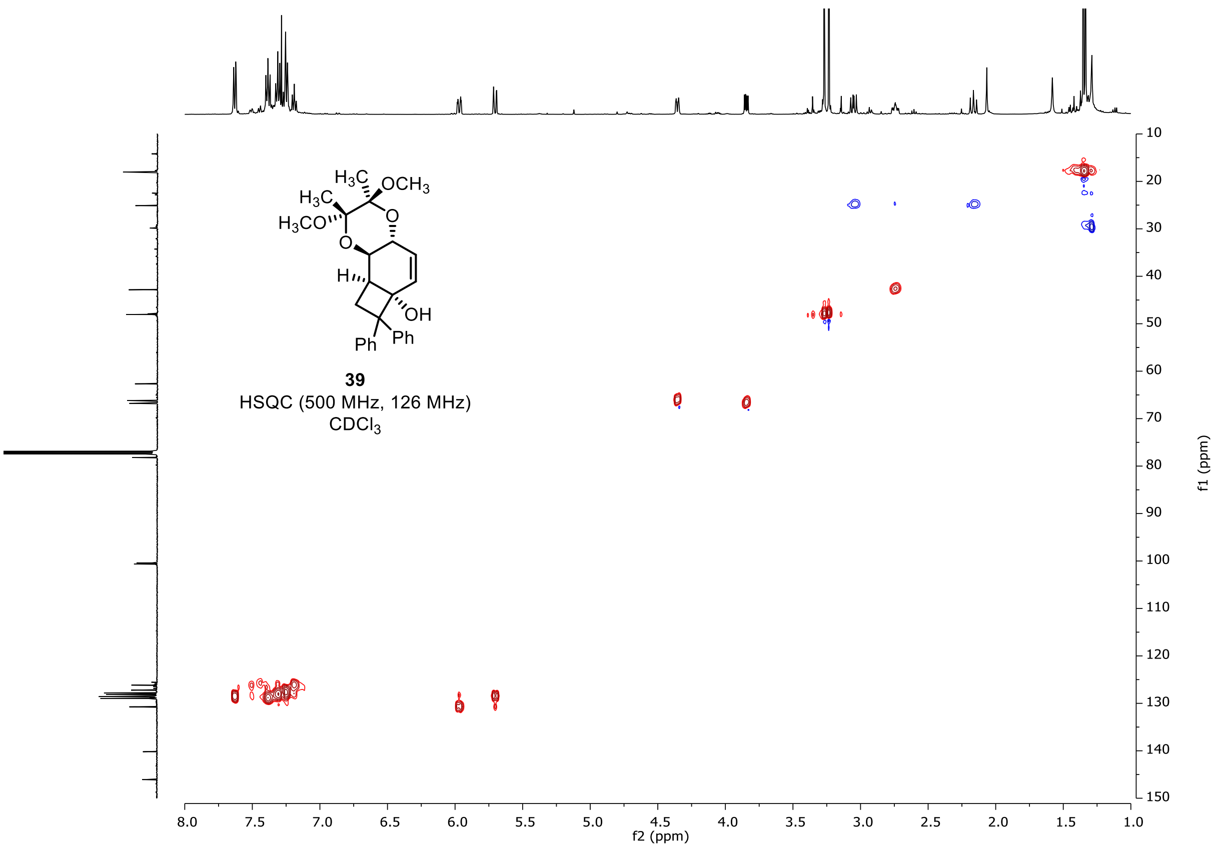

39
HSQC (500 MHz, 126 MHz)
$CDCl_3$
f2 (ppm)
f1 (ppm)

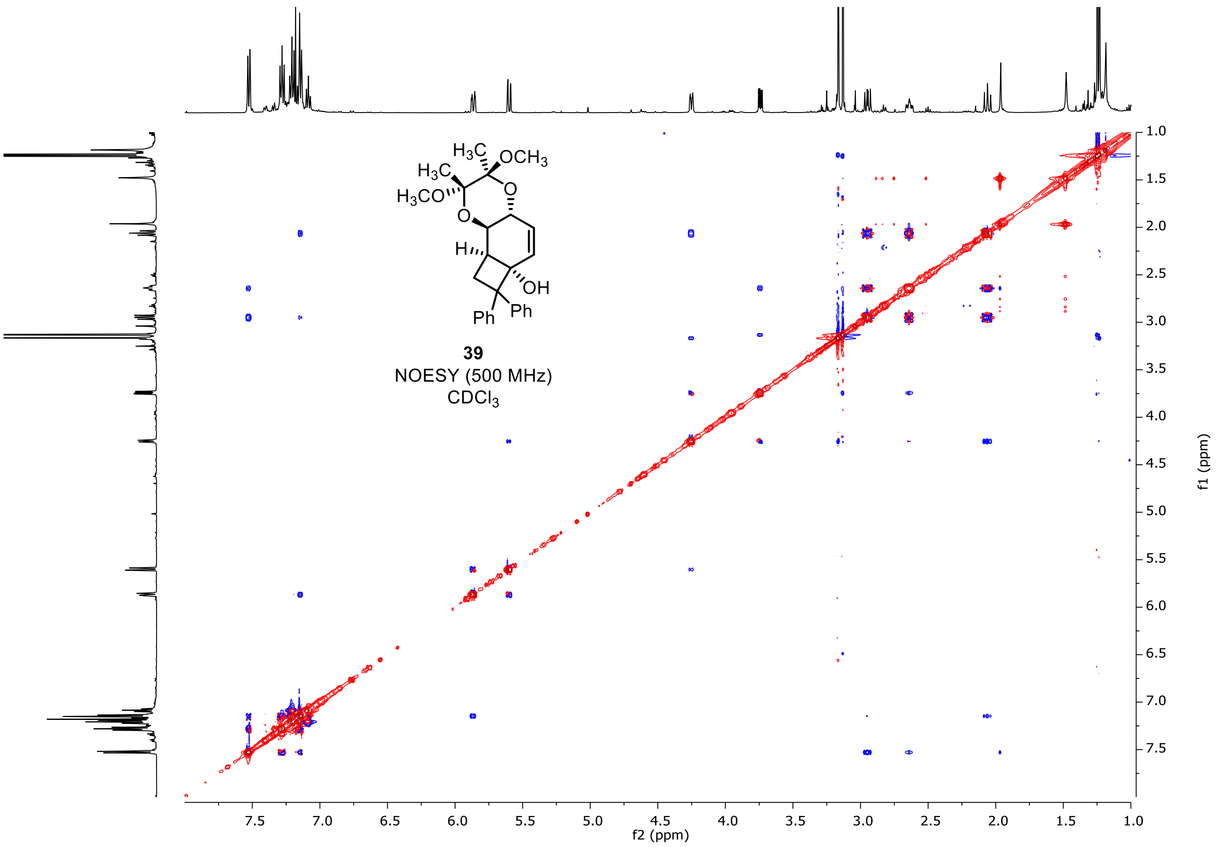

39
NOESY (500 MHz)
$CDCl_3$
f2 (ppm)
f1 (ppm)

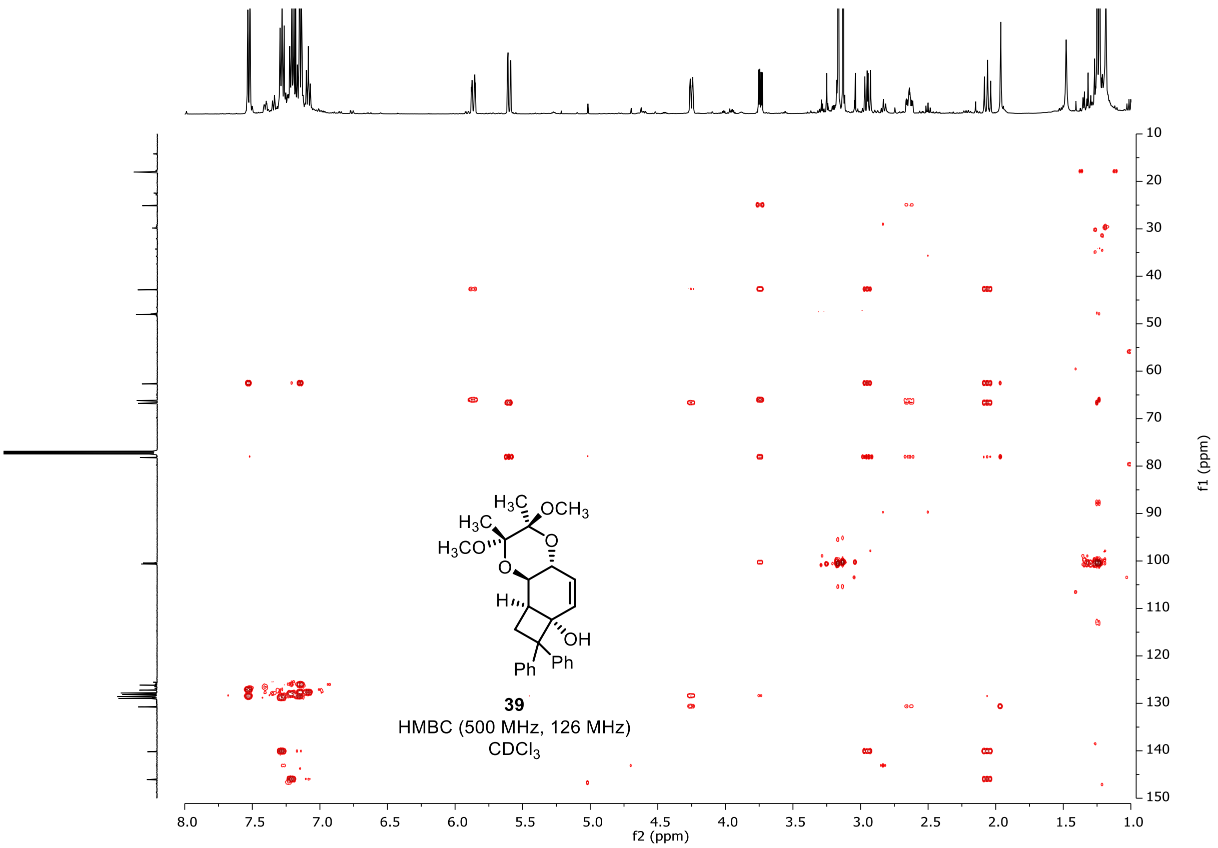

39
HMBC (500 MHz, 126 MHz)
$CDCl_3$

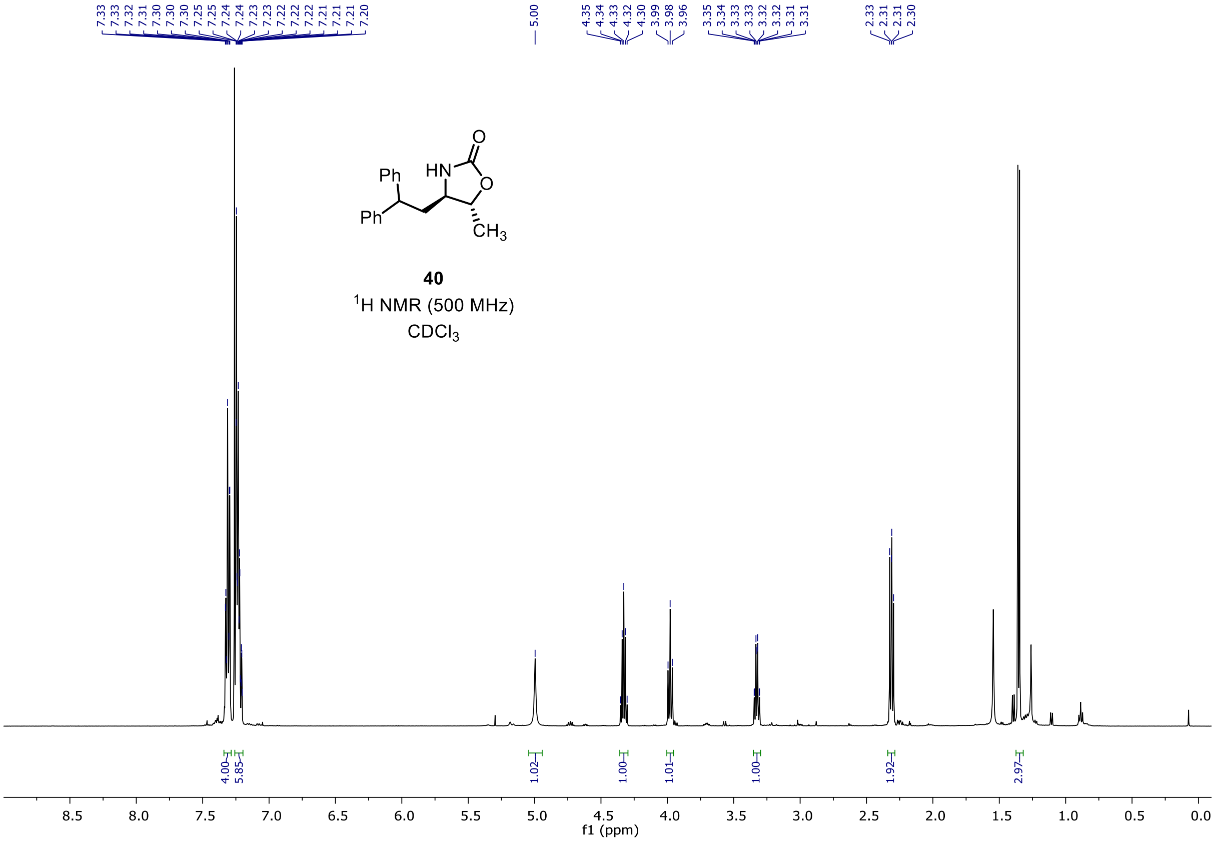

40
^{1}H NMR (500 MHz)
$CDCl_3$

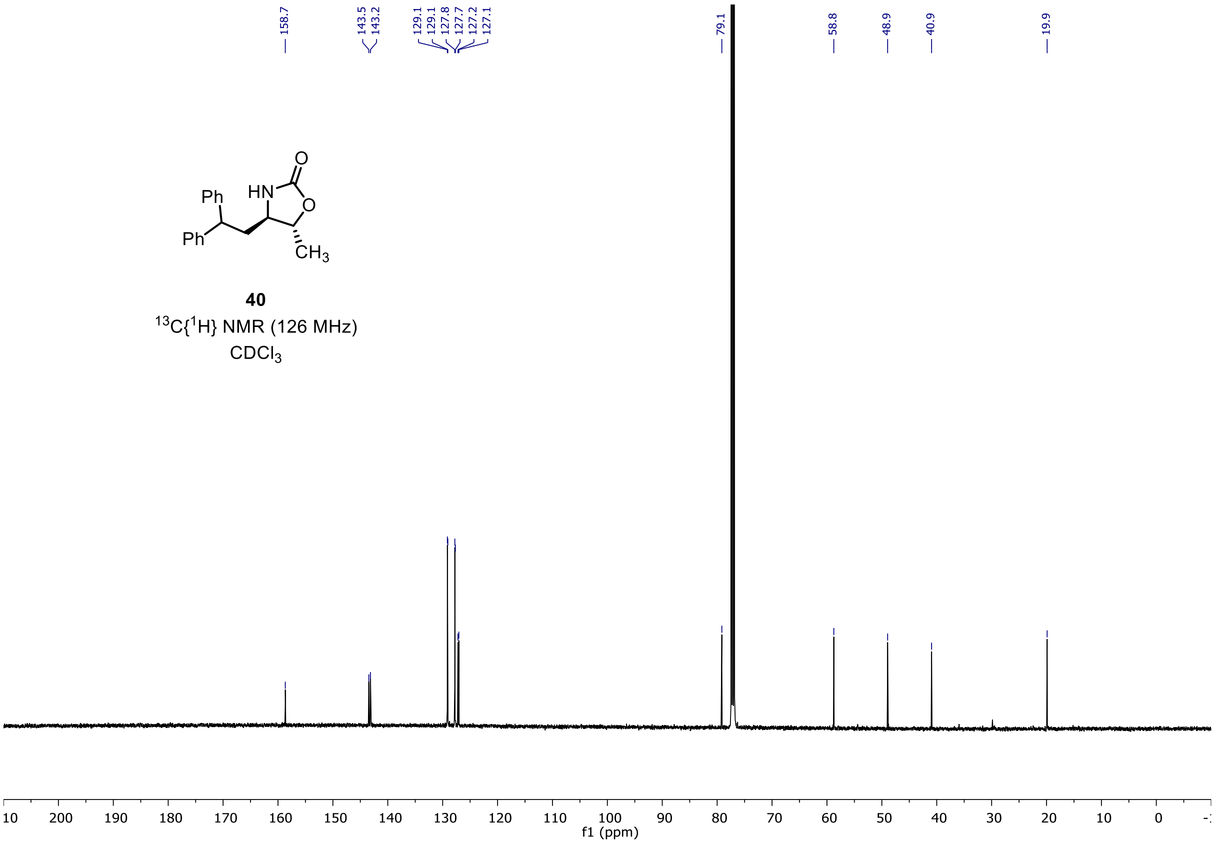

158.7
143.5
143.2
129.1
129.1
127.8
127.7
127.2
127.1
79.1
58.8
48.9
40.9
19.9
40
^{13}C{^{1}H} NMR (126 MHz)
$CDCl_3$
f1 (ppm)

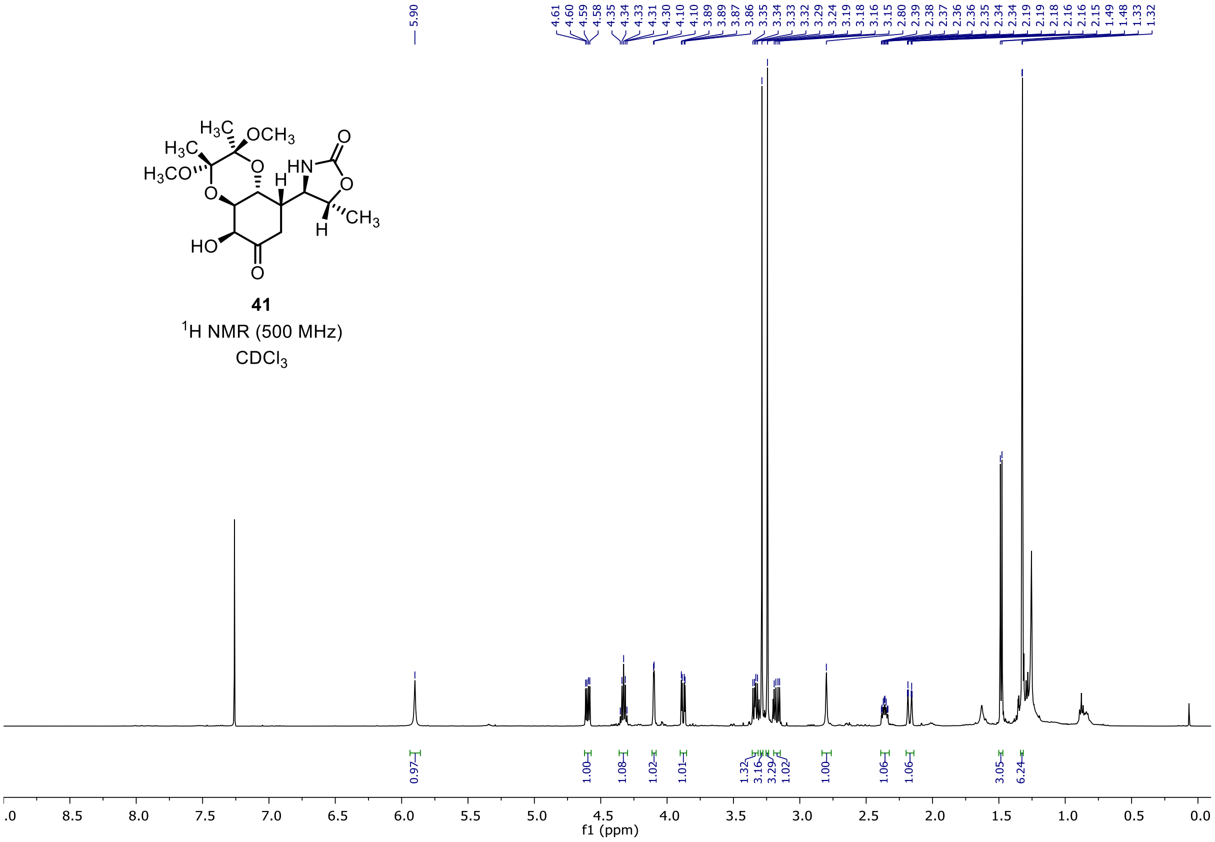

5.90
41
^{1}H NMR (500 MHz)
$CDCl_3$
f1 (ppm)

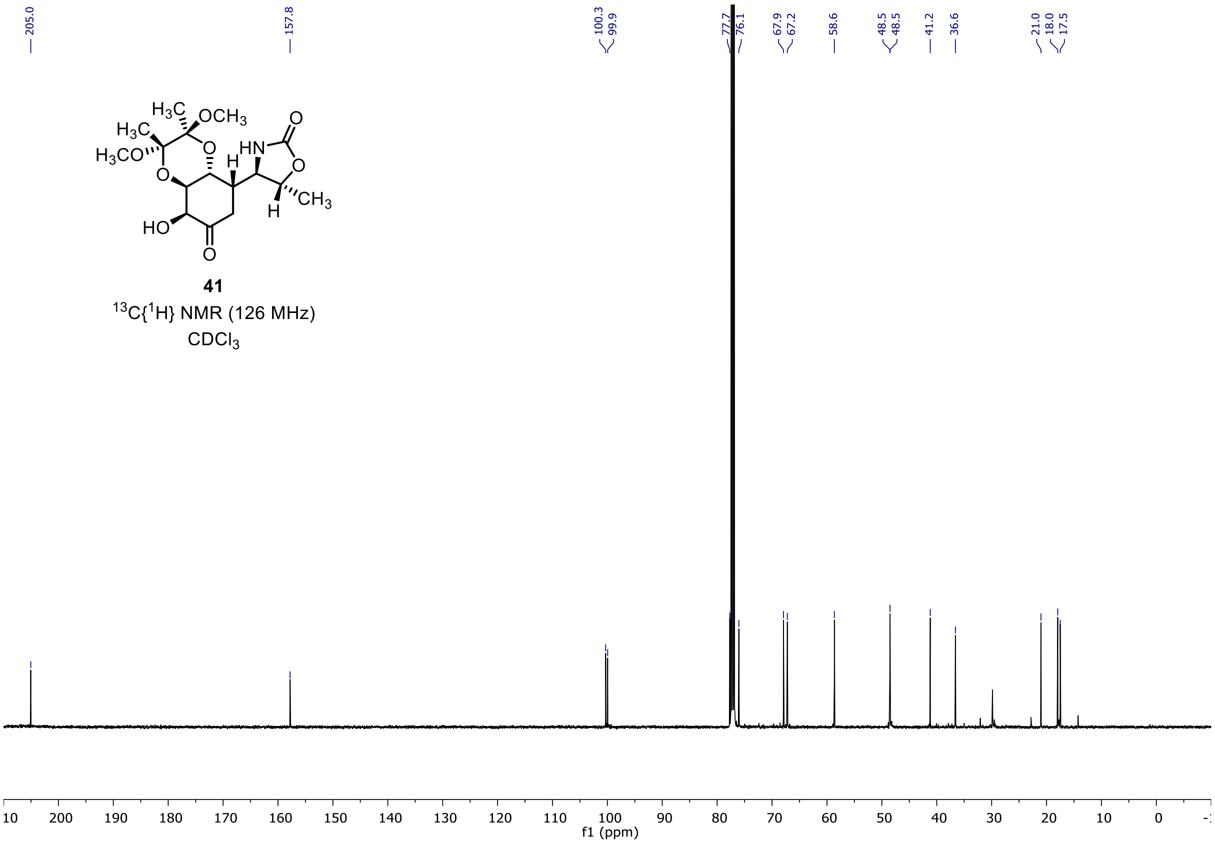

205.0
157.8
100.3
99.9
77.7
76.1
67.9
67.2
58.6
48.5
48.5
41.2
36.6
21.0
18.0
17.5
41
$^{13}C\{^1H\}$ NMR (126 MHz)
$CDCl_3$
f1 (ppm)

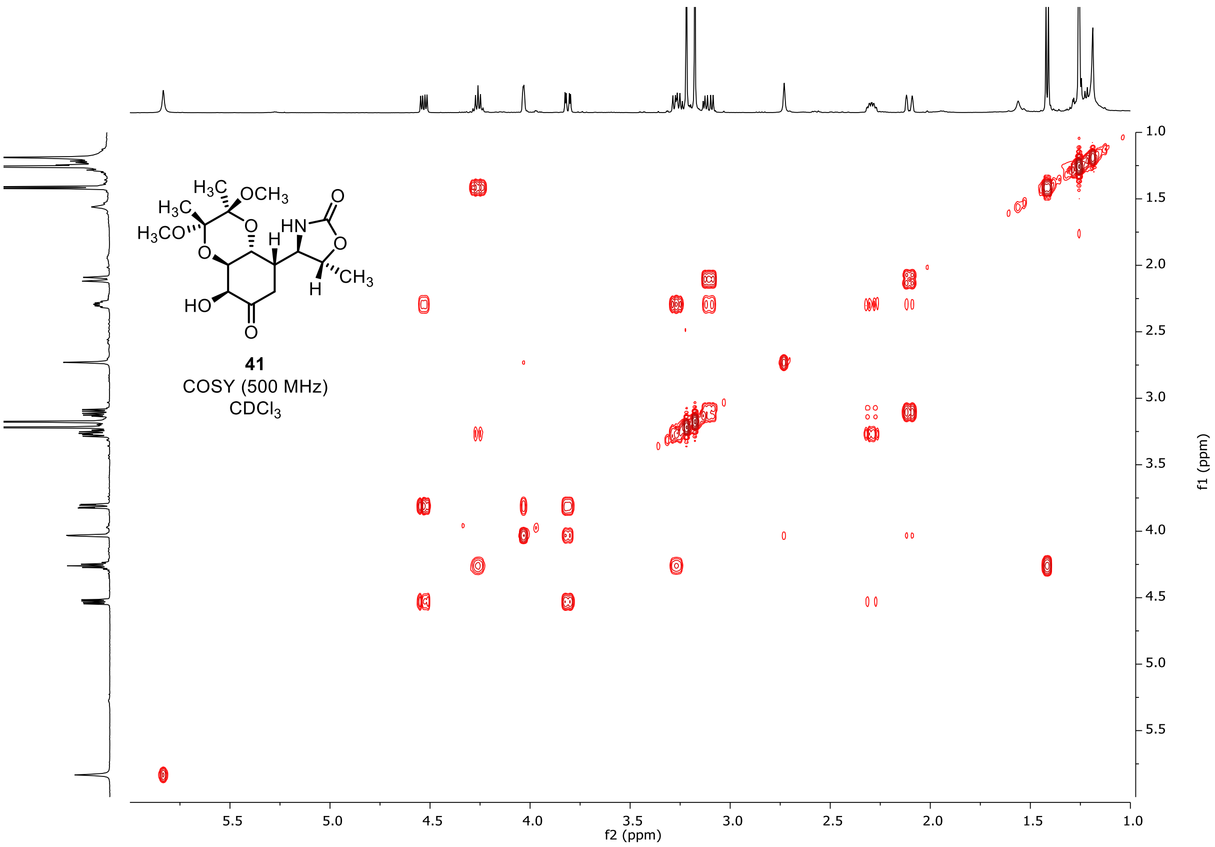

41
COSY (500 MHz)
$CDCl_3$
f2 (ppm)
f1 (ppm)

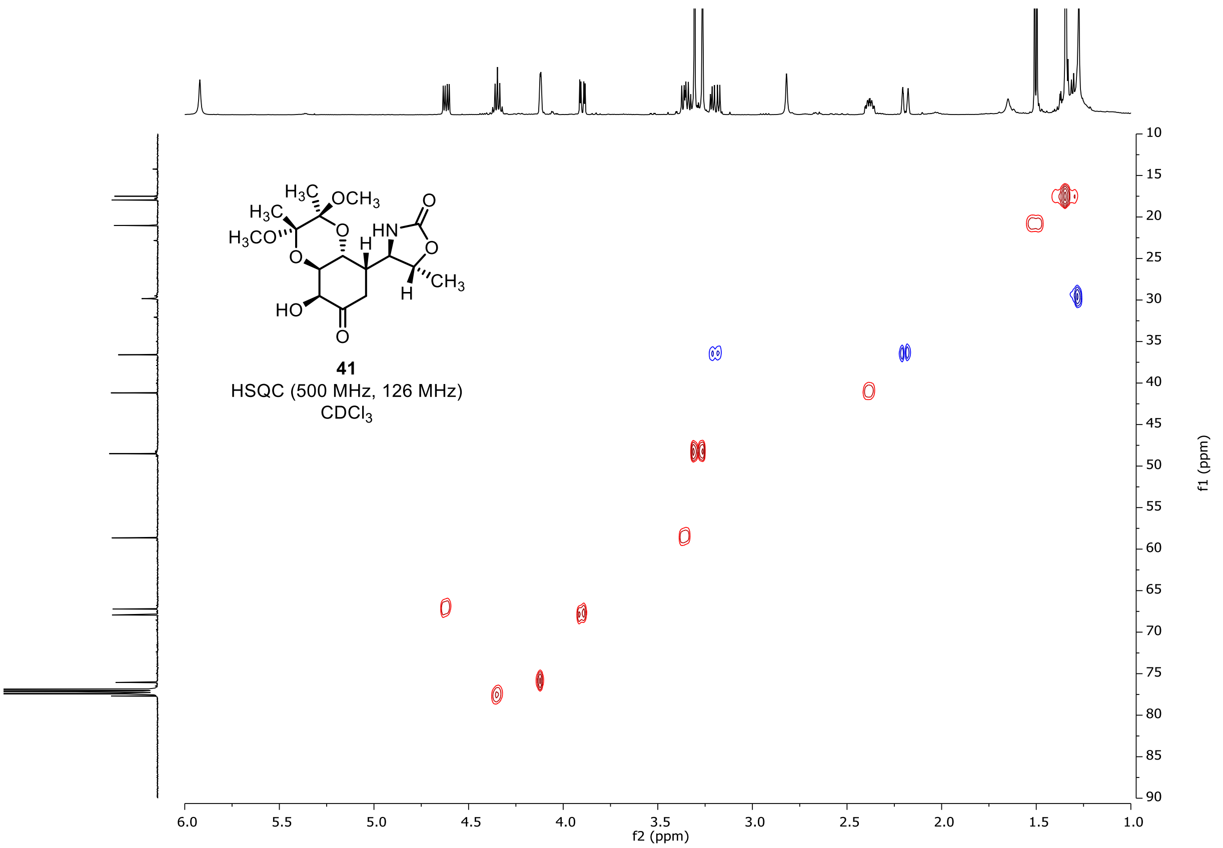
H3C
OCH3
H3C
H3CO
O
HN
O
O
H
O
CH3
H
HO
O
41
HSQC (500 MHz, 126 MHz)
CDCl3
f2 (ppm)
f1 (ppm)

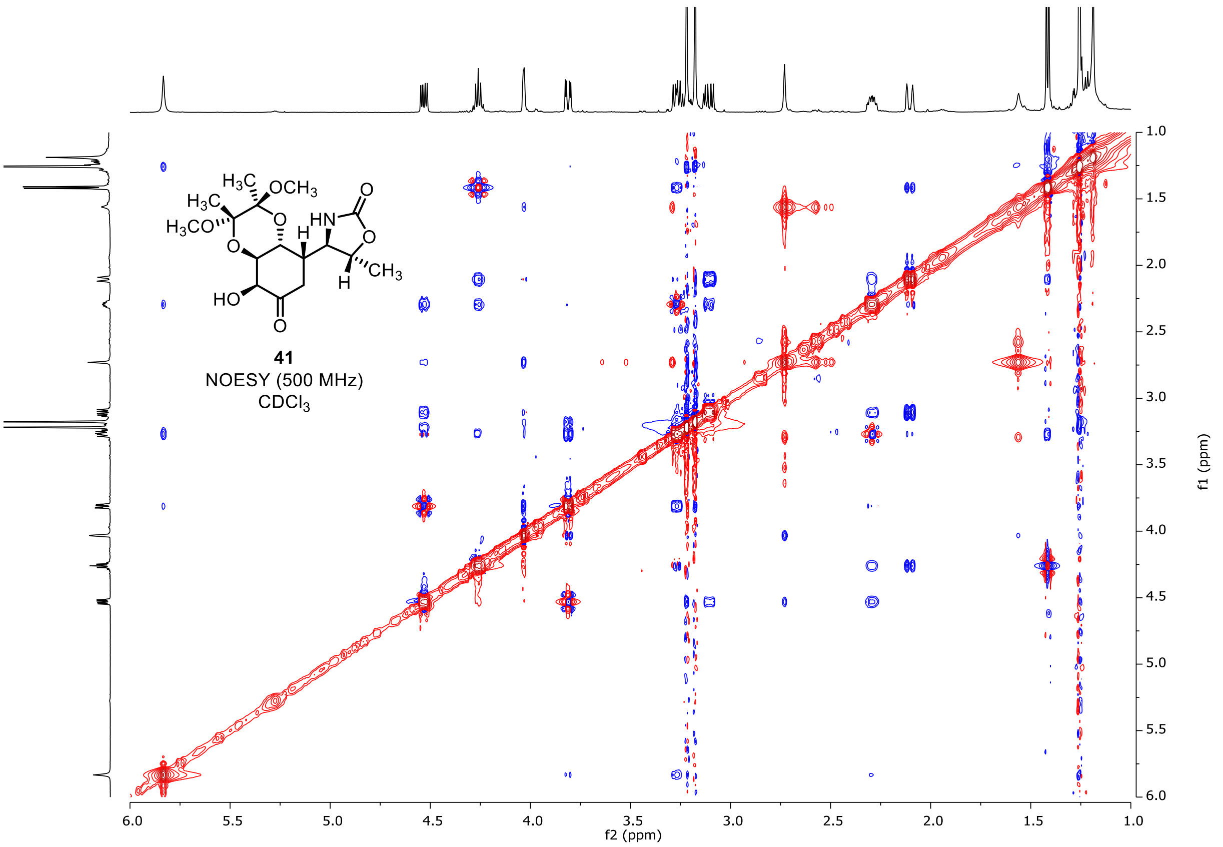
H3C
OCH3
H3C
H3CO
O
HN
O
O
H
O
CH3
H
HO
O
41
NOESY (500 MHz)
CDCl3
f2 (ppm)
f1 (ppm)

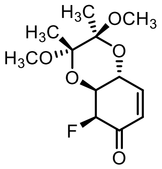

10 + 1,3,5-trimethoxybenzene* (ca. 0.35 equiv)
^{1}H NMR (500 MHz)
DMSO-d_6

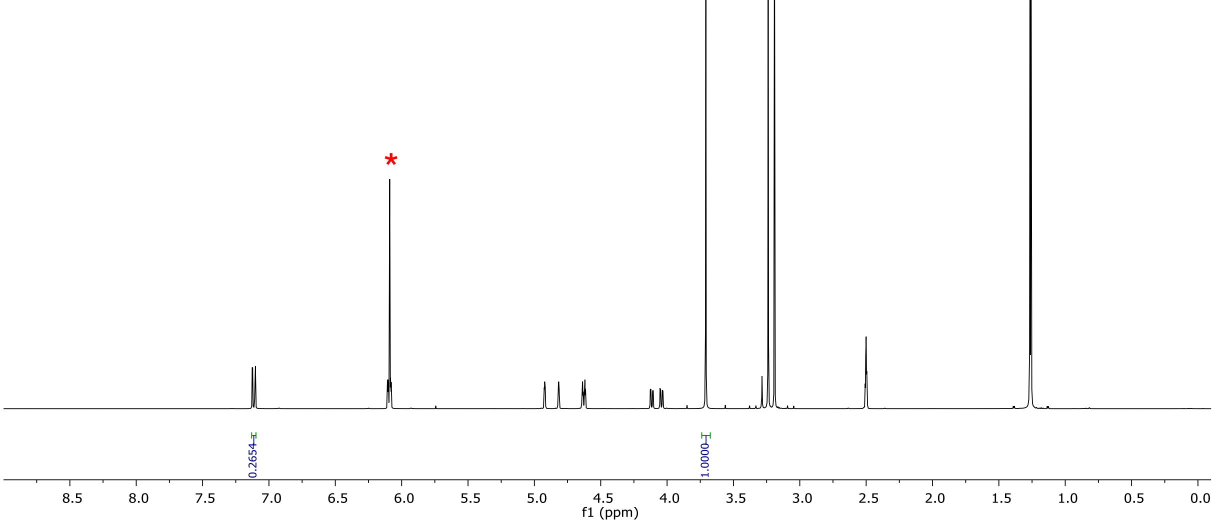

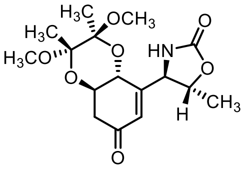

11* + 1,3,5-trimethoxybenzene*
reaction mixture (see Scheme 6, entry 10)
^{1}H NMR (500 MHz)
DMSO-d_6

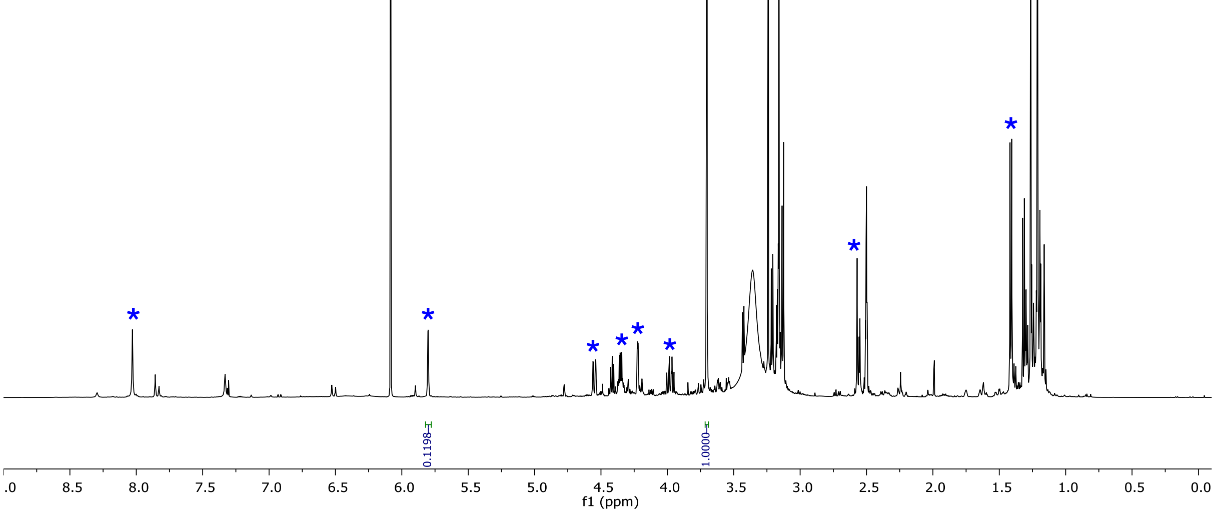

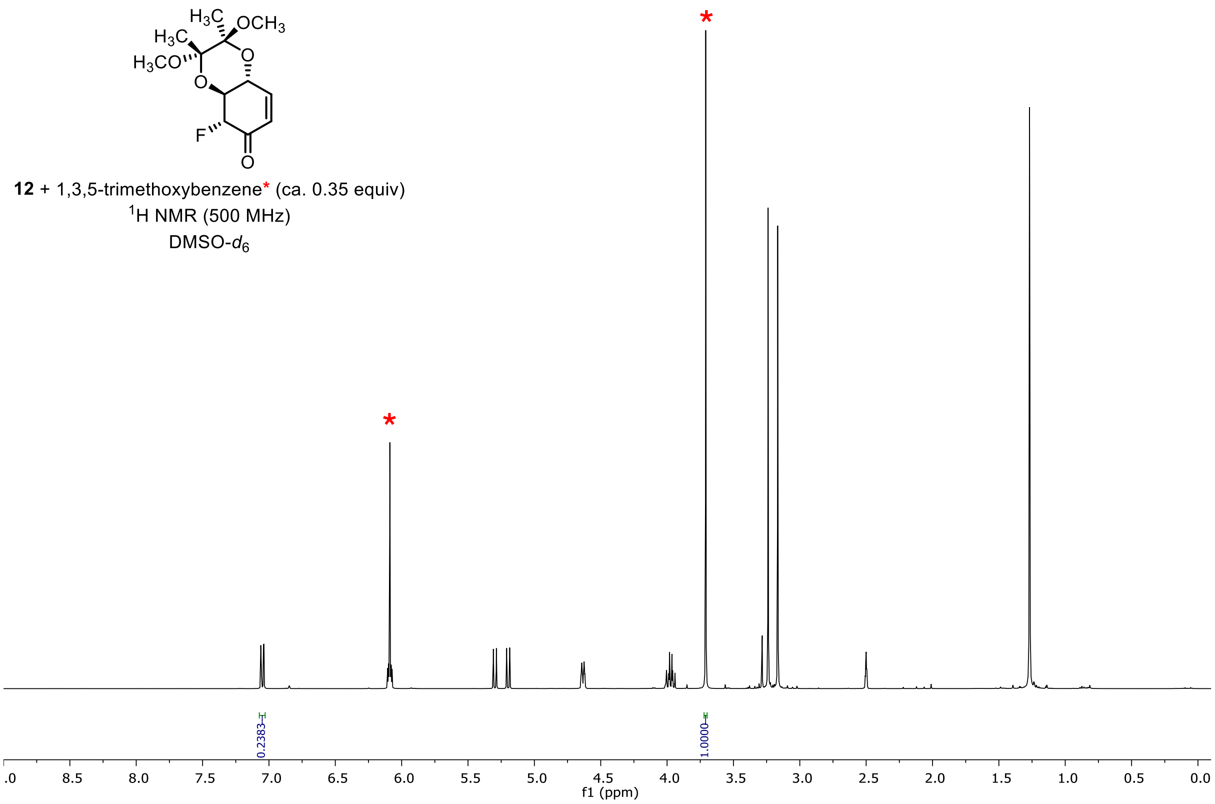

12 + 1,3,5-trimethoxybenzene* (ca. 0.35 equiv)
1H NMR (500 MHz)
DMSO-d6
0.2383
1.0000
f1 (ppm)

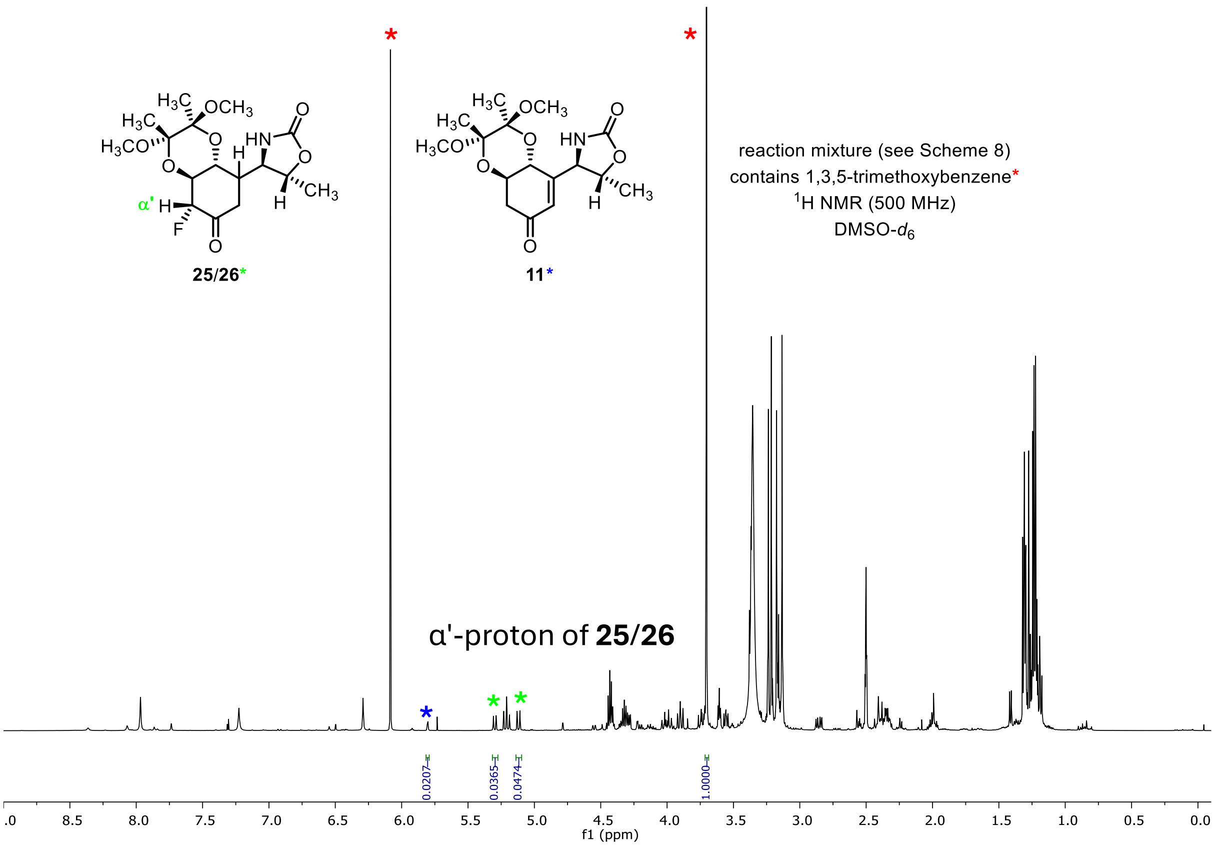

25/26*
11*
reaction mixture (see Scheme 8)
contains 1,3,5-trimethoxybenzene*
1H NMR (500 MHz)
DMSO-d6
α'-proton of 25/26
0.0207
0.0365
0.0474
1.0000
f1 (ppm)

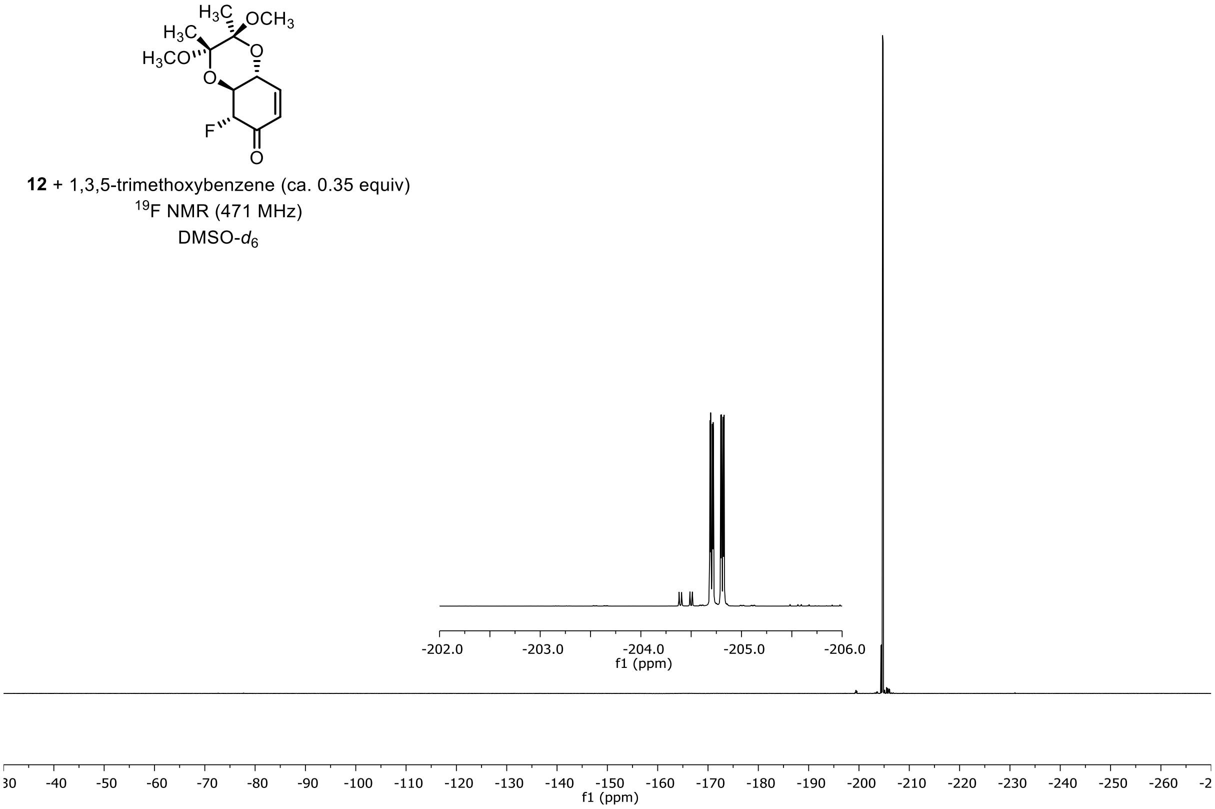
12 + 1,3,5-trimethoxybenzene (ca. 0.35 equiv)
^{19}F NMR (471 MHz)
DMSO-d_6
-202.0 -203.0 -204.0 -205.0 -206.0
f1 (ppm)
-40 -50 -60 -70 -80 -90 -100 -110 -120 -130 -140 -150 -160 -170 -180 -190 -200 -210 -220 -230 -240 -250 -260
f1 (ppm)

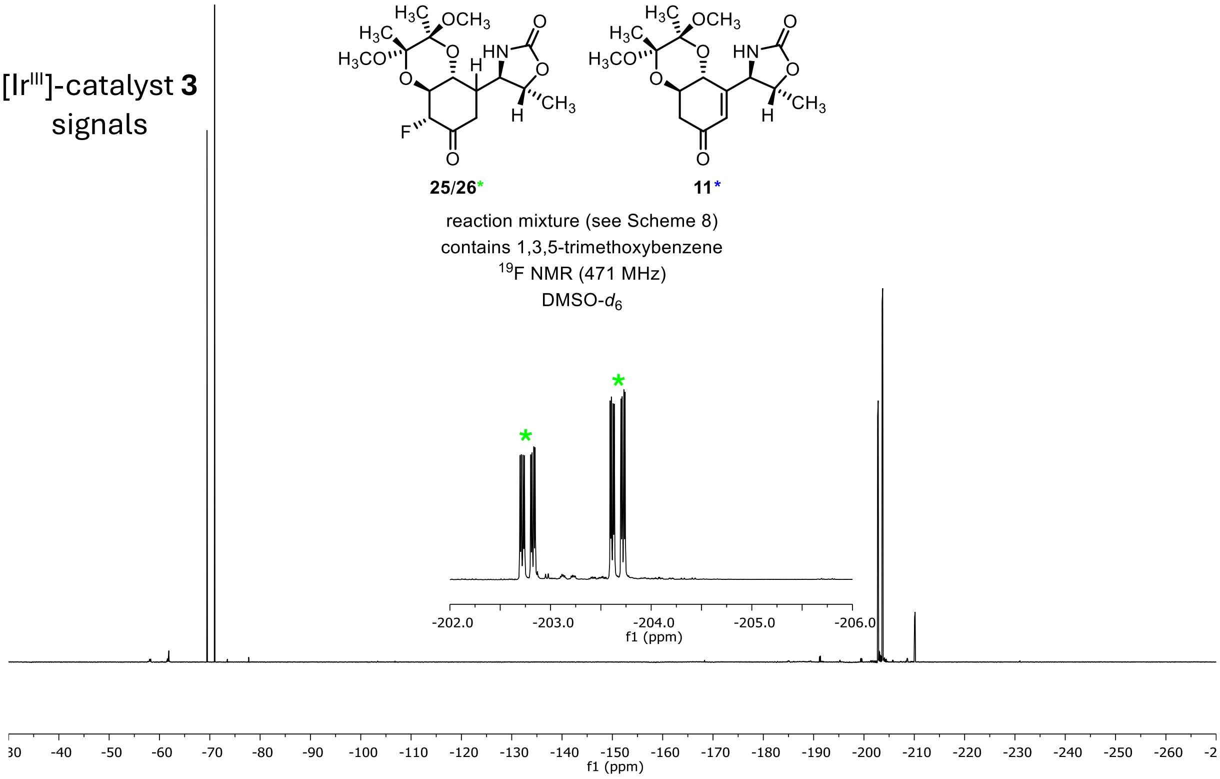
[Ir^{III}]-catalyst **3** signals
25/26*
11*
reaction mixture (see Scheme 8)
contains 1,3,5-trimethoxybenzene
^{19}F NMR (471 MHz)
DMSO-d_6
-202.0 -203.0 -204.0 -205.0 -206.0
f1 (ppm)
-40 -50 -60 -70 -80 -90 -100 -110 -120 -130 -140 -150 -160 -170 -180 -190 -200 -210 -220 -230 -240 -250 -260
f1 (ppm)

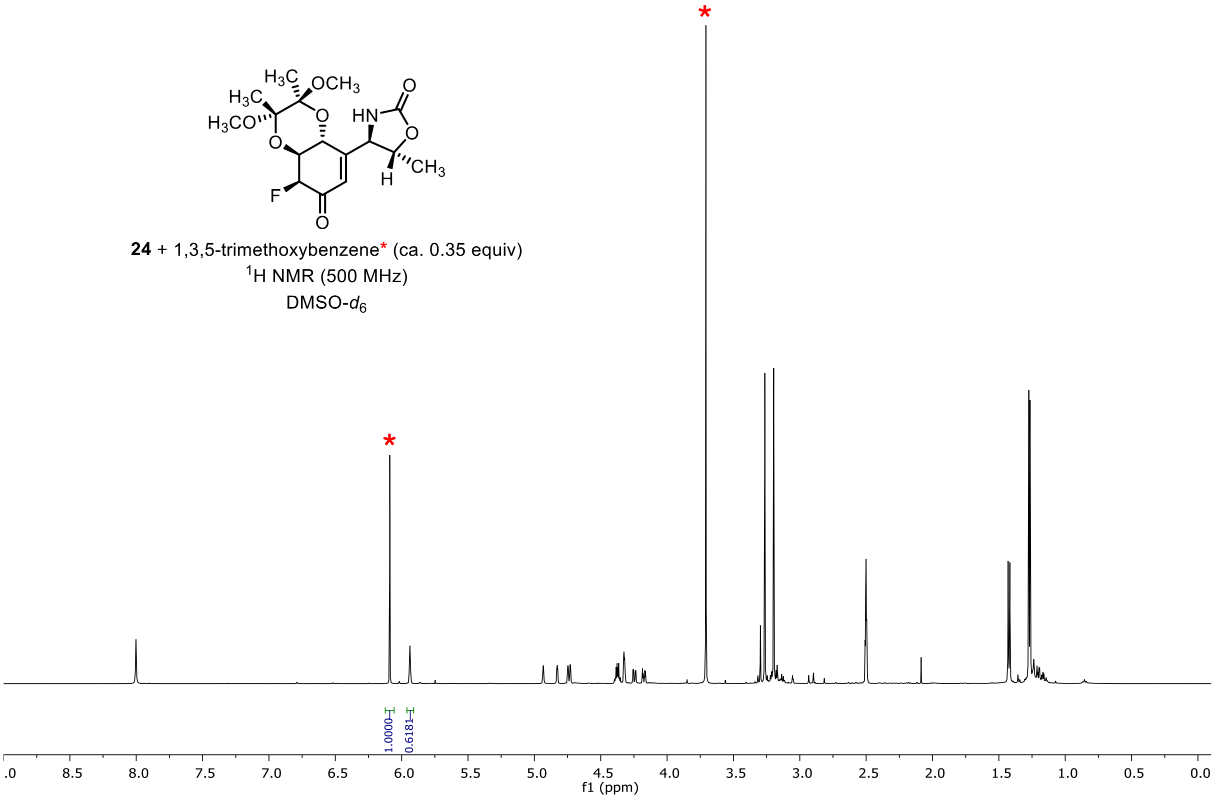
H3C
OCH3
H3C
H3CO
O
O
HN
O
O
CH3
H
F
O
24 + 1,3,5-trimethoxybenzene* (ca. 0.35 equiv)
1H NMR (500 MHz)
DMSO-d6
1.0000
0.6181
f1 (ppm)

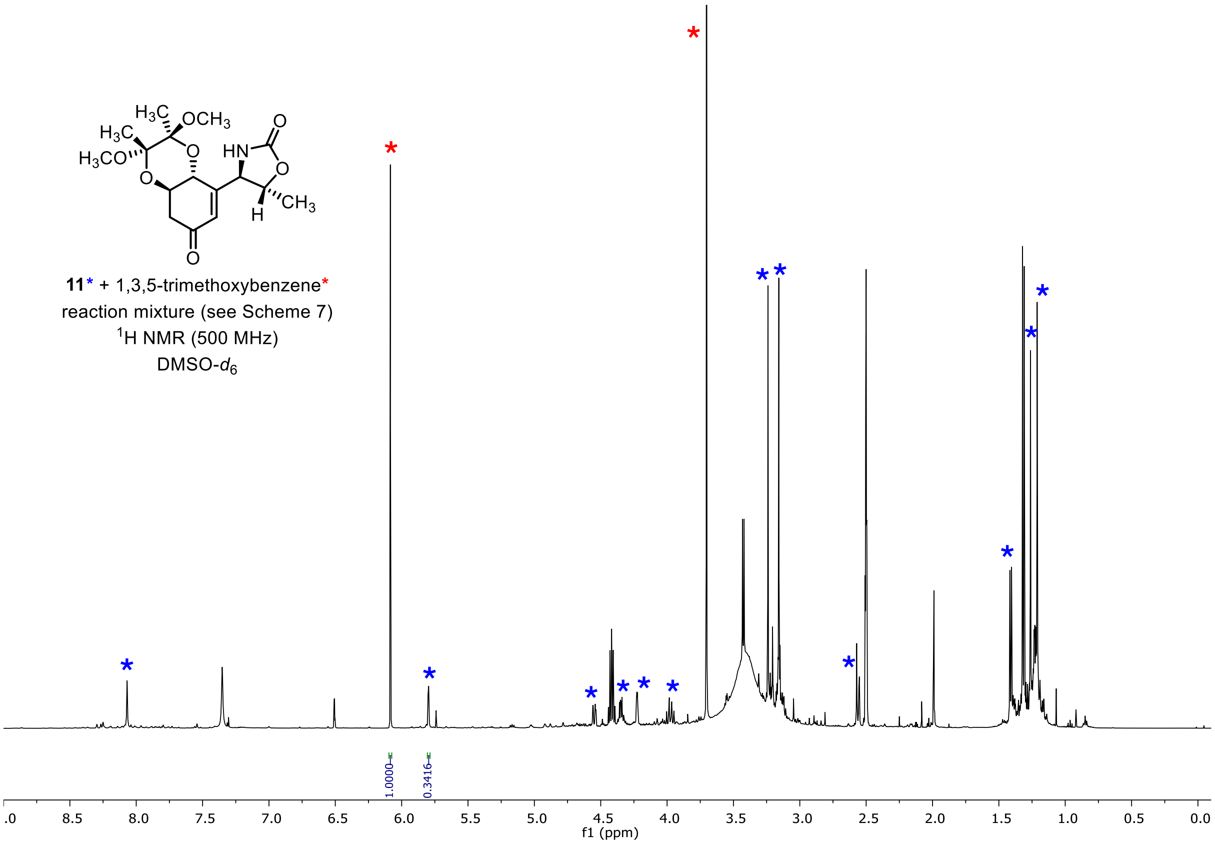
H3C
OCH3
H3C
H3CO
O
O
HN
O
O
CH3
H
O
11* + 1,3,5-trimethoxybenzene*
reaction mixture (see Scheme 7)
1H NMR (500 MHz)
DMSO-d6
1.0000
0.3416
f1 (ppm)

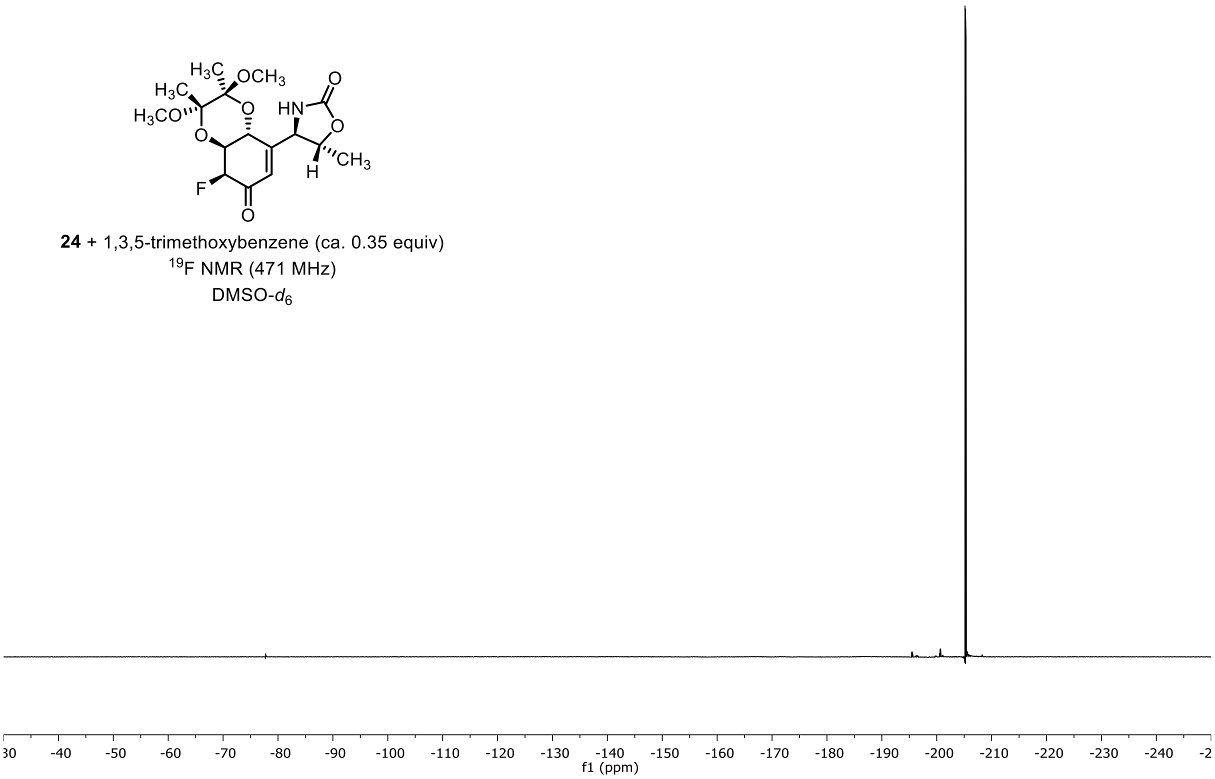

24 + 1,3,5-trimethoxybenzene (ca. 0.35 equiv)
^{19}F NMR (471 MHz)
DMSO-d_6
f1 (ppm)

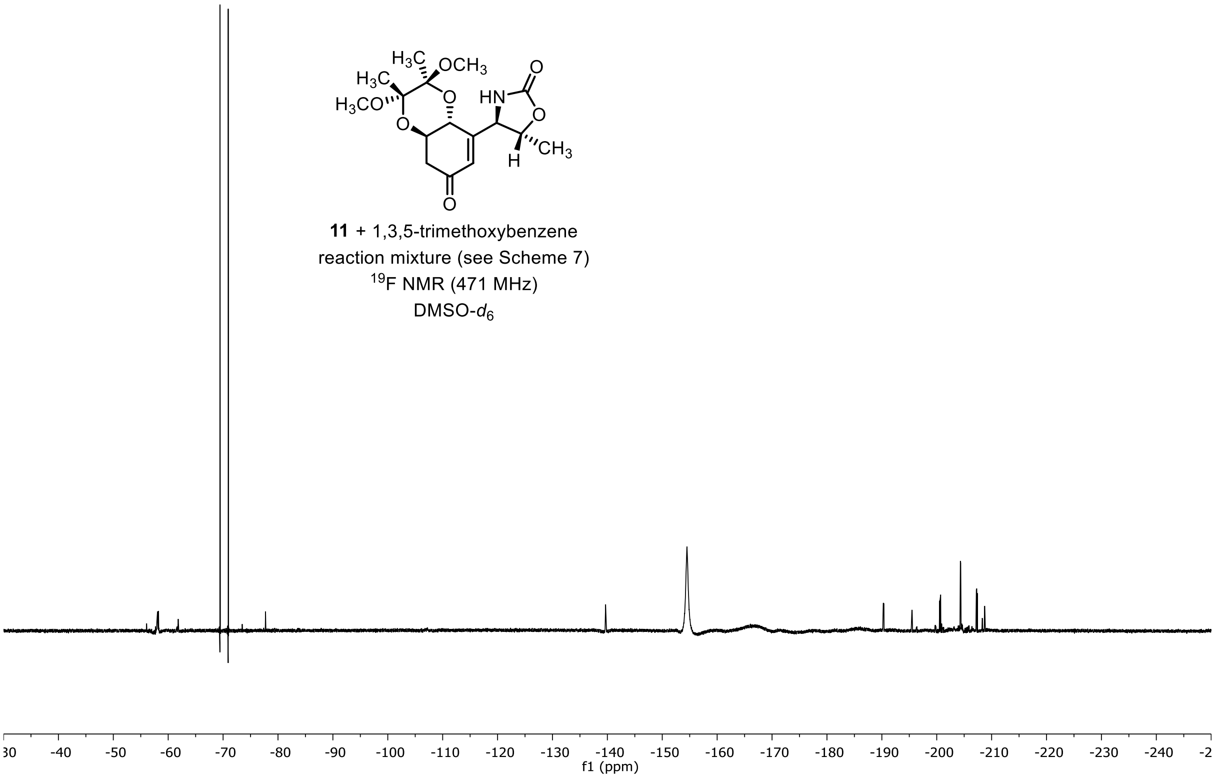

11 + 1,3,5-trimethoxybenzene
reaction mixture (see Scheme 7)
^{19}F NMR (471 MHz)
DMSO-d_6
f1 (ppm)

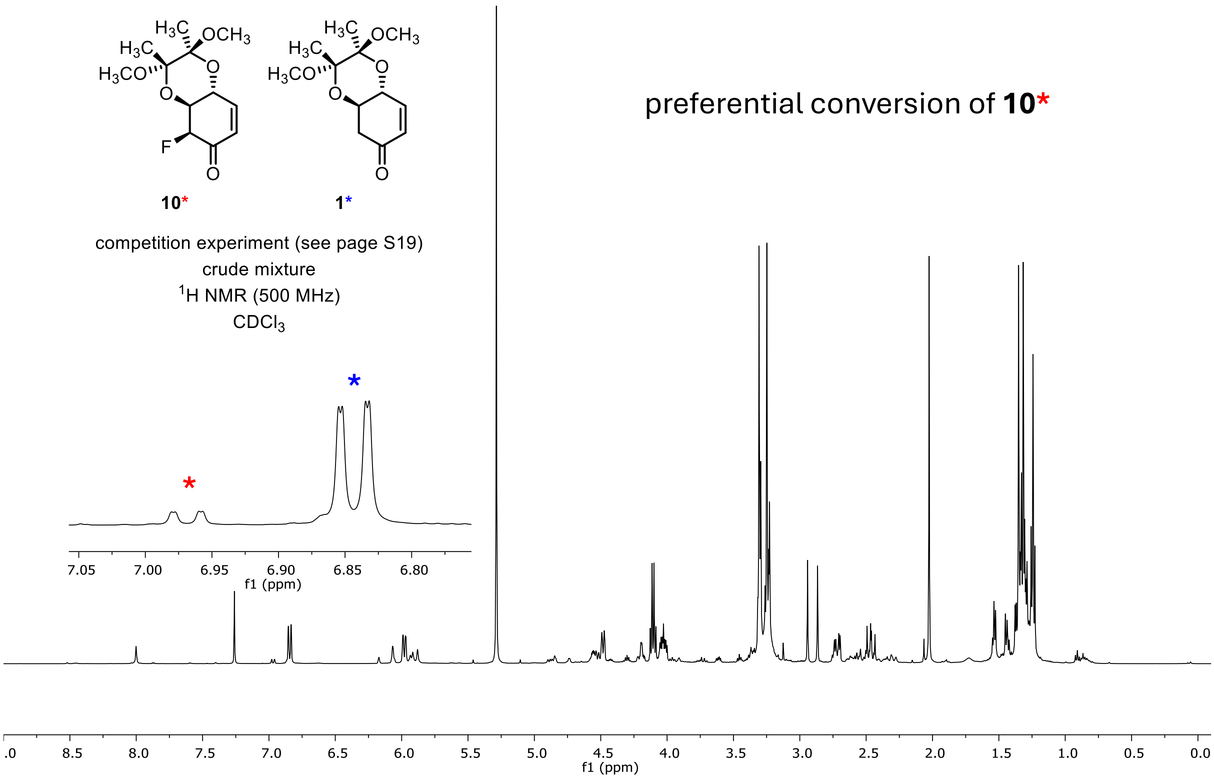

preferential conversion of 10*
10*
1*
competition experiment (see page S19)
crude mixture
1H NMR (500 MHz)
CDCl3
f1 (ppm)

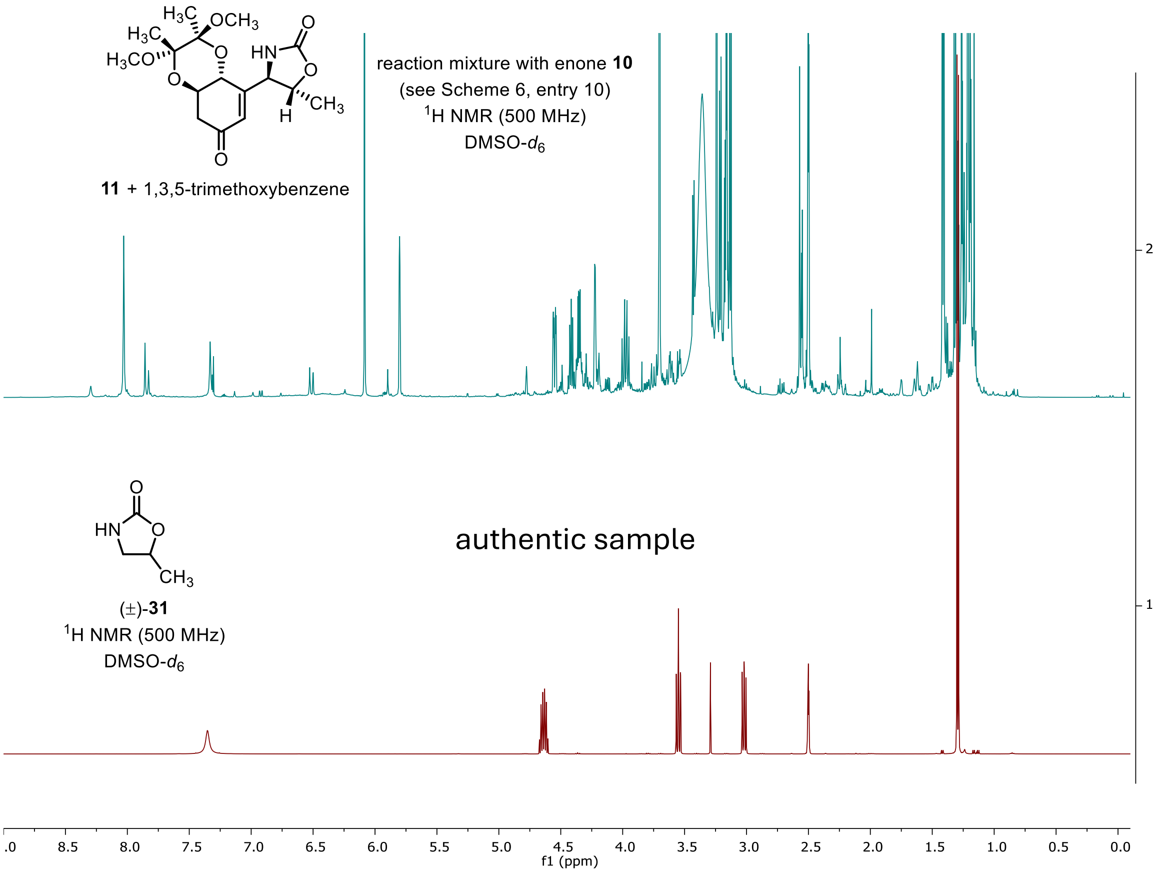

11 + 1,3,5-trimethoxybenzene
reaction mixture with enone 10
(see Scheme 6, entry 10)
^{1}H NMR (500 MHz)
DMSO-d_6
authentic sample
(±)-31
^{1}H NMR (500 MHz)
DMSO-d_6
f1 (ppm)

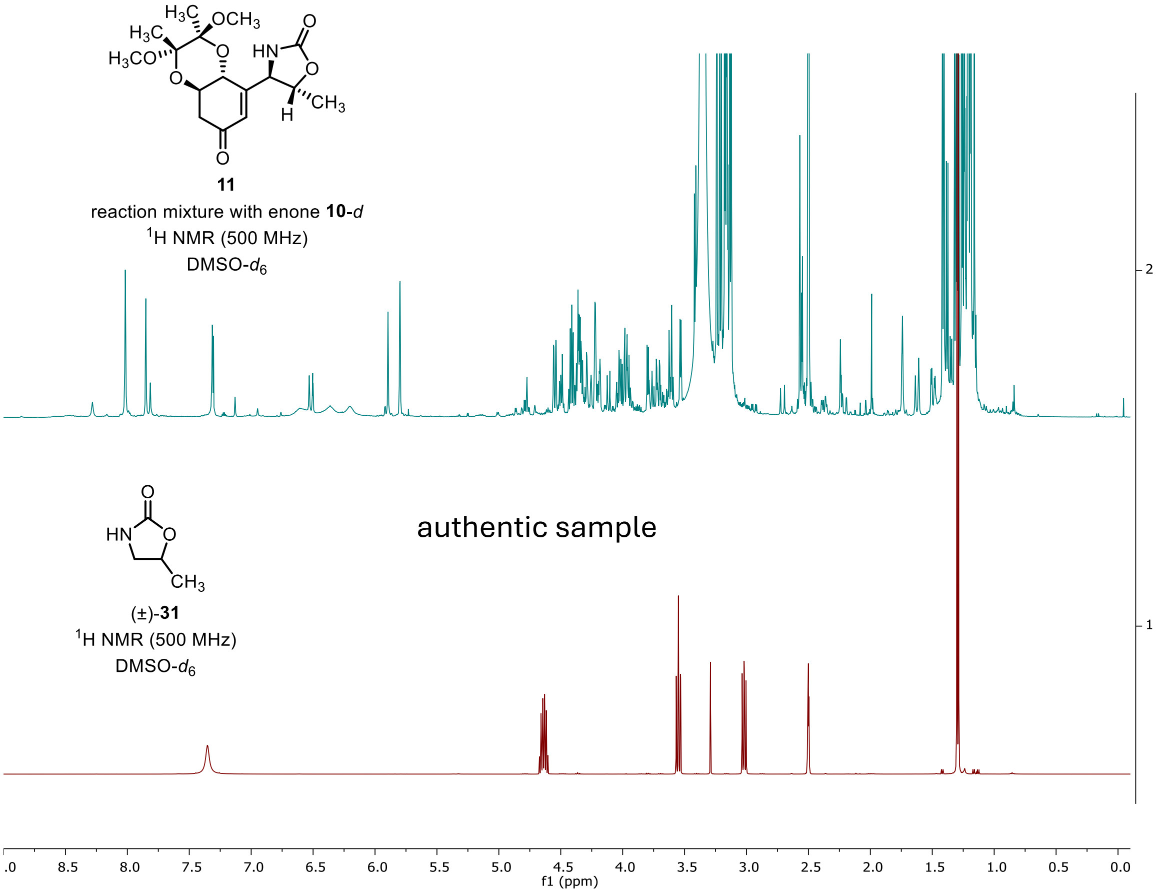

11
reaction mixture with enone 10-d
^{1}H NMR (500 MHz)
DMSO-d_6
authentic sample
(±)-31
^{1}H NMR (500 MHz)
DMSO-d_6
f1 (ppm)
